# The Prevalence of *TET2* Gene Mutations in Patients with *BCR*-*ABL*-Negative Myeloproliferative Neoplasms (MPN): A Systematic Review and Meta-Analysis

**DOI:** 10.3390/cancers13123078

**Published:** 2021-06-20

**Authors:** Yuh Cai Chia, Md Asiful Islam, Phil Hider, Peng Yeong Woon, Muhammad Farid Johan, Rosline Hassan, Marini Ramli

**Affiliations:** 1 Department of Haematology, School of Medical Sciences, Universiti Sains Malaysia, Kubang Kerian 16150, Kelantan, Malaysia; yuhcai@student.usm.my (Y.C.C.); faridjohan@usm.my (M.F.J.); roslin@usm.my (R.H.); 2 Department of Population Health, University of Otago, Christchurch 8140, New Zealand; phil.hider@otago.ac.nz; 3 Department of Molecular Biology and Human Genetics, Tzu Chi University, Hualien 97004, Taiwan; woon07@mail.tcu.edu.tw

**Keywords:** essential thrombocythaemia, meta-analysis, myelofibrosis, myeloproliferative neoplasms, polycythaemia vera, *TET2*

## Abstract

**Simple Summary:**

Many molecular biology techniques have been widely used to study the pathogenesis of different diseases, particularly haematologic malignancies which are generally caused by abnormalities in the genome. *TET2* gene is one of the commonly found mutated genes in *BCR*-*ABL*-negative myeloproliferative neoplasms. However, the prevalence of *TET2* gene mutations in the disease remains unclear. Therefore, this study aims to estimate the prevalence of *TET2* gene mutations in myeloproliferative neoplasms. The findings may be helpful for future research, diagnoses and the identification of better therapeutic strategies to manage the diseases.

**Abstract:**

Multiple recurrent somatic mutations have recently been identified in association with myeloproliferative neoplasms (MPN). This meta-analysis aims to assess the pooled prevalence of *TET2* gene mutations among patients with MPN. Six databases (PubMed, Scopus, ScienceDirect, Google Scholar, Web of Science and Embase) were searched for relevant studies from inception till September 2020, without language restrictions. The eligibility criteria included *BCR*-*ABL*-negative MPN adults with *TET2* gene mutations. A random-effects model was used to estimate the pooled prevalence with 95% confidence intervals (CIs). Subgroup analyses explored results among different continents and countries, WHO diagnostic criteria, screening methods and types of MF. Quality assessment was undertaken using the Joanna Briggs Institute critical appraisal tool. The study was registered with PROSPERO (CRD42020212223). Thirty-five studies were included (*n* = 5121, 47.1% female). Overall, the pooled prevalence of *TET2* gene mutations in MPN patients was 15.5% (95% CI: 12.1–19.0%, *I*^2^ = 94%). Regional differences explained a substantial amount of heterogeneity. The prevalence of *TET2* gene mutations among the three subtypes PV, ET and MF were 16.8%, 9.8% and 15.7%, respectively. The quality of the included studies was determined to be moderate–high among 83% of the included studies. Among patients with *BCR*-*ABL*-negative MPN, the overall prevalence of *TET2* gene mutations was 15.5%.

## 1. Introduction

Myeloproliferative neoplasms (MPN) are a group of rare blood cancers characterised by the clonal expansion of a large number of abnormal haematopoietic stem cells. Classic Philadelphia-negative (*BCR*-*ABL*-negative) MPN can be divided into three categories: (i) polycythaemia vera (PV), (ii) essential thrombocythaemia (ET) and (iii) primary myelofibrosis (PMF). MPN can transform into acute myeloid leukaemia (AML) and may be associated with an elevated risk of thrombotic and haemorrhagic events [1,2]. Thrombosis and haemorrhage are the major causes of mortality and morbidity amongst patients with MPN and occur in about 34–39% of cases with PV, 10–29% with ET and 7.2–13.2% of patients with PMF [3].

Three main driver gene mutations, Janus kinase 2 (*JAK2*), Thrombopoietin receptor (*MPL*) and Calreticulin (*CALR*), have been identified in association with MPN and may have an important role in assisting the diagnosis of MPN [4]. In addition, epigenetic modification genes such as *TET2*, *ASXL1*, *DNMT3A* and *EZH2* are also commonly mutated in cases of MPN with a frequency of 1–30% [5,6,7,8].

*TET2* participates in one of the crucial steps in gene regulation, and mutations in this gene have been identified in 5–20% of people diagnosed with MPN [9]. Somatic missense mutations, somatic nonsense mutations and insertion–deletion mutations are detected in the *TET2* gene among MPN patients. All of these mutations are loss-of-function mutations. Malfunction of *TET2* protein may lead to the development of MPN and contributes to the disease progression [10,11]. However, some disagreement still exists about the relative significance of these *TET2* gene mutations to MPN. Some researchers suggest that *TET2* gene mutations are not important for MPN [12,13], whereas others have concluded that these mutations significantly contribute to their phenotype [14,15].

The prevalence of *TET2* gene mutations among MPN has not yet been established. This meta-analysis aims to estimate the prevalence of *TET2* gene mutations among all *BCR*-*ABL*-negative MPN and its three main subtypes.

## 2. Materials and Methods

PRISMA guidelines [16] were followed, and a study protocol was registered at the International Prospective Register of Systematic Reviews (PROSPERO), registration number CRD42020212223.

### 2.1. Data Sources and Searches

PubMed, Scopus, ScienceDirect, Google Scholar, Web of Science and Embase databases were searched from their inception till September 2020, without any language restrictions. Detailed search strategies are presented in Table S1. Any published studies or preprints with relevant data were included. Review articles, case reports and opinion articles were excluded. Data presented on websites or reported by press releases and news reports were not considered. Snowball searching was employed to review the references of included studies. Endnote X8 software was used to remove duplicate studies.

### 2.2. Study Selection

Study eligibility was determined by screening the title and abstract of the articles of interest. Two authors (Y.C.C. and M.A.I.) independently examined full-text reports of potentially relevant studies for inclusion. Any disagreements were resolved by consensus.

### 2.3. Extraction of Data

Data were independently extracted by two authors (Y.C.C. and M.A.I). The following data were obtained from each eligible study and inserted into a customised Excel spreadsheet: author surname, publication year, study design, study location, type of MPN, number of patients with MPN, demographic characteristics of patients including age and sex, clinical characteristics of the MPN patients including haemoglobin level, leucocyte and platelet counts, the total number of mutated *ASXL1* and the screening method used to identify *TET2* gene mutations and diagnostic criteria employed for MPN diagnoses.

### 2.4. Quality Assessment

A random-effects model was used to estimate the pooled prevalence of the *TET2* gene mutations amongst patients with MPN, including 95% confidence intervals (Cis). Two authors (Y.C.C. and M.A.I.) independently assessed the quality of included studies using the Joanna Briggs Institute critical appraisal tools [13]. Study quality was categorised into three groups: low-quality or high risk of bias, moderate quality or moderate risk of bias, and high-quality or low risk of bias with overall scores of <50%, 50–69% and ≥70%, respectively [17].

### 2.5. Publication Bias

Funnel plots presenting estimates of prevalence plotted against standard error measures were used to assess the likelihood of publication bias. When a minimum of 10 studies were available, an Egger’s test was conducted to assess publication bias based on funnel plot asymmetry.

### 2.6. Data Synthesis and Sensitivity Analysis

The *I*^2^ statistic was used to gauge the heterogeneity between studies, with *I*^2^ > 75% indicating substantial heterogeneity. The statistical significance of study heterogeneity was also assessed using Cochran’s Q test; *p* < 0.05 was considered statistically heterogeneous. To help identify the outlier studies and the sources of heterogeneity, a Galbraith plot was constructed. Prevalence estimates were explored with sensitivity analyses. Three strategies were followed for these analyses: (i) studies with small sample sizes (<100) were excluded, (ii) low-quality studies were excluded and (iii) outlier studies were excluded. In each case, the results were then compared to the overall prevalence estimate. Metaprop codes in meta (version 4.15-1) and metaphor (version 2.4-0) packages of R (version 3.6.3) and RStudio (version 1.3.1093) were used for the analyses and graphs [18].

## 3. Results

### 3.1. Study Selection

**Figure 1:**
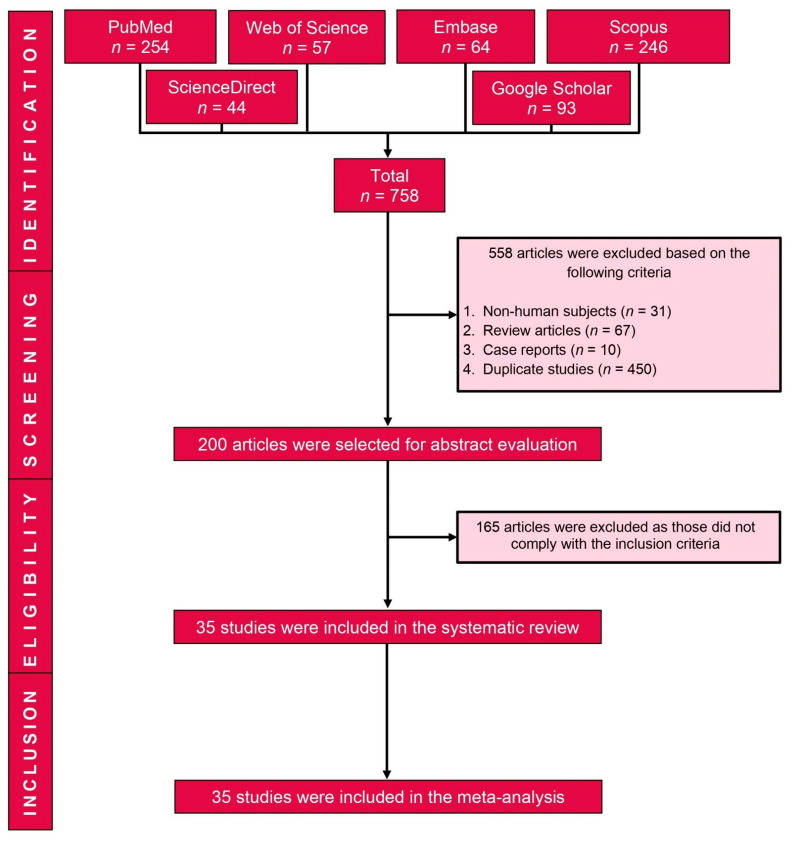
PRISMA flow diagram of study selection.

The search generated 758 potentially relevant studies. After excluding 558 studies (duplicates *n* = 450; review articles *n* = 67; non-human studies *n* = 31; and case reports, *n* = 10), 200 full-text studies were examined and 35 studies met the inclusion criteria and were included in the review (Figure 1).

### 3.2. Characteristics of Included Studies

Table 1 presents the main characteristics of the 35 included studies. Overall, the meta-analysis includes data from 5121 patients with MPN (47.1% female). Study participants were located in four continents: Europe (*n* = 1758), Asia (*n* = 301), North America (*n* = 3019) and Australia (*n* = 43), and 12 countries (Australia, China, Denmark, France, Germany, Italy, Korea, Spain, Sweden, Switzerland, the United Kingdom and the United States of America). Most (27/35) studies used a version of the World Health Organization classification and diagnostic criteria (WHO 2016 7 studies, WHO 2008 17 studies and WHO 2001 3 studies) to determine MPN diagnoses. Many studies confirmed *TET2* gene mutations with either next-generation sequencing (NGS) or Sanger sequencing, which have higher sensitivity in detecting mutations compared with other methods, such as high-resolution melting (HRM) analysis [19]. One study was published in Chinese Mandarin and was translated into English (Y.C.C.).

### 3.3. Meta-Analysis

**Figure 2:**
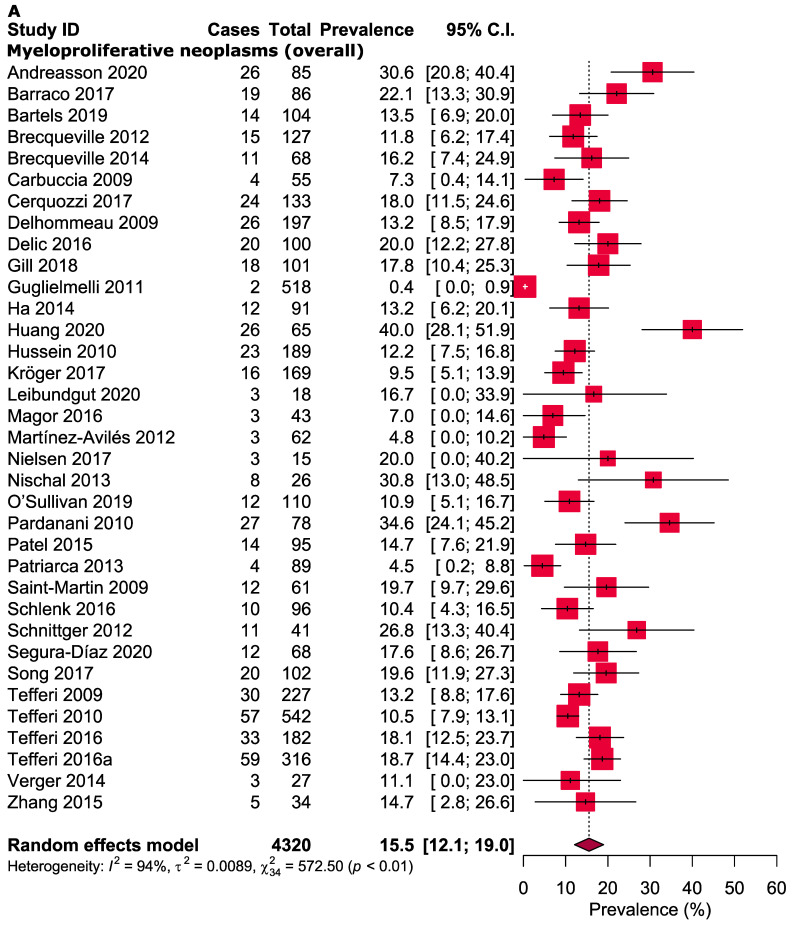

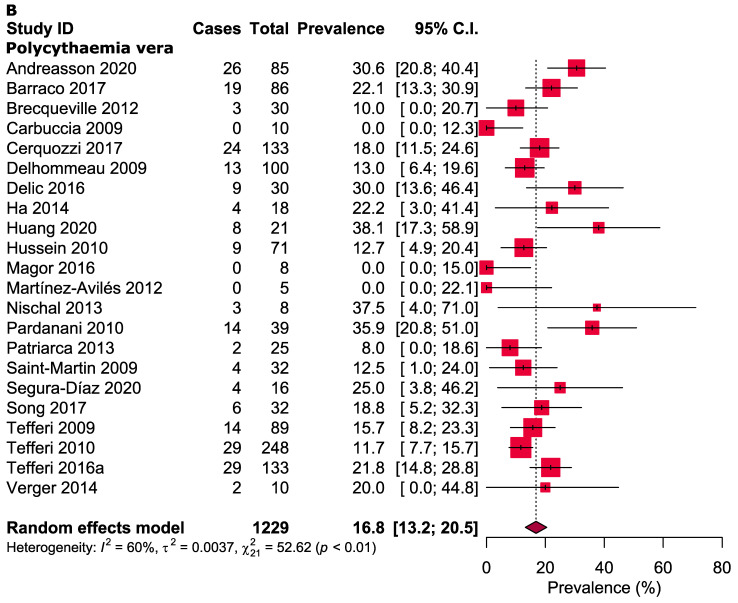

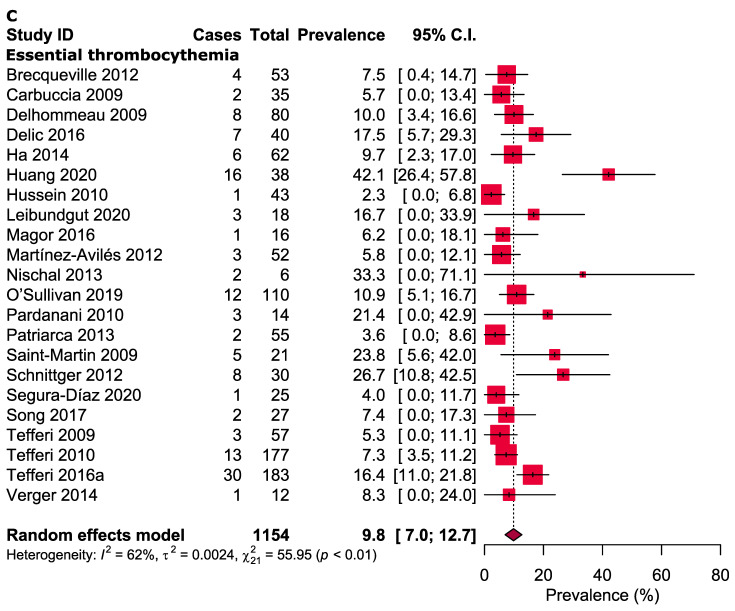

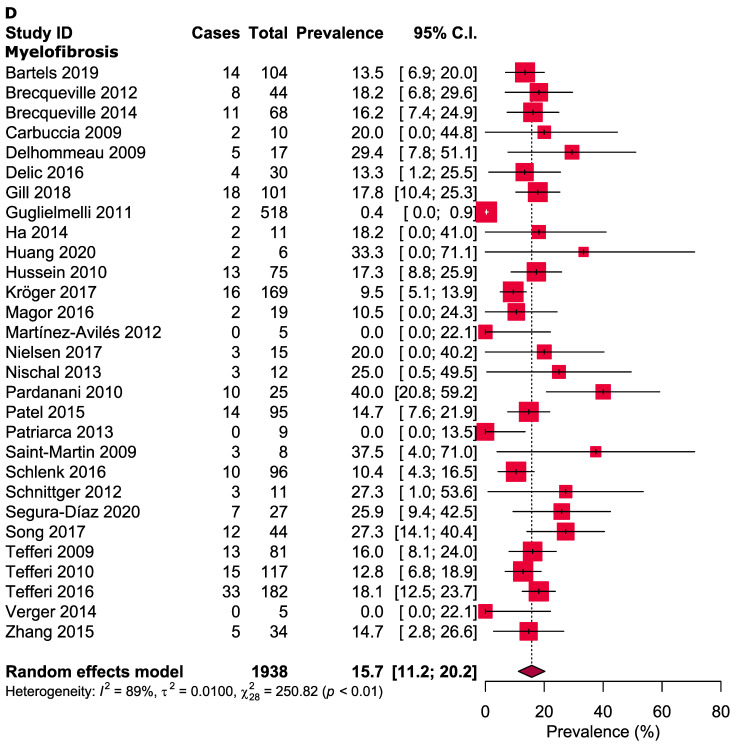
(**A**) Prevalence of *TET2* gene mutations in patients with MPN (overall). (**B**) Prevalence of *TET2* gene mutations in patients with PV. (**C**) Prevalence of *TET2* gene mutations in patients with ET. (**D**) Prevalence of *TET2* gene mutations in patients with MF.

The overall pooled prevalence of *TET2* gene mutations in patients with MPN was 15.5% (95% CI: 12.1–19.0%, *I*^2^ = 94%, Figure 2A). The prevalence of *TET2* gene mutations in PV, ET and MF patients was 16.8% (95% CI: 13.2–20.5%, *I*^2^ = 60%, Figure 2B), 9.8% (95% CI: 7.0–12.7%, *I*^2^ = 62%, Figure 2C) and 15.7% (95% CI: 11.2–20.2%, *I*^2^ = 89%, Figure 2D), respectively. In other subgroup analyses, the pooled prevalence of *TET2* gene mutations was compared between four continents: Europe (13.0%; 95% CI: 8.8–17.2%, *I*^2^ = 92%), North America (17.4%; 95% CI: 14.0–20.9%, *I*^2^ = 74%), Asia (20.8%; 95% CI: 10.5–31.1%, *I*^2^ = 80%) and Australia (7.0%; 95% CI: 0.0–14.6%, *I*^2^ = NA). The prevalence of *TET2* gene mutations were further analysed based on countries: China (23.9%; 95% CI: 9.6–38.1%, *I*^2^ = 82%), France (13.6%; 95% CI: 10.6–16.7%, *I*^2^ = 0%), Germany (14.2%; 95% CI: 9.2–19.1%, *I*^2^ = 61%), Italy (1.9%; 95% CI: 0.0–5.7%, *I*^2^ = 71%), Spain (10.7%; 95% CI: 0.0–23.2%, *I*^2^ = 82%) and the United States (17.4%; 95% CI: 14.0–20.9%, *I*^2^ = 74%). Assessments of PV, ET and MF prevalence across the four continents (Figure S1) and in relation to different countries were also examined (Figure S2). Three forms of WHO criteria were used and the prevalence of *TET2* gene mutations was highest in the 2016 version (WHO 2001 criteria 12.9%, 95% CI: 10.2–15.5%, *I*^2^ = 0%, WHO 2008 criteria 14.5%, 95% CI: 9.7–19.3%, *I*^2^ = 95% and WHO 2016 criteria 20.1%, 95% CI: 14.7–25.4%, *I*^2^ = 61%) (Figure S3). A higher prevalence of *TET2* gene mutations were observed while using NGS (17.2%, 95% CI: 14.0–20.4%, *I*^2^ = 80%) and Sanger sequencing (12.7%, 95% CI: 9.6–15.9%, *I*^2^ = 52%), but not in HRM analysis (7.7%, 95% CI: 0.0–16.6%, *I*^2^ = 88%) (Figure S4). The MF subgroup was further divided into two subgroups (PMF and SMF), and the prevalence of *TET2* gene mutations were studied in both and found to be similar (PMF 16.7%, 95% CI: 13.6–19.8%, *I*^2^ = 24% and SMF 14.8%, 95% CI: 9.3–20.2%, *I*^2^ = 0%) (Figure S5). Various levels of heterogeneity were observed in the main analyses (ranging from 60% to 94%) (Figure 2) and subgroup analyses (ranging from 0% to 93%) (Table 2, Figures S1–S5).

### 3.4. Quality Assessment

Detailed quality assessments of the included studies are presented in Tables S2 and S3. Most studies were judged to be of high quality (68.6%), while the remainder were considered to be of either moderate (14.3%) or low quality (17.7%).

### 3.5. Publication Bias

**Figure 3:**
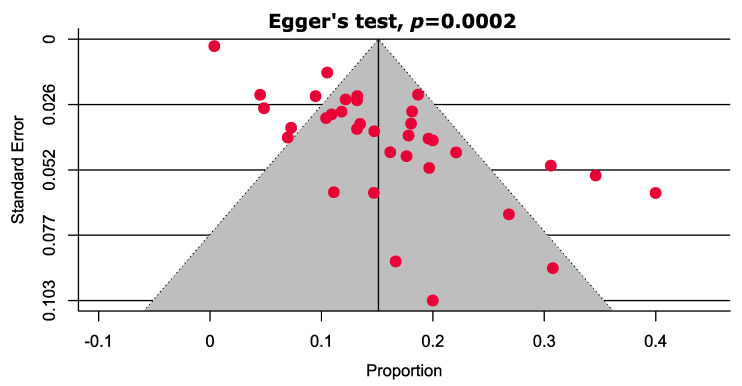
Funnel plot estimating the prevalence of *TET2* gene mutations in patients with MPN (overall).

The results from the funnel plots and Egger’s tests suggest that there is only a small likelihood of publication bias (Figure 3 and Figure S6).

### 3.6. Sensitivity Analyses

**Figure 4:**
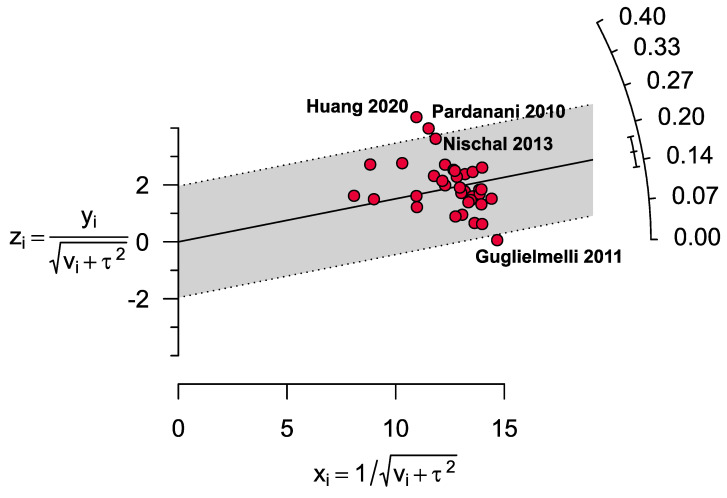
Galbraith plot analysing MPN (overall) identified four outlier studies.

In the sensitivity analyses, only minor differences (ranging from 4.0% lower to 1.8% higher) were observed in the pooled prevalence estimates of *TET2* gene mutations among cases of MPN compared to the main findings (Table 3 and Figure S7). A Galbraith plot was performed, and four outlier studies [29,30,37,39] were identified (Figure 4).

## 4. Discussion

The overall prevalence of *TET2* gene mutations among *BCR*-*ABL*-negative MPN patients was estimated to be 15.5%. This estimate is similar to the occurrence of *TET2* somatic mutations in patients with various myeloid cancers [11]. Compared with other myeloid malignancies, the prevalence of *TET2* gene mutations among patients with *BCR*-*ABL*-negative MPN appears to be lower. Among patients with myelodysplastic syndromes (MDS), the prevalence of these mutations has been estimated to be 18–35% [11,53,54,55,56,57,58], 36–60% for those with chronic myelomonocytic leukaemia (CMML) [54,59,60,61], 24% in cases with AML, 22% in chronic myelogenous leukaemia (CML) [11], 20% in mastocytosis [62,63] and about 30% in patients with blastic plasmacytoid dendritic cell neoplasm [64,65].

The results appear to confirm the observation that epigenetic regulators like the *TET2* gene have mutated more frequently among those patients with PV (*p* = 0.05), compared with those with either MF (*p* = 0.02) or ET (*p* = 0.023) [27]. According to a meta-analysis that analysed the frequency of three main genes (*JAK2*, *MPL* and *CALR*) in MPN from 2000 to 2018 [66], for the most common gene mutation *JAK2* V617F, *TET2* showed a lower prevalence as compared to JAK2 V617F in PV (46.7–100.0%), ET (31.3–72.1%) and MF (25.0–85.7%). For the *MPL* gene, our results displayed a higher proportion in PV (16.8% vs. 0%) but similar percentages in ET (9.8% vs. 0.9–12.5%) and MF (15.7% vs. 0–17.7%). Finally, in relation to the last common driver gene, *CALR*, *TET2* manifested a higher prevalence in PV (16.8% vs. 0%), a lower prevalence in ET (9.8% vs. 12.6–50%) and a similar proportion in MF (15.7% vs. 10–100%). From these results, it appears that the *TET2* gene mutations have distributed more evenly across MPN subcategories in contrast to the three main driver mutation genes [47].

This study has several notable strengths. To our knowledge, no meta-analysis has previously investigated the prevalence of *TET2* gene mutations in patients with MPN. This meta-analysis included studies from six databases using robust search strategies without any language restrictions. All the sensitivity analyses produced similar results to the overall findings, suggesting that the main result is likely to be robust and credible.

Nevertheless, there are a few limitations. Several meta-analyses exhibited high heterogeneity, indicating considerable variability among the results from the included studies. After excluding the four outlier studies identified by the Galbraith plot, heterogeneity was reduced from 94% to 63% across all MPN studies, 60% to 54% for PV, 62% to 48% for ET and 89% to 18% for MF, suggesting that these four studies were an important source of heterogeneity. Several factors may further explain this heterogeneity. One of the outlier studies [29] recorded a very low prevalence of *TET2* gene mutations (0.4%). This may be due to the use of a different method (HRM analysis) that may be less sensitive to identifying the mutations compared with most other studies. Notably, a similar result was also observed in one of the two other studies [35,44] that also employed the same analytical technique. Different etiological exposures might occur in different regions, resulting in differences in the prevalence estimates across the different studies [67]. In support of this hypothesis, a lower prevalence was recorded in Australia and Italy, whereas a higher result was identified in China and the United States. Variations in the use of different diagnostic guidelines may have also affected the estimates of prevalence and further contributed to the heterogeneity of results between studies. The discovery of the *JAK2* gene mutations in 2005 and their subsequent inclusion in the diagnostic criteria [68] for MPN, PV and ET but not MF [2,69] may account for some of the differences observed among the studies. A stepwise increase in *TET2* gene mutations in MPN was observed with subsequent versions of the WHO classification and diagnostic criteria among all cases of MPN (12.9% for WHO criteria 2001, 14.5% for WHO criteria 2008 and 20.1% for WHO criteria 2016), PV (13.7% for WHO criteria 2001, 16.9% for WHO criteria 2008 and 21.4% for WHO criteria 2016) and ET (5.3% for WHO criteria 2001, 9.4% for WHO criteria 2008 and 20.3% for WHO criteria 2016) but not in MF (17.5% for WHO criteria 2001, 14.4% for WHO criteria 2008 and 16.5% for WHO criteria 2016).

Another limitation of this meta-analysis is that the prevalence of MPN may be underestimated in some studies. Patients with MPN can be relatively symptom-free for many years so people, with little contact with health services, can remain undiagnosed for long periods [70]. Estimates of the prevalence of *TET2* mutations in MPN may be underestimated, particularly in less-developed countries or among disadvantaged groups in well-developed countries. 

Finally, the included studies largely focused on the allele frequencies of the main driver mutations (*JAK2*, *MPL* and *CALR*) and did not permit any analysis of the allelic frequencies of the *TET2* mutant allele in MPN.

## 5. Conclusions

This meta-analysis provides the most comprehensive currently available estimate of the overall prevalence of *TET2* gene mutations (15.5%) among patients with MPN. However, substantial heterogeneity was evident among the results included in this meta-analysis, likely related to factors such as regional differences in patients included in studies and variations in the diagnostic criteria employed by the studies. This heterogeneity suggests that caution should be employed with using the estimates of prevalence.

## Figures and Tables

**Table 1:** Major characteristics of the included studies.

No	Study ID [References]	Study Design	Country	Type of MPN	Total Number of MPN Patients (Female)	Age (Years) [Mean ± SD/Median (IQR)/Range]	Haemoglobin (g/dL) [Mean ± SD/Median (IQR)/Range]	Leucocyte Count (10^9^/L) [Mean ± SD/Range/Median (IQR)]	Platelet Count (10^9^/L) [Mean ± SD/Range/Median (IQR)]	Total Number of Mutated *ASXL1* (%)	Screening Method for *TET2* Gene Mutations	Diagnostic Criteria
1	Andreasson 2020 [20]	Cross-sectional	Sweden	PV	85 (41)	71.0 (37.0–94.0)	NR	NR	NR	8.2	NGS	2008 WHO
2	Barraco 2017 [21]	Cross-sectional	USA	PV	267 (125)	64.0 (17.0–94.0)	18.0 (14.8–24.3)	11.5 (4.3–59.3)	439.0 (37.0–2747.0)	8.1	NR	2016 WHO
3	Bartels 2019 [22]	Case–control	Germany	MF	104 (53)	NR	NR	NR	NR	9.6	NGS	2016 WHO
4	Brecqueville 2012 [23]	Cross-sectional	France	PV, ET & MF	127 (57)	NR (29.0–97.0)	NR	NR	NR	11.0	SS	2008 WHO
5	Brecqueville 2014 [24]	Cross-sectional	France	MF	68 (NR)	69.0 (30.0–86.0)	11.4 (5.8–17.8)	8.9 (1.3–120.0)	256.0 (5.0–1188.0)	26.5	SS	2008 WHO
6	Carbuccia 2009 [25]	Cross-sectional	France	PV, ET & MF	NR	NR	NR	NR	NR	7.3	SS	NR
7	Cerquozzi 2017 [26]	Cross-sectional	USA	PV	587 (302)	60.0 (17.0–94.0)	NR	NR	476.0 (41.0–2747.0)	10.5	NGS	2016 WHO, ELN
8	Delhommeau 2009 [11]	Cross-sectional	France	PV, ET & MF	203 (41)	NR	NR	NR	NR	NR	SS, SNP array, CGH	2001 WHO
9	Delic 2016 [27]	Cross-sectional	Germany	PV, ET & MF	100 (NR)	69.0 (28.0–87.0)	NR	NR	NR	21.0	NGS	2008 WHO
10	Gill 2018 [28]	Cross-sectional	China	MF	101 (39)	60.0 (26.0–89.0)	10.3 (3.0–18.5)	12.1 (1.5–177.4)	344.0 (19.0–1720.0)	30.7	NGS	2016 WHO, IWG-MRT
11	Guglielmelli 2011 [29]	Cross-sectional	Italy	MF	518 (303)	NR	NR	NR	NR	22.2	HRM	2008 WHO, IWG-MRT
12	Ha 2014 [14]	Cross-sectional	Korea	PV, ET & MF	99 (50)	63.7 ± 13.0	13.7 ± 3.8	16.5 ± 15.4	825.4 ± 490.0	NR	SS, SNP array, CGH	2008 WHO
13	Huang 2020 [30]	Cross-sectional	China	PV, ET & MF	65 (32)	62.0 (NR)	NR	NR	NR	10.8	NGS	2016 WHO
14	Hussein 2010 [31]	Cross-sectional	USA	PV, ET & MF	199 (96)	58.0 (19.0–93.0)	NR	NR	NR	NR	NGS	2001 WHO
15	Kröger 2017 [32]	Cross-sectional	Germany	MF	169 (73)	58.0 (18.0–75.0)	NR	NR	NR	29.0	SS	NR
16	Leibundgut 2020 [33]	Cross-sectional	Switzerland	ET	18 (10)	59.5 (21.0–83.0)	NR	7.8 (3.0–14.6)	788.0 (521.0–1359.0)	11.1	NGS	2016 WHO
17	Magor 2016 [34]	Cross-sectional	Australia	PV, ET & MF	43 (16)	61.0 (24.0–91.0)	NR	NR	NR	9.3	Targeted exon resequencing	2008 WHO
18	Martínez-Avilés 2012 [35]	Cross-sectional	Spain	PV, ET & MF	62 (43)	NR	NR	NR	NR	4.8	HRM, SS	2008 WHO
19	Nielsen 2017 [36]	Case–control	Denmark	MF	16 (3)	66.0 (52.0–80.0)	10.3 (7.9–13.4)	5.9 (2.3–64.4)	155.5 (56.0–357.0)	50.0	PCR-DGGE	NR
20	Nischal 2013 [37]	Cross-sectional	USA	PV, ET & MF	25 (14)	68.0 (54.0–72.0)	NR	NR	NR	24.0	SS	NR
21	O’Sullivan 2019 [38]	Cross-sectional	UK	ET	NR	NR	NR	NR	NR	NR	NGS	NR
22	Pardanani 2010 [39]	Cross-sectional	USA	PV, ET & MF	78 (34)	64.0 (22.0–95.0)	NR	NR	NR	NR	NGS	2008 WHO
23	Patel 2015 [40]	Cross-sectional	USA	MF	95 (44)	66.0 (40.0–84.0)	10.7 (7.2–16.9)	25.0 (2.5–159.0)	339.0 (13.0–969.0)	21.1	NGS	IWG-MRT
24	Patriarca 2013 [41]	Cross-sectional	Italy	PV, ET & MF	97 (44)	NR	NR	NR	NR	NR	NGS	2008 WHO
25	Saint-Martin 2009 [42]	Cross-sectional	France	PV, ET & MF	NR	NR	NR	NR	NR	NR	SS	2008 WHO
26	Schlenk 2016 [43]	Cross-sectional	Germany	MF	96 (33)	NR	NR	NR	NR	30.2	SS	2008 WHO, IWG-MRT
27	Schnittger 2012 [44]	Cross-sectional	Germany	ET & MF	NR	NR	NR	NR	NR	NR	SS, HRM	NR
28	Segura-Díaz 2020 [45]	Cross-sectional	Spain	PV, ET & MF	68 (40)	68.0 (43.0–90.0)	NR	NR	NR	8.8	NGS	2016 WHO
29	Song 2017 [46]	Cross-sectional	USA	PV, ET & MF	135 (64)	NR	NR	NR	NR	21.2	NGS	2008 WHO
30	Tefferi 2009 [47]	Cross-sectional	USA	PV, ET & MF	227 (111)	NR	NR	NR	NR	NR	NGS	2001 WHO
31	Tefferi 2010 [48]	Cross-sectional	USA	PV, ET & MF	908 (487)	NR	NR	NR	NR	NR	NGS	2008 WHO, IWG-MRT
32	Tefferi 2016 [49]	Cross-sectional	USA	MF	182 (64)	63.0 (22.0–87.0)	10.1 (5.8–16.0)	10.5 (1.9–219.0)	224.0 (11.0–1493.0)	35.7	NGS	2008 WHO
33	Tefferi 2016a [50]	Cross-sectional	USA	PV & ET	316 (177)	NR	NR	NR	NR	11.4	NGS	2008 WHO
34	Verger 2014 [51]	Cross-sectional	France	PV, ET & MF	27 (NR)	NR	NR	NR	NR	NR	SS	NR
35	Zhang 2015 [52]	Cross-sectional	China	MF	36 (15)	65.0 (46.0–93.0)	10.9 (3.0–16.0)	22.3 (1.4–54.5)	215.0 (3.0–1157.0)	11.1	WGS	2008 WHO

aCGH: array-comparative genomic hybridisation; ASXL1: Additional sex combs-like 1; CGH: comparative genomic hybridisation; ELN: European Leukemia Net; ET: essential thrombocythaemia; HRM: high-resolution melting analysis; IQR: interquartile range; IWG-MRT: International Working Group for Myelofibrosis Research and Treatment; MF: myelofibrosis; MPN: myeloproliferative neoplasms; SS: Sanger sequencing; NGS: next-generation sequencing; NR: not reported; PCR-DGGE: polymerase chain reaction-denaturing gradient gel electrophoresis; PV: polycythaemia vera; SD: standard deviation; SNP: single nucleotide polymorphism; TET2: Ten–eleven translocation 2; UK: United Kingdom; USA: United States of America; WGS: whole-genome sequencing; WHO: World Health Organization.

**Table 2:** The pooled prevalence of *TET2* gene mutations in different subgroups of MPN.

Subgroups	Prevalence [95% CIs] (%)	Number of Studies Analysed	Total Number of Patients	Heterogeneity		Publication Bias, Egger’s Test (*p*-Value)
				*I* ^2^	*p*-Value	
**Overall myeloproliferative neoplasms**						
**Europe**	13.0 [8.8–17.2]	19	2010	92%	<0.0001	0.004
**North America**	17.4 [14.0–20.9]	11	1976	74%	<0.0001	0.0005
**Asia**	20.8 [10.5–31.1]	4	291	80%	0.001	NA
**Australia**	7.0 [0.0–14.6]	1	43	NA	NA	NA
**China**	23.9 [9.6–38.1]	3	200	82%	0.003	NA
**France**	13.6 [10.6–16.7]	5	480	0%	0.67	NA
**Germany**	14.2 [9.2–19.1]	5	510	61%	0.03	NA
**Italy**	1.9 [0.0–5.7]	2	607	71%	0.06	NA
**Spain**	10.7 [0.0–23.2]	2	130	82%	0.01	NA
**USA**	17.4 [14.0–20.9]	11	1976	74%	<0.0001	0.0005
**WHO criteria reported**	15.7 [11.8–19.7]	27	3782	95%	<0.0001	0.0002
**WHO criteria not reported**	13.1 [8.9–17.3]	8	538	47%	0.06	0.005
**WHO 2001 criteria**	12.9 [10.2–15.5]	3	613	0%	0.93	NA
**WHO 2008 criteria**	14.5 [9.7–19.3]	17	2594	95%	<0.0001	0.0004
**WHO 2016 criteria**	20.1 [14.7–25.4]	7	575	61%	0.01	0.40
**NGS method**	17.2 [14.0–20.4]	18	2604	80%	<0.0001	0.0007
**SS method**	12.7 [9.6–15.9]	11	965	52%	0.02	0.001
**HRM method**	7.7 [0.0–16.6]	3	621	88%	0.0002	NA
**Polycythaemia vera**						
**Europe**	14.6 [8.0–21.1]	10	343	63%	0.01	0.58
**North America**	18.2 [14.2–22.5]	9	839	57%	0.01	NA
**Asia**	29.6 [14.1–45.2]	2	39	17%	0.27	NA
**Australia**	0.0 [0.0–15.0]	1	8	NA	NA	NA
**France**	12.5 [7.6–17.5]	4	172	0%	0.90	NA
**Spain**	12.7 [0.0–37.2]	2	21	61%	0.28	NA
**USA**	18.2 [14.0–22.5]	9	839	57%	0.01	NA
**WHO 2001 criteria**	13.7 [9.6–17.9]	3	260	0%	0.40	NA
**WHO 2008 criteria**	16.9 [11.3–22.6]	12	685	69%	0.0009	0.77
**WHO 2016 criteria**	21.4 [15.6–27.3]	4	256	16%	0.31	NA
**NGS method**	19.8 [15.1–24.6]	12	922	67%	0.0005	0.009
**SS method**	13.0 [8.4–17.7]	7	203	0%	0.71	NA
**HRM method**	0.0 [0.0–22.1]	1	5	NA	NA	NA
**Essential thrombocythaemia**						
**Europe**	8.8 [5.7–12.0]	12	531	39%	0.08	0.002
**North America**	8.7 [3.8–13.6]	7	507	69%	0.003	NA
**Asia**	25.1 [0.0–56.9]	2	100	93%	0.0002	NA
**Australia**	6.2 [0.0–18.1]	1	16	NA	NA	NA
**France**	9.7 [5.3–14.2]	4	166	0%	0.44	NA
**Spain**	12.1 [0.0–21.2]	2	46	74%	0.04	NA
**USA**	8.7 [3.8–13.6]	7	507	69%	0.003	NA
**WHO 2001 criteria**	5.3 [1.1–9.6]	3	180	44%	0.16	NA
**WHO 2008 criteria**	9.4 [6.1–12.6]	11	700	49%	0.03	0.06
**WHO 2016 criteria**	20.3 [0.0–43.7]	3	81	89%	<0.0001	0.41
**NGS method**	10.2 [6.1–14.4]	12	787	75%	<0.0001	0.003
**SS method**	10.4 [6.2–14.6]	8	316	31%	0.18	NA
**HRM method**	14.9 [0.0–35.2]	2	82	83%	0.01	NA
**Myelofibrosis**						
**Europe**	13.7 [7.9–19.5]	15	1127	85%	<0.0001	0.008
**North America**	16.8 [12.3–23.7]	9	640	52%	0.09	NA
**Asia**	17.4 [11.4–23.5]	4	152	0%	0.82	NA
**Australia**	10.5 [0.0–24.3]	1	19	NA	NA	NA
**China**	17.4 [11.2–23.6]	3	141	0%	0.63	NA
**France**	17.6 [9.9–25.3]	5	142	20%	0.51	NA
**Germany**	11.0 [8.0–14.0]	5	410	0%	0.61	NA
**Italy**	0.4 [0.0–0.9]	2	527	0%	0.50	NA
**Spain**	14.0 [0.0–39.4]	2	32	70%	0.21	NA
**USA**	17.7 [13.8–21.6]	8	631	35%	0.15	NA
**WHO 2001 criteria**	17.5 [11.9–23.3]	3	173	0%	0.52	NA
**WHO 2008 criteria**	14.4 [8.1–20.7]	15	1210	90%	<0.0001	0.04
**WHO 2016 criteria**	16.5 [11.8–21.2]	4	238	0%	0.39	0.20
**NGS method**	16.5 [13.2–19.8]	13	896	38%	0.17	0.053
**SS method**	13.3 [9.1–17.5]	11	446	24%	0.35	0.01
**HRM method**	5.0 [0.0–18.2]	3	534	50%	0.10	NA
**Different types of myelofibrosis**						
**PMF**	16.7 [13.6–19.8]	20	853	24%	0.41	0.06
**SMF**	14.8 [9.3–20.2]	9	158	0%	0.95	NA

CIs: confidence intervals; HRM: high-resolution melting analysis; NA: not applicable; NGS: next-generation sequencing; PMF: primary myelofibrosis; SMF: secondary myelofibrosis; SS: Sanger sequencing; WHO: World Health Organization.

**Table 3:** Sensitivity analyses.

Strategies of Sensitivity Analyses	Prevalence [95% CIs] (%)	Difference of Pooled Prevalence Compared to the Main Result	Number of Studies Analysed	Total Number of Subjects	Heterogeneity	
					*I* ^2^	*p*-Value
**Myeloproliferative neoplasms (overall)**						
Excluding small studies	13.6 [8.8–18.4]	1.9% lower	15	3117	96%	<0.0001
Excluding low- and moderate-quality studies	15.4 [11.3–19.6]	0.1% lower	24	3485	95%	<0.0001
Excluding outlier studies	13.9 [12.0–15.9]	1.6% lower	31	3633	63%	<0.0001
**Polycythaemia vera**						
Excluding small studies	15.6 [11.0–20.3]	1.2% lower	4	614	59%	0.06
Excluding low- and moderate-quality studies	18.6 [14.4–22.7]	1.8% higher	13	946	59%	0.003
Excluding outlier studies	15.4 [12.0–18.7]	1.4% lower	19	1161	54%	0.01
**Essential thrombocythaemia**						
Excluding small studies	11.3 [5.9–16.8]	1.5% higher	3	470	72%	0.02
Excluding low- and moderate-quality studies	11.1 [7.1–15.0]	1.3% higher	12	839	72%	<0.0001
Excluding outlier studies	8.4 [6.0–10.8]	4.4% lower	19	1096	48%	0.01
**Myelofibrosis**						
Excluding small studies	11.7 [4.0–19.5]	4.0% lower	6	1191	95%	<0.0001
Excluding low- and moderate-quality studies	14.0 [8.9–19.1]	1.7% lower	18	1700	91%	<0.0001
Excluding outlier studies	14.5 [12.4–16.7]	1.2% lower	25	1377	18%	0.46

CIs: Confidence intervals.

## Data Availability

The data presented in this study are available within the article and supplementary materials.

## Supplementary Materials

The following are available online at https://www.mdpi.com/article/10.3390/cancers13123078/s1, Figure S1: prevalence of *TET2* gene mutations based on continents, Figure S2: Prevalence of *TET2* gene mutations based on countries, Figure S3: Prevalence of *TET2* gene mutations based on the WHO classification and diagnostic criteria of MPN, Figure S4: Prevalence of *TET2* gene mutations based on different methods used to detect *TET2* gene mutations in MPN, Figure S5: Subgroup analysis. Prevalence of *TET2* gene mutations in patients with (A) PMF and (B) SMF, Figure S6: Funnel plots estimating the prevalence of *TET2* gene mutations in patients with (A) polycythaemia vera, (B) essential thrombocythaemia and (C) myelofibrosis, Figure S7: Sensitivity analyses, Table S1: Search strategies, Table S2: Quality assessment of the included cross-sectional studies, Table S3: Quality assessment of the included case–control studies.
