# Supplementary material for: The Prevalence of TET2 Gene Mutations in Patients with BCR-ABL-Negative Myeloproliferative Neoplasms (MPN): A Systematic Review and Meta-Analysis
Source: Cancers (Basel). 2021 Jun 20;13(12):3078. doi: 10.3390/cancers13123078 (PMC8235080; doi:10.3390/cancers13123078)
Supplement: Supplementary file 1 [file cancers-13-03078-s001.zip › cancers-1207393-supplementary.pdf]

Supplementary materials

Table S1. Search strategies.

Databases	Search strategies
**PubMed**	(Ten-Eleven Translocation 2[Title/Abstract] OR TET2[Title/Abstract]) AND (myeloproliferative[Title/Abstract] OR vera[Title/Abstract] OR thrombocythemia[Title/Abstract] OR thrombocytosis [Title/Abstract] OR myelofibrosis[Title/Abstract])
**ScienceDirect**	("Ten-Eleven Translocation 2" OR TET2) AND (myeloproliferative OR vera OR thrombocythemia OR thrombocytosis OR myelofibrosis)
**Embase**	('Ten-Eleven Translocation 2':ti OR TET2:ti) AND (myeloproliferative:ti OR vera:ti OR thrombocythemia:ti OR thrombocytosis:ti OR myelofibrosis:ti)
**Scopus**	TITLE-ABS("Ten-Eleven Translocation 2" OR TET2) AND TITLE-ABS(myeloproliferative OR vera OR thrombocythemia OR thrombocytosis OR myelofibrosis)
**Web of Science**	TI=("Ten-Eleven Translocation 2" OR TET2) AND TI=(myeloproliferative OR vera OR thrombocythemia OR thrombocytosis OR myelofibrosis)
**Google Scholar**	allintitle: myeloproliferative TET2<br>allintitle: myeloproliferative Ten-Eleven Translocation 2<br>allintitle: MPN TET2<br>allintitle: vera TET2<br>allintitle: thrombocythemia TET2<br>allintitle: thrombocytosis TET2<br>allintitle:myelofibrosis TET2

Table S2. Quality assessment of the included cross-sectional studies.

No.	Study ID	Questions assessing included cross-sectional studies								Yes
		1	2	3	4	5	6	7	8	(%)
1	Andreasson 2020 [1]	Y	Y	Y	Y	Y	Y	Y	Y	100.0
2	Barraco 2017 [2]	Y	Y	N	Y	Y	Y	N	Y	75.0
3	Brecqueville 2012 [3]	Y	Y	Y	Y	Y	Y	Y	Y	100.0
4	Brecqueville 2014 [4]	Y	Y	Y	Y	Y	Y	Y	Y	100.0
5	Carbuccia 2009 [5]	N	N	Y	Y	N	N	Y	N	37.5
6	Cerquozzi 2017 [6]	Y	Y	Y	Y	Y	Y	Y	Y	100.0
7	Delhommeau 2009 [7]	Y	Y	Y	Y	N	N	Y	N	62.5
8	Delic 2016 [8]	Y	Y	Y	Y	Y	Y	Y	Y	100.0
9	Gill 2018 [9]	Y	Y	Y	Y	Y	Y	Y	Y	100.0
10	Guglielmelli 2011 [10]	Y	Y	N	Y	Y	Y	Y	Y	87.5
11	Ha 2014 [11]	Y	Y	Y	Y	Y	Y	Y	Y	100.0
12	Huang 2020 [12]	Y	Y	Y	Y	Y	Y	Y	Y	100.0
13	Hussein 2010 [13]	Y	N	Y	Y	Y	N	Y	N	62.5
14	Kröger 2017 [14]	N	Y	Y	N	Y	Y	Y	Y	75.0
15	Leibundgut 2020 [15]	Y	Y	Y	Y	Y	Y	Y	Y	100.0
16	Magor 2016 [16]	Y	Y	Y	Y	N	N	Y	N	62.5
17	Martínez-Avilés 2012 [17]	Y	Y	Y	Y	N	N	Y	N	62.5

18	Nischal 2013 [18]	N	N	Y	Y	N	N	Y	N	37.5
19	O'Sullivan 2019 [19]	N	N	Y	Y	Y	Y	Y	Y	75.0
20	Pardanani 2010 [20]	Y	Y	Y	Y	N	N	Y	N	62.5
21	Patel 2015 [21]	N	N	Y	Y	Y	Y	Y	Y	75.0
22	Patriarca 2013 [22]	Y	Y	Y	Y	Y	Y	Y	Y	100.0
23	Saint-Martin 2009 [23]	Y	N	Y	Y	Y	Y	Y	Y	87.5
24	Schlenk 2016 [24]	Y	Y	N	Y	Y	Y	Y	Y	87.5
25	Schnittger 2012 [25]	N	N	Y	N	N	N	Y	N	25.0
26	Segura-Díaz 2020 [26]	Y	Y	Y	Y	Y	Y	Y	Y	100.0
27	Song 2017 [27]	Y	N	Y	Y	N	N	Y	N	50.0
28	Tefferi 2009 [28]	Y	Y	Y	Y	Y	Y	Y	Y	100.0
29	Tefferi 2010 [29]	Y	Y	Y	Y	Y	Y	Y	Y	100.0
30	Tefferi 2016 [30]	Y	Y	Y	Y	Y	Y	Y	Y	100.0
31	Tefferi 2016a [31]	Y	Y	Y	Y	Y	Y	Y	Y	100.0
32	Verger 2014 [32]	N	N	Y	N	N	N	Y	N	25.0
33	Zhang 2015 [33]	Y	Y	Y	Y	Y	Y	Y	Y	100.0

1. Were the criteria for inclusion in the sample clearly defined? 2. Were the study subjects and the setting described in detail? 3. Was the exposure measured in a valid and reliable way? 4. Were objective, standard criteria used for measurement of the condition? 5. Were confounding factors identified? 6. Were strategies to deal with confounding factors stated? 7. Were the outcomes measured in a valid and reliable way? 8. Was appropriate statistical analysis used? Y=Yes; N=No; U=Unclear

Table S3. Quality assessment of the included case-control studies.

**No.**	**Study ID**	**Questions assessing included case-control studies**										**Yes**
		**1**	**2**	**3**	**4**	**5**	**6**	**7**	**8**	**9**	**10**	**(%)**
1	Bartels 2019 [34]	Y	Y	Y	Y	Y	Y	Y	Y	Y	N	90.0
2	Nielsen 2017 [35]	N	N	N	Y	Y	N	N	Y	Y	N	40.0

1. Were the groups comparable other than the presence of disease in caseNs or the absence of disease in controls? 2. Were cases and controls matched appropriately? 3. Were the same criteria used for identification of cases and controls? 4. Was exposure measured in a standard, valid and reliable way? 5. Was exposure measured in the same way for cases and controls? 6. Were confounding factors identified? 7. Were strategies to deal with confounding factors stated? 8. Were outcomes assessed in a standard, valid and reliable way for cases and controls? 9. Was the exposure period of interest long enough to be meaningful? 10. Was appropriate statistical analysis used? Y=Yes; N=No; U=Unclear

A

Study ID	Cases	Total	Prevalence	95% C.I.
**Overall myeloproliferative neoplasms (Europe)**				
Andreasson 2020	26	85	30.6	[20.8; 40.4]
Bartels 2019	14	104	13.5	[6.9; 20.0]
Brecqueville 2012	15	127	11.8	[6.2; 17.4]
Brecqueville 2014	11	68	16.2	[7.4; 24.9]
Carbuccia 2009	4	55	7.3	[0.4; 14.1]
Delhommeau 2009	26	197	13.2	[8.5; 17.9]
Delic 2016	20	100	20.0	[12.2; 27.8]
Guglielmelli 2011	2	518	0.4	[0.0; 0.9]
Kröger 2017	16	169	9.5	[5.1; 13.9]
Leibundgut 2020	3	18	16.7	[0.0; 33.9]
Martínez-Avilés 2012	3	62	4.8	[0.0; 10.2]
Nielsen 2017	3	15	20.0	[0.0; 40.2]
O'Sullivan 2019	12	110	10.9	[5.1; 16.7]
Patriarca 2013	4	89	4.5	[0.2; 8.8]
Saint-Martin 2009	12	61	19.7	[9.7; 29.6]
Schlenk 2016	10	96	10.4	[4.3; 16.5]
Schnittger 2012	11	41	26.8	[13.3; 40.4]
Segura-Díaz 2020	12	68	17.6	[8.6; 26.7]
Verger 2014	3	27	11.1	[0.0; 23.0]
**Random effects model**		**2010**	**13.0**	**[8.8; 17.2]**

Heterogeneity: $I^2 = 92\%$, $\tau^2 = 0.0069$, $\chi^2_{18} = 215.90$ ($p < 0.01$)

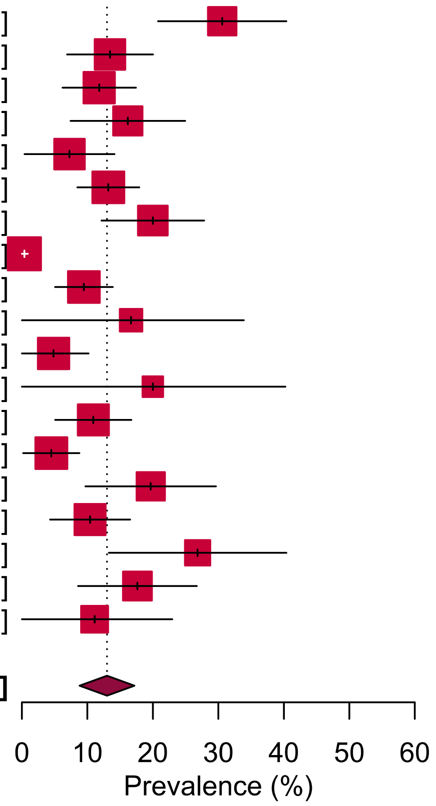

B

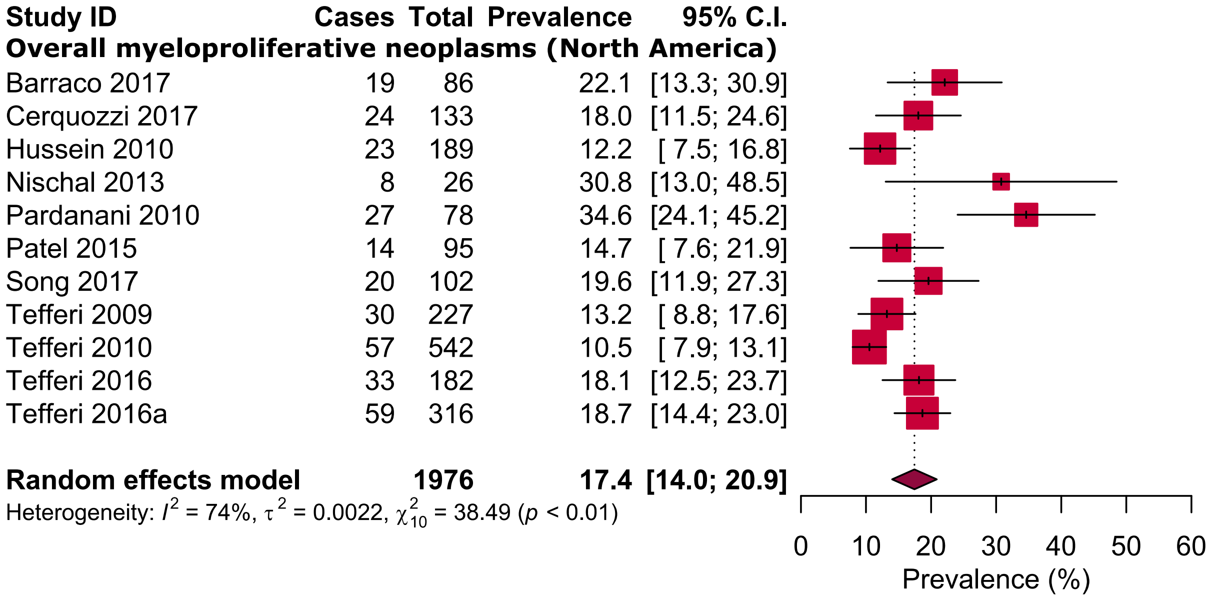
Study ID Cases Total Prevalence 95% C.I.
Overall myeloproliferative neoplasms (North America)
Barraco 2017 19 86 22.1 [13.3; 30.9]
Cerquozzi 2017 24 133 18.0 [11.5; 24.6]
Hussein 2010 23 189 12.2 [7.5; 16.8]
Nischal 2013 8 26 30.8 [13.0; 48.5]
Pardanani 2010 27 78 34.6 [24.1; 45.2]
Patel 2015 14 95 14.7 [7.6; 21.9]
Song 2017 20 102 19.6 [11.9; 27.3]
Tefferi 2009 30 227 13.2 [8.8; 17.6]
Tefferi 2010 57 542 10.5 [7.9; 13.1]
Tefferi 2016 33 182 18.1 [12.5; 23.7]
Tefferi 2016a 59 316 18.7 [14.4; 23.0]
Random effects model 1976 17.4 [14.0; 20.9]
Heterogeneity: I^2 = 74%, τ^2 = 0.0022, χ^2_{10} = 38.49 (p < 0.01)
0 10 20 30 40 50 60
Prevalence (%)

C

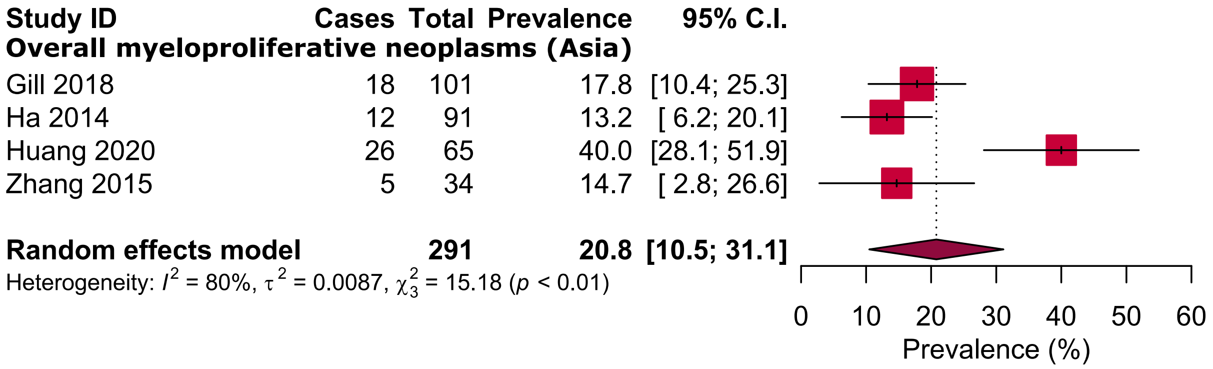
Study ID Cases Total Prevalence 95% C.I.
Overall myeloproliferative neoplasms (Asia)
Gill 2018 18 101 17.8 [10.4; 25.3]
Ha 2014 12 91 13.2 [6.2; 20.1]
Huang 2020 26 65 40.0 [28.1; 51.9]
Zhang 2015 5 34 14.7 [2.8; 26.6]
Random effects model 291 20.8 [10.5; 31.1]
Heterogeneity: I^2 = 80%, τ^2 = 0.0087, χ^2_3 = 15.18 (p < 0.01)
0 10 20 30 40 50 60
Prevalence (%)

D

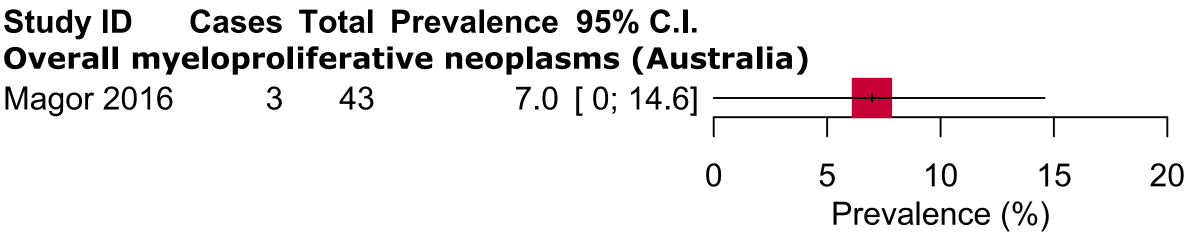
Study ID Cases Total Prevalence 95% C.I.
Overall myeloproliferative neoplasms (Australia)
Magor 2016 3 43 7.0 [0; 14.6]
0 5 10 15 20
Prevalence (%)

E

Study ID	Cases	Total	Prevalence	95% C.I.
**Essential thrombocythemia (Europe)**				
Brecqueville 2012	4	53	7.5	[0.4; 14.7]
Carbuccia 2009	2	35	5.7	[0.0; 13.4]
Delhommeau 2009	8	80	10.0	[3.4; 16.6]
Delic 2016	7	40	17.5	[5.7; 29.3]
Leibundgut 2020	3	18	16.7	[0.0; 33.9]
Martínez-Avilés 2012	3	52	5.8	[0.0; 12.1]
O'Sullivan 2019	12	110	10.9	[5.1; 16.7]
Patriarca 2013	2	55	3.6	[0.0; 8.6]
Saint-Martin 2009	5	21	23.8	[5.6; 42.0]
Schnittger 2012	8	30	26.7	[10.8; 42.5]
Segura-Díaz 2020	1	25	4.0	[0.0; 11.7]
Verger 2014	1	12	8.3	[0.0; 24.0]
**Random effects model**		**531**	**8.8**	**[5.7; 12.0]**

Heterogeneity: $I^2 = 39\%$, $\tau^2 = 0.0011$, $\chi^2_{11} = 17.89$ ($p = 0.08$)

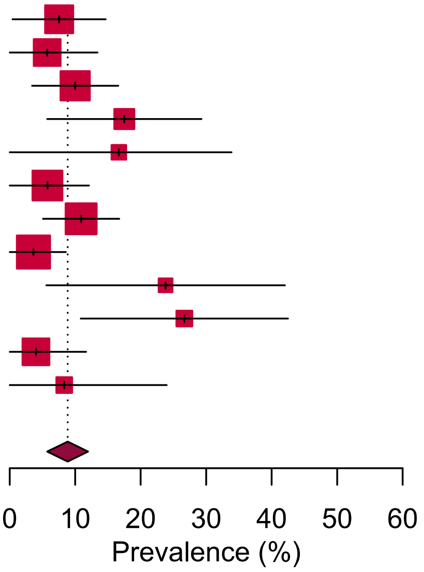

F

Study ID	Cases	Total	Prevalence	95% C.I.
**Essential thrombocythemia (North America)**				
Hussein 2010	1	43	2.3	[0.0; 6.8]
Nischal 2013	2	6	33.3	[0.0; 71.1]
Pardanani 2010	3	14	21.4	[0.0; 42.9]
Song 2017	2	27	7.4	[0.0; 17.3]
Tefferi 2009	3	57	5.3	[0.0; 11.1]
Tefferi 2010	13	177	7.3	[3.5; 11.2]
Tefferi 2016a	30	183	16.4	[11.0; 21.8]
**Random effects model**		**507**	**8.7**	**[3.8; 13.6]**

Heterogeneity: $I^2 = 69\%$, $\tau^2 = 0.0024$, $\chi^2_{6} = 19.61$ ($p < 0.01$)

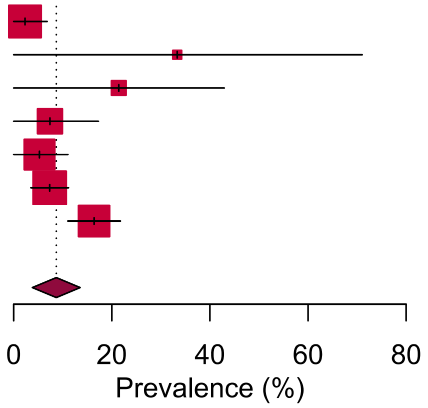

G

Study ID	Cases	Total	Prevalence	95% C.I.
**Essential thrombocythemia (Asia)**				
Ha 2014	6	62	9.7	[2.3; 17.0]
Huang 2020	16	38	42.1	[26.4; 57.8]
**Random effects model**		**100**	**25.1**	**[0.0; 56.9]**

Heterogeneity: $I^2 = 93\%$, $\tau^2 = 0.0487$, $\chi^2_{1} = 13.44$ ($p < 0.01$)

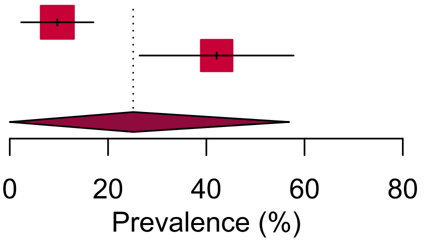

H

Study ID	Cases	Total	Prevalence	95% C.I.
**Essential thrombocythemia (Australia)**				
Magor 2016	1	16	6.2	[0; 18.1]

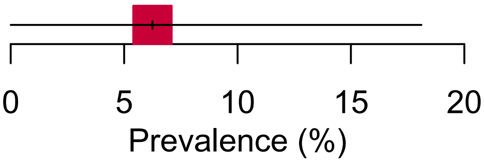

I

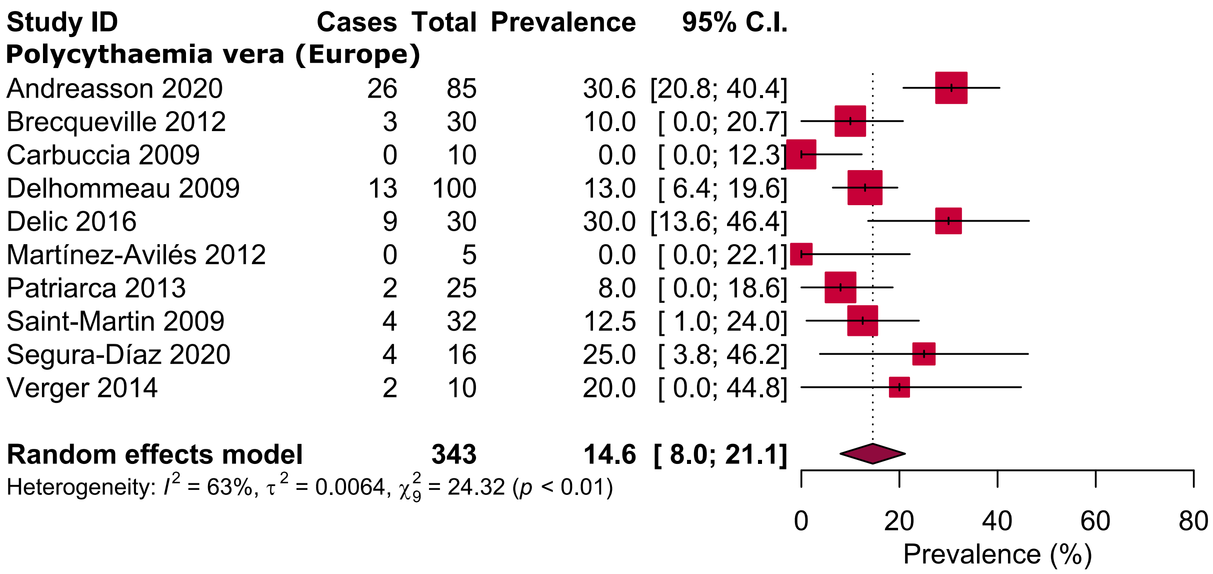
Study ID Cases Total Prevalence 95% C.I.
Polycythaemia vera (Europe)
Andreasson 2020 26 85 30.6 [20.8; 40.4]
Brecqueville 2012 3 30 10.0 [0.0; 20.7]
Carbuccia 2009 0 10 0.0 [0.0; 12.3]
Delhommeau 2009 13 100 13.0 [6.4; 19.6]
Delic 2016 9 30 30.0 [13.6; 46.4]
Martínez-Avilés 2012 0 5 0.0 [0.0; 22.1]
Patriarca 2013 2 25 8.0 [0.0; 18.6]
Saint-Martin 2009 4 32 12.5 [1.0; 24.0]
Segura-Díaz 2020 4 16 25.0 [3.8; 46.2]
Verger 2014 2 10 20.0 [0.0; 44.8]
Random effects model 343 14.6 [8.0; 21.1]
Heterogeneity: I^2 = 63%, τ^2 = 0.0064, χ^2_9 = 24.32 (p < 0.01)
0 20 40 60 80
Prevalence (%)

J

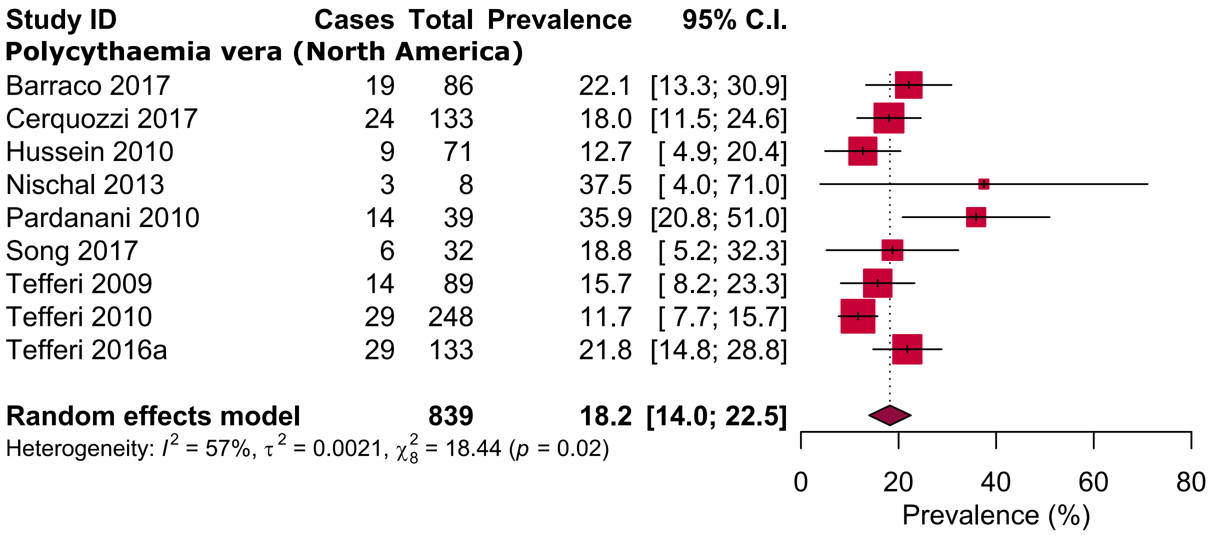
Study ID Cases Total Prevalence 95% C.I.
Polycythaemia vera (North America)
Barraco 2017 19 86 22.1 [13.3; 30.9]
Cerquozzi 2017 24 133 18.0 [11.5; 24.6]
Hussein 2010 9 71 12.7 [4.9; 20.4]
Nischal 2013 3 8 37.5 [4.0; 71.0]
Pardanani 2010 14 39 35.9 [20.8; 51.0]
Song 2017 6 32 18.8 [5.2; 32.3]
Tefferi 2009 14 89 15.7 [8.2; 23.3]
Tefferi 2010 29 248 11.7 [7.7; 15.7]
Tefferi 2016a 29 133 21.8 [14.8; 28.8]
Random effects model 839 18.2 [14.0; 22.5]
Heterogeneity: I^2 = 57%, τ^2 = 0.0021, χ^2_8 = 18.44 (p = 0.02)
0 20 40 60 80
Prevalence (%)

K

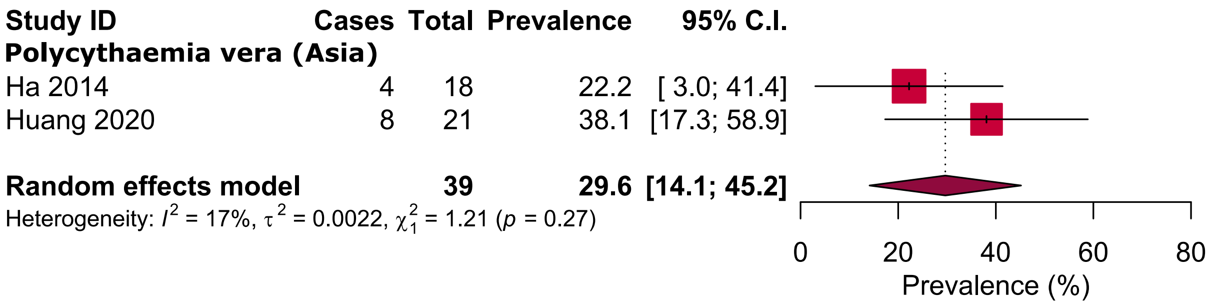
Study ID Cases Total Prevalence 95% C.I.
Polycythaemia vera (Asia)
Ha 2014 4 18 22.2 [3.0; 41.4]
Huang 2020 8 21 38.1 [17.3; 58.9]
Random effects model 39 29.6 [14.1; 45.2]
Heterogeneity: I^2 = 17%, τ^2 = 0.0022, χ^2_1 = 1.21 (p = 0.27)
0 20 40 60 80
Prevalence (%)

L

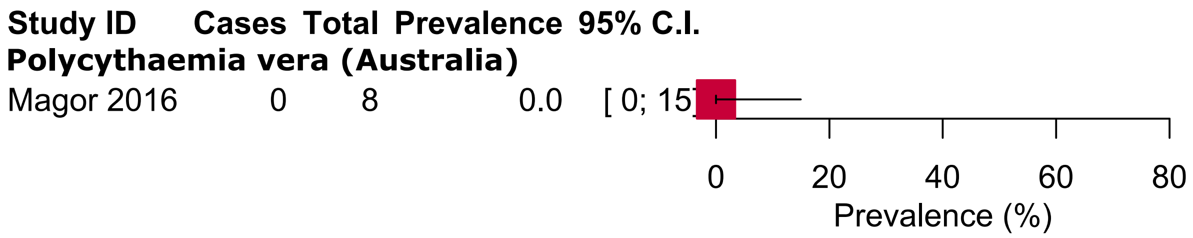
Study ID Cases Total Prevalence 95% C.I.
Polycythaemia vera (Australia)
Magor 2016 0 8 0.0 [0; 15]
0 20 40 60 80
Prevalence (%)

M

Study ID	Cases	Total	Prevalence	95% C.I.
**Myelofibrosis (Europe)**				
Bartels 2019	14	104	13.5	[6.9; 20.0]
Brecqueville 2012	8	44	18.2	[6.8; 29.6]
Brecqueville 2014	11	68	16.2	[7.4; 24.9]
Carbuccia 2009	2	10	20.0	[0.0; 44.8]
Delhommeau 2009	5	17	29.4	[7.8; 51.1]
Delic 2016	4	30	13.3	[1.2; 25.5]
Guglielmelli 2011	2	518	0.4	[0.0; 0.9]
Kröger 2017	16	169	9.5	[5.1; 13.9]
Martínez-Avilés 2012	0	5	0.0	[0.0; 22.1]
Nielsen 2017	3	15	20.0	[0.0; 40.2]
Saint-Martin 2009	3	8	37.5	[4.0; 71.0]
Schlenk 2016	10	96	10.4	[4.3; 16.5]
Schnittger 2012	3	11	27.3	[1.0; 53.6]
Segura-Díaz 2020	7	27	25.9	[9.4; 42.5]
Verger 2014	0	5	0.0	[0.0; 22.1]
**Random effects model**		**1127**	**13.7**	**[7.9; 19.5]**

Heterogeneity: $I^2 = 85\%$, $\tau^2 = 0.0075$, $\chi^2_{14} = 95.58$ ($p < 0.01$)

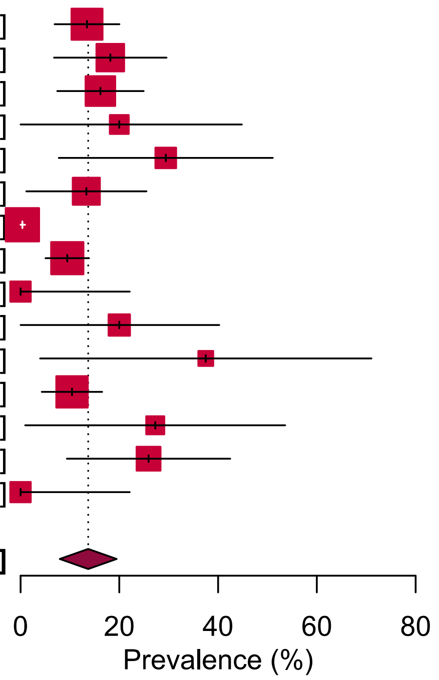

N

Study ID	Cases	Total	Prevalence	95% C.I.
**Myelofibrosis (North America)**				
Hussein 2010	13	75	17.3	[8.8; 25.9]
Nischal 2013	3	12	25.0	[0.5; 49.5]
Pardanani 2010	10	25	40.0	[20.8; 59.2]
Patel 2015	14	95	14.7	[7.6; 21.9]
Patriarca 2013	0	9	0.0	[0.0; 13.5]
Song 2017	12	44	27.3	[14.1; 40.4]
Tefferi 2009	13	81	16.0	[8.1; 24.0]
Tefferi 2010	15	117	12.8	[6.8; 18.9]
Tefferi 2016	33	182	18.1	[12.5; 23.7]
**Random effects model**		**640**	**16.8**	**[12.3; 21.4]**

Heterogeneity: $I^2 = 52\%$, $\tau^2 = 0.0022$, $\chi^2_8 = 16.52$ ($p = 0.04$)

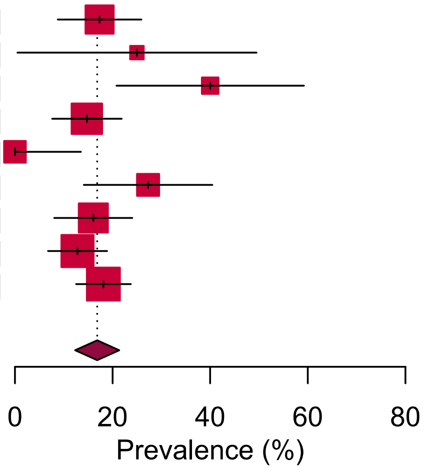

O

Study ID	Cases	Total	Prevalence	95% C.I.
**Myelofibrosis (Asia)**				
Gill 2018	18	101	17.8	[10.4; 25.3]
Ha 2014	2	11	18.2	[0.0; 41.0]
Huang 2020	2	6	33.3	[0.0; 71.1]
Zhang 2015	5	34	14.7	[2.8; 26.6]
**Random effects model**		**152**	**17.4**	**[11.4; 23.5]**

Heterogeneity: $I^2 = 0\%$, $\tau^2 = 0$, $\chi^2_3 = 0.90$ ($p = 0.83$)

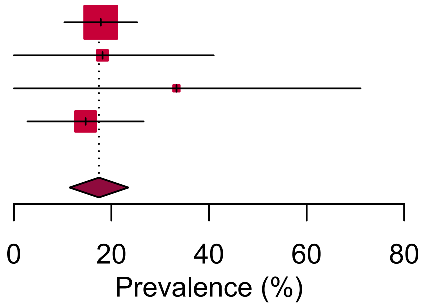

P

Study ID	Cases	Total	Prevalence	95% C.I.
**Myelofibrosis (Australia)**				
Magor 2016	2	19	10.5	[0; 24.3]

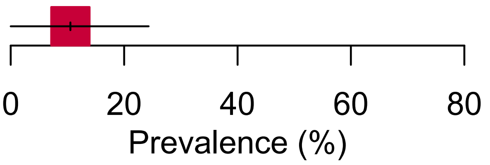

Figure S1. Prevalence of *TET2* gene mutations based on continents.

A

Study ID	Cases	Total	Prevalence	95% C.I.
**Overall myeloproliferative neoplasms (China)**				
Gill 2018	18	101	17.8	[10.4; 25.3]
Huang 2020	26	65	40.0	[28.1; 51.9]
Zhang 2015	5	34	14.7	[2.8; 26.6]
**Random effects model**		**200**	**23.9**	**[9.6; 38.1]**

Heterogeneity: I^2 = 82%, τ^2 = 0.0130, χ^2_2 = 11.42 (p < 0.01)

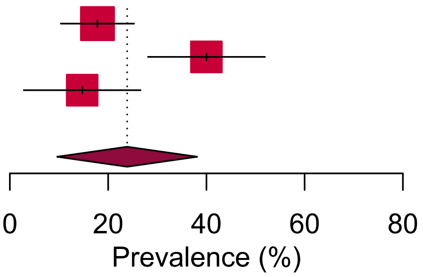

B

Study ID	Cases	Total	Prevalence	95% C.I.
**Overall myeloproliferative neoplasms (France)**				
Brecqueville 2012	15	127	11.8	[6.2; 17.4]
Brecqueville 2014	11	68	16.2	[7.4; 24.9]
Delhommeau 2009	26	197	13.2	[8.5; 17.9]
Saint-Martin 2009	12	61	19.7	[9.7; 29.6]
Verger 2014	3	27	11.1	[0.0; 23.0]
**Random effects model**		**480**	**13.6**	**[10.6; 16.7]**

Heterogeneity: I^2 = 0%, τ^2 = 0, χ^2_4 = 2.34 (p = 0.67)

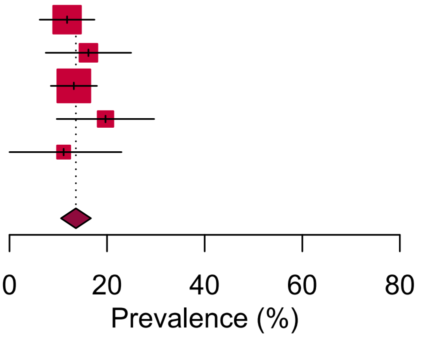

C

Study ID	Cases	Total	Prevalence	95% C.I.
**Overall myeloproliferative neoplasms (Germany)**				
Bartels 2019	14	104	13.5	[6.9; 20.0]
Delic 2016	20	100	20.0	[12.2; 27.8]
Kröger 2017	16	169	9.5	[5.1; 13.9]
Schlenk 2016	10	96	10.4	[4.3; 16.5]
Schnittger 2012	11	41	26.8	[13.3; 40.4]
**Random effects model**		**510**	**14.2**	**[9.2; 19.1]**

Heterogeneity: I^2 = 61%, τ^2 = 0.0018, χ^2_4 = 10.14 (p = 0.04)

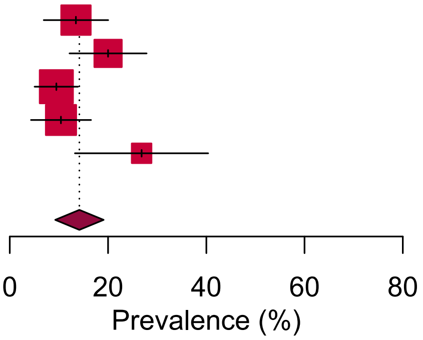

D

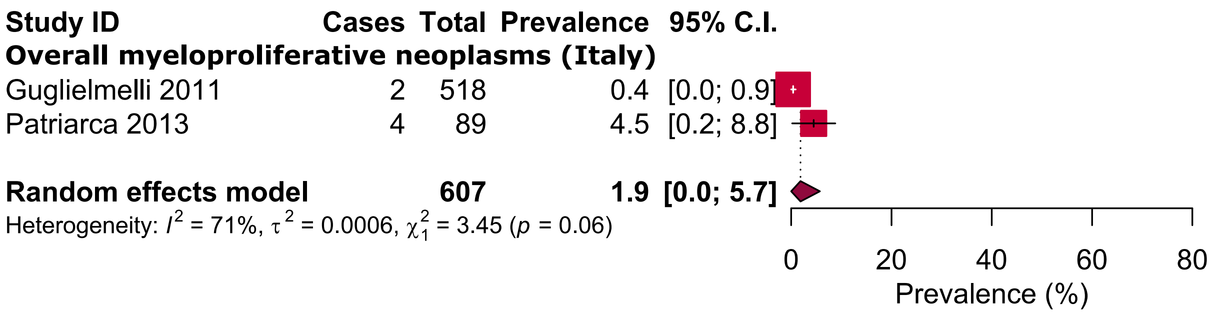
Study ID Cases Total Prevalence 95% C.I.
Overall myeloproliferative neoplasms (Italy)
Guglielmelli 2011 2 518 0.4 [0.0; 0.9]
Patriarca 2013 4 89 4.5 [0.2; 8.8]
Random effects model 607 1.9 [0.0; 5.7]
Heterogeneity: I^2 = 71%, τ^2 = 0.0006, χ^2_1 = 3.45 (p = 0.06)
0 20 40 60 80
Prevalence (%)

E

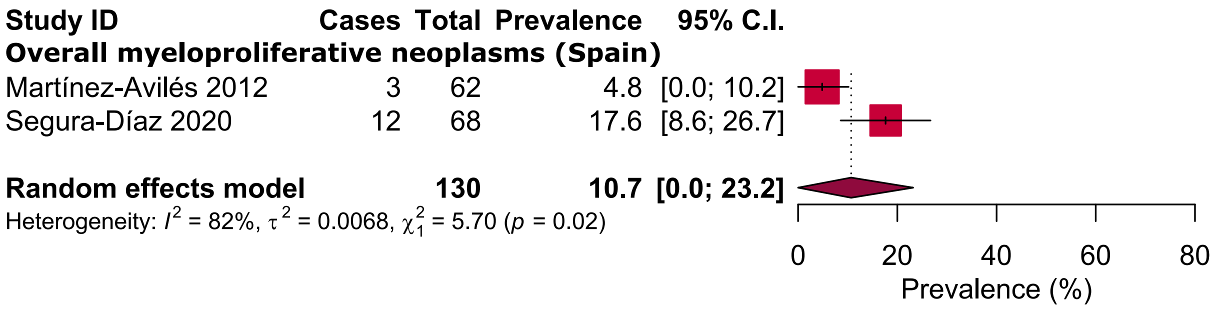
Study ID Cases Total Prevalence 95% C.I.
Overall myeloproliferative neoplasms (Spain)
Martínez-Avilés 2012 3 62 4.8 [0.0; 10.2]
Segura-Díaz 2020 12 68 17.6 [8.6; 26.7]
Random effects model 130 10.7 [0.0; 23.2]
Heterogeneity: I^2 = 82%, τ^2 = 0.0068, χ^2_1 = 5.70 (p = 0.02)
0 20 40 60 80
Prevalence (%)

F

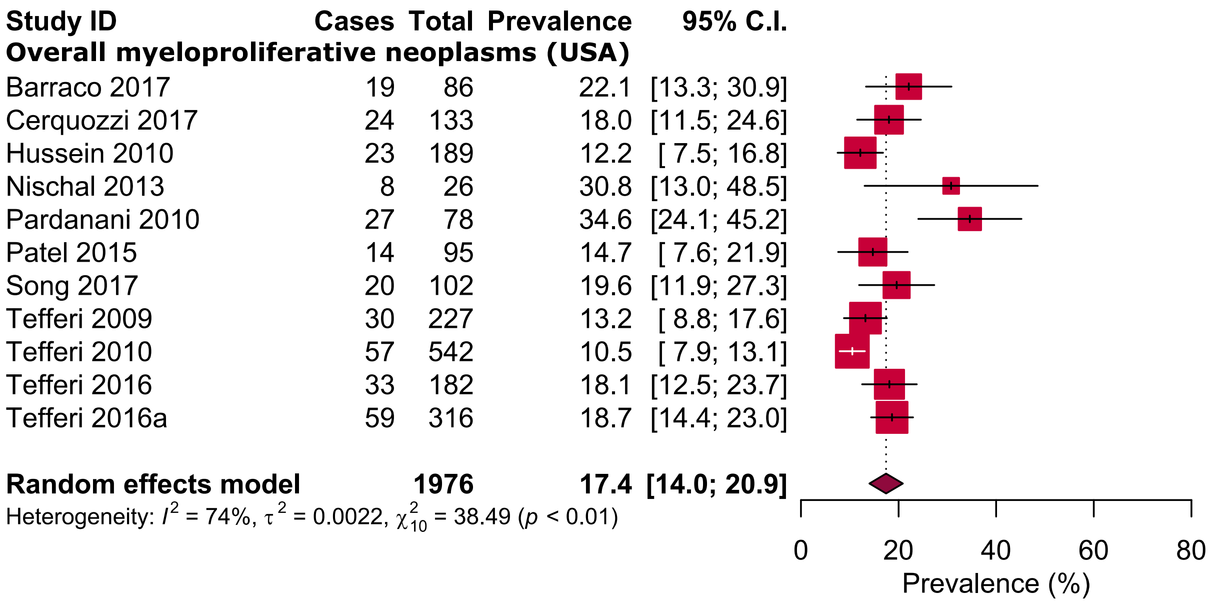
Study ID Cases Total Prevalence 95% C.I.
Overall myeloproliferative neoplasms (USA)
Barraco 2017 19 86 22.1 [13.3; 30.9]
Cerquozzi 2017 24 133 18.0 [11.5; 24.6]
Hussein 2010 23 189 12.2 [7.5; 16.8]
Nischal 2013 8 26 30.8 [13.0; 48.5]
Pardanani 2010 27 78 34.6 [24.1; 45.2]
Patel 2015 14 95 14.7 [7.6; 21.9]
Song 2017 20 102 19.6 [11.9; 27.3]
Tefferi 2009 30 227 13.2 [8.8; 17.6]
Tefferi 2010 57 542 10.5 [7.9; 13.1]
Tefferi 2016 33 182 18.1 [12.5; 23.7]
Tefferi 2016a 59 316 18.7 [14.4; 23.0]
Random effects model 1976 17.4 [14.0; 20.9]
Heterogeneity: I^2 = 74%, τ^2 = 0.0022, χ^2_{10} = 38.49 (p < 0.01)
0 20 40 60 80
Prevalence (%)

G

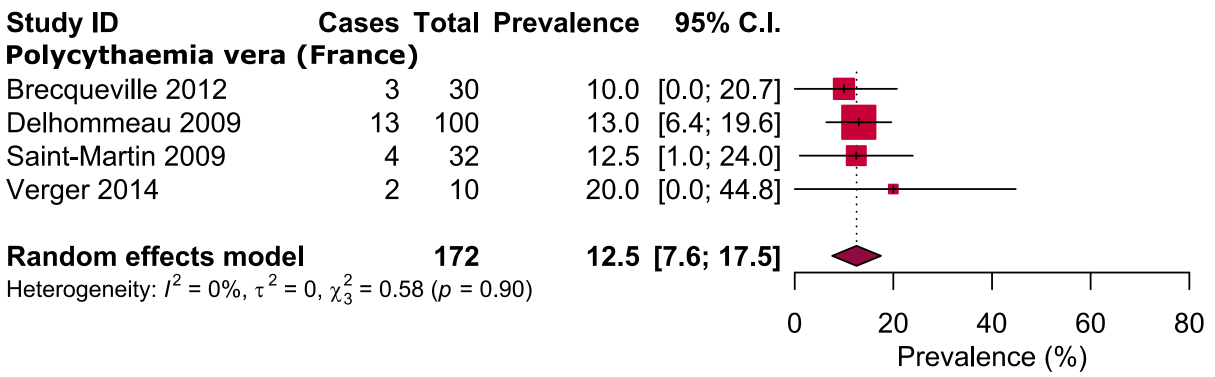
Study ID Cases Total Prevalence 95% C.I.
Polycythaemia vera (France)
Brecqueville 2012 3 30 10.0 [0.0; 20.7]
Delhommeau 2009 13 100 13.0 [6.4; 19.6]
Saint-Martin 2009 4 32 12.5 [1.0; 24.0]
Verger 2014 2 10 20.0 [0.0; 44.8]
Random effects model 172 12.5 [7.6; 17.5]
Heterogeneity: I^2 = 0%, τ^2 = 0, χ^2_3 = 0.58 (p = 0.90)
0 20 40 60 80
Prevalence (%)

H

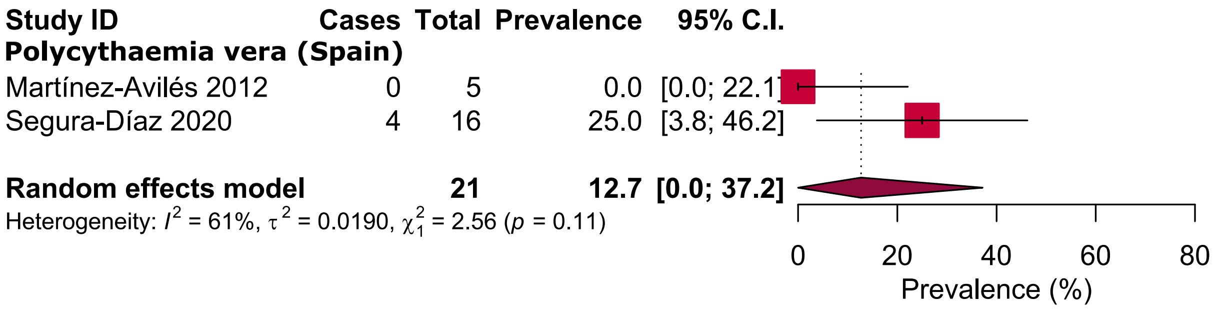
Study ID Cases Total Prevalence 95% C.I.
Polycythaemia vera (Spain)
Martínez-Avilés 2012 0 5 0.0 [0.0; 22.1]
Segura-Díaz 2020 4 16 25.0 [3.8; 46.2]
Random effects model 21 12.7 [0.0; 37.2]
Heterogeneity: I^2 = 61%, τ^2 = 0.0190, χ^2_1 = 2.56 (p = 0.11)
0 20 40 60 80
Prevalence (%)

I

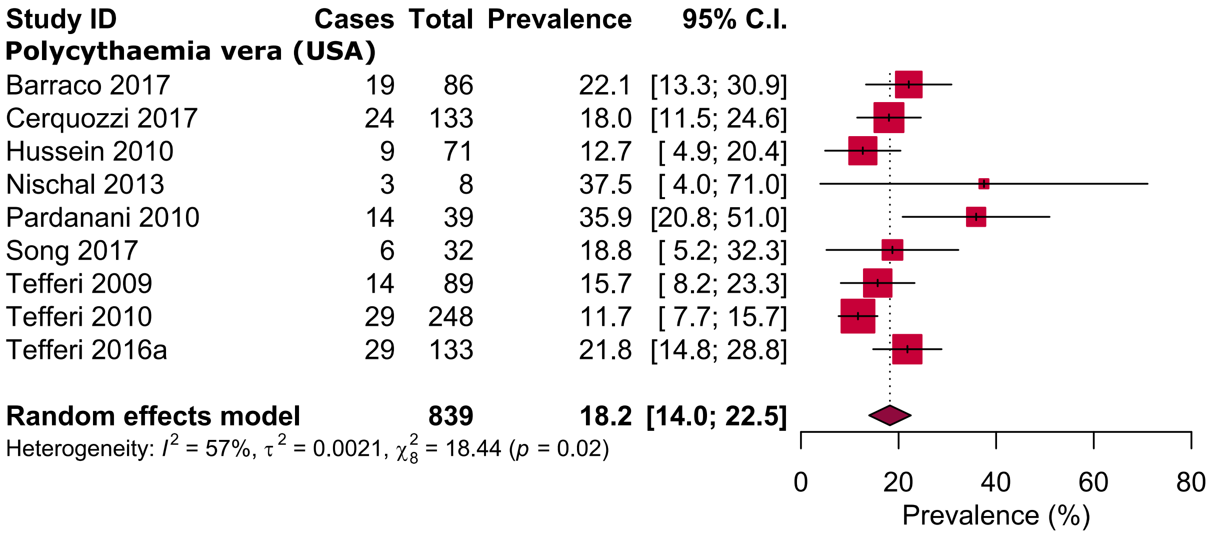
Study ID Cases Total Prevalence 95% C.I.
Polycythaemia vera (USA)
Barraco 2017 19 86 22.1 [13.3; 30.9]
Cerquozzi 2017 24 133 18.0 [11.5; 24.6]
Hussein 2010 9 71 12.7 [4.9; 20.4]
Nischal 2013 3 8 37.5 [4.0; 71.0]
Pardanani 2010 14 39 35.9 [20.8; 51.0]
Song 2017 6 32 18.8 [5.2; 32.3]
Tefferi 2009 14 89 15.7 [8.2; 23.3]
Tefferi 2010 29 248 11.7 [7.7; 15.7]
Tefferi 2016a 29 133 21.8 [14.8; 28.8]
Random effects model 839 18.2 [14.0; 22.5]
Heterogeneity: I^2 = 57%, τ^2 = 0.0021, χ^2_8 = 18.44 (p = 0.02)
0 20 40 60 80
Prevalence (%)

J

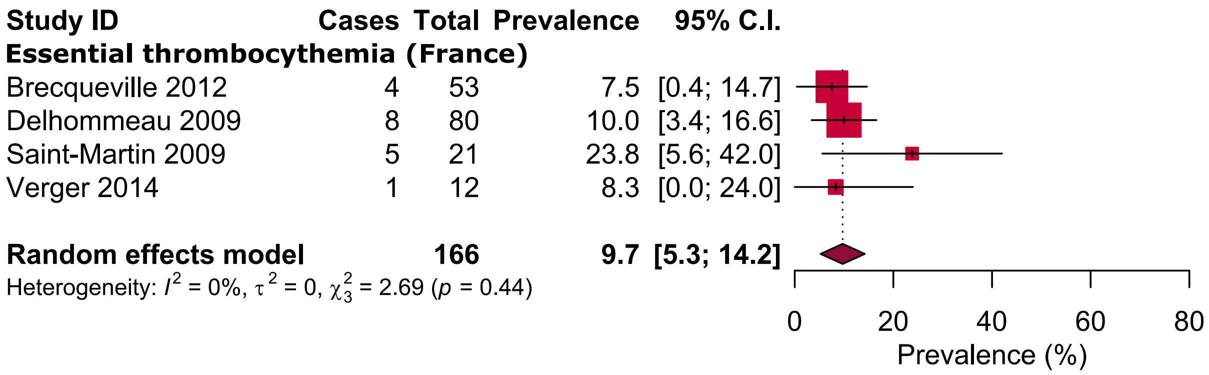
Study ID Cases Total Prevalence 95% C.I.
Essential thrombocythemia (France)
Brecqueville 2012 4 53 7.5 [0.4; 14.7]
Delhommeau 2009 8 80 10.0 [3.4; 16.6]
Saint-Martin 2009 5 21 23.8 [5.6; 42.0]
Verger 2014 1 12 8.3 [0.0; 24.0]
Random effects model 166 9.7 [5.3; 14.2]
Heterogeneity: I^2 = 0%, τ^2 = 0, χ^2_3 = 2.69 (p = 0.44)
0 20 40 60 80
Prevalence (%)

K

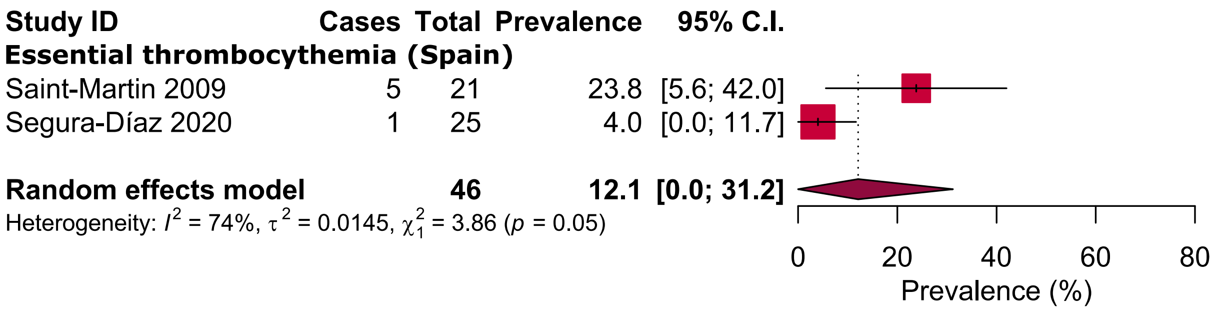
Study ID Cases Total Prevalence 95% C.I.
Essential thrombocythemia (Spain)
Saint-Martin 2009 5 21 23.8 [5.6; 42.0]
Segura-Díaz 2020 1 25 4.0 [0.0; 11.7]
Random effects model 46 12.1 [0.0; 31.2]
Heterogeneity: I^2 = 74%, τ^2 = 0.0145, χ^2_1 = 3.86 (p = 0.05)
0 20 40 60 80
Prevalence (%)

L

Study ID	Cases	Total	Prevalence	95% C.I.
**Essential thrombocythemia (USA)**				
Hussein 2010	1	43	2.3	[0.0; 6.8]
Nischal 2013	2	6	33.3	[0.0; 71.1]
Pardanani 2010	3	14	21.4	[0.0; 42.9]
Song 2017	2	27	7.4	[0.0; 17.3]
Tefferi 2009	3	57	5.3	[0.0; 11.1]
Tefferi 2010	13	177	7.3	[3.5; 11.2]
Tefferi 2016a	30	183	16.4	[11.0; 21.8]
**Random effects model**		**507**	**8.7**	**[3.8; 13.6]**

Heterogeneity: $I^2 = 69\%$, $\tau^2 = 0.0024$, $\chi^2_6 = 19.61$ ($p < 0.01$)

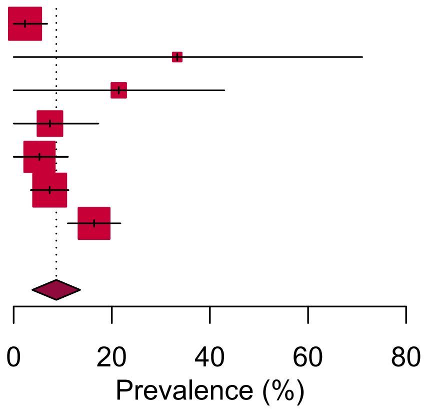

M

Study ID	Cases	Total	Prevalence	95% C.I.
**Myelofibrosis (China)**				
Gill 2018	18	101	17.8	[10.4; 25.3]
Huang 2020	2	6	33.3	[0.0; 71.1]
Zhang 2015	5	34	14.7	[2.8; 26.6]
**Random effects model**		**141**	**17.4**	**[11.2; 23.6]**

Heterogeneity: $I^2 = 0\%$, $\tau^2 = 0$, $\chi^2_2 = 0.89$ ($p = 0.64$)

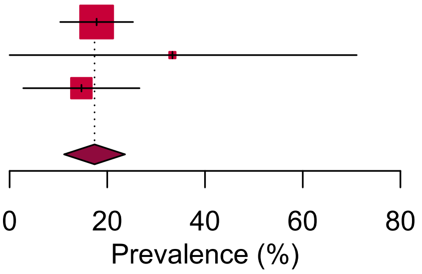

N

Study ID	Cases	Total	Prevalence	95% C.I.
**Myelofibrosis (France)**				
Brecqueville 2012	8	44	18.2	[6.8; 29.6]
Brecqueville 2014	11	68	16.2	[7.4; 24.9]
Delhommeau 2009	5	17	29.4	[7.8; 51.1]
Saint-Martin 2009	3	8	37.5	[4.0; 71.0]
Verger 2014	0	5	0.0	[0.0; 22.1]
**Random effects model**		**142**	**17.6**	**[9.9; 25.3]**

Heterogeneity: $I^2 = 20\%$, $\tau^2 = 0.0016$, $\chi^2_4 = 5.03$ ($p = 0.28$)

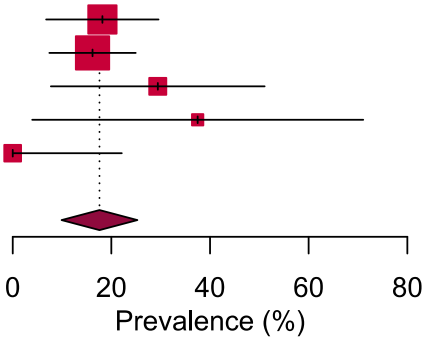

O

Study ID	Cases	Total	Prevalence	95% C.I.
**Myelofibrosis (Germany)**				
Bartels 2019	14	104	13.5	[6.9; 20.0]
Delic 2016	4	30	13.3	[1.2; 25.5]
Kröger 2017	16	169	9.5	[5.1; 13.9]
Schlenk 2016	10	96	10.4	[4.3; 16.5]
Schnittger 2012	3	11	27.3	[1.0; 53.6]
**Random effects model**		**410**	**11.0**	**[8.0; 14.0]**

Heterogeneity: $I^2 = 0\%$, $\tau^2 = 0$, $\chi^2_4 = 2.65$ ($p = 0.62$)

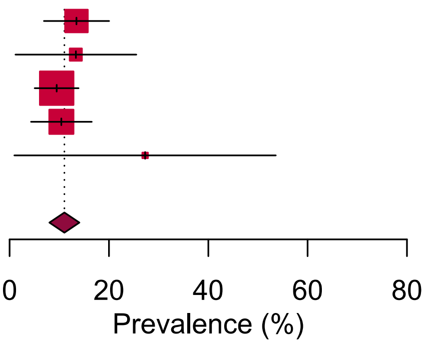

P

Study ID	Cases	Total	Prevalence	95% C.I.
**Myelofibrosis (Italy)**				
Guglielmelli 2011	2	518	0.4	[0; 0.9]
Patriarca 2013	0	9	0.0	[0; 13.5]
**Random effects model**		**527**	**0.4**	**[0; 0.9]**

Heterogeneity: $I^2 = 0\%$, $\tau^2 = 0$, $\chi^2_1 = 0.00$ ($p = 0.96$)

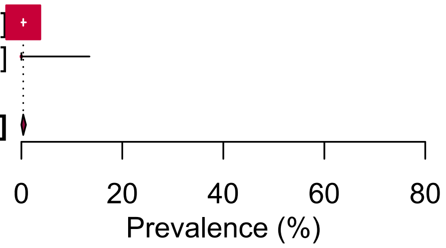

Q

Study ID	Cases	Total	Prevalence	95% C.I.
**Myelofibrosis (Spain)**				
Martínez-Avilés 2012	0	5	0.0	[0.0; 22.1]
Segura-Díaz 2020	7	27	25.9	[9.4; 42.5]
**Random effects model**		**32**	**14.0**	**[0.0; 39.4]**

Heterogeneity: $I^2 = 70\%$, $\tau^2 = 0.0237$, $\chi^2_1 = 3.39$ ($p = 0.07$)

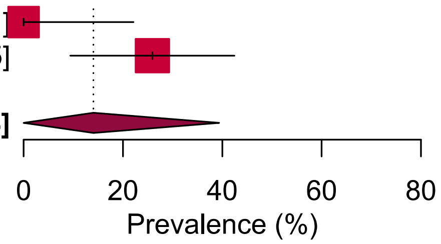

R

Study ID	Cases	Total	Prevalence	95% C.I.
**Myelofibrosis (USA)**				
Hussein 2010	13	75	17.3	[8.8; 25.9]
Nischal 2013	3	12	25.0	[0.5; 49.5]
Pardanani 2010	10	25	40.0	[20.8; 59.2]
Patel 2015	14	95	14.7	[7.6; 21.9]
Song 2017	12	44	27.3	[14.1; 40.4]
Tefferi 2009	13	81	16.0	[8.1; 24.0]
Tefferi 2010	15	117	12.8	[6.8; 18.9]
Tefferi 2016	33	182	18.1	[12.5; 23.7]
**Random effects model**		**631**	**17.7**	**[13.8; 21.6]**

Heterogeneity: $I^2 = 35\%$, $\tau^2 = 0.0010$, $\chi^2_7 = 10.69$ ($p = 0.15$)

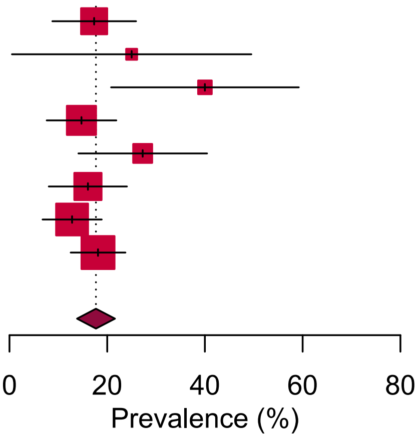

Figure S2. Prevalence of *TET2* gene mutations based on countries.

A

Study ID	Cases	Total	Prevalence	95% C.I.
**Overall myeloproliferative neoplasms (WHO criteria reported)**				
Andreasson 2020	26	85	30.6	[20.8; 40.4]
Barraco 2017	19	86	22.1	[13.3; 30.9]
Bartels 2019	14	104	13.5	[6.9; 20.0]
Brecqueville 2012	15	127	11.8	[6.2; 17.4]
Brecqueville 2014	11	68	16.2	[7.4; 24.9]
Cerquozzi 2017	24	133	18.0	[11.5; 24.6]
Delhommeau 2009	26	197	13.2	[8.5; 17.9]
Delic 2016	20	100	20.0	[12.2; 27.8]
Gill 2018	18	101	17.8	[10.4; 25.3]
Guglielmelli 2011	2	518	0.4	[0.0; 0.9]
Ha 2014	12	91	13.2	[6.2; 20.1]
Huang 2020	26	65	40.0	[28.1; 51.9]
Hussein 2010	23	189	12.2	[7.5; 16.8]
Leibundgut 2020	3	18	16.7	[0.0; 33.9]
Magor 2016	3	43	7.0	[0.0; 14.6]
Martínez-Avilés 2012	3	62	4.8	[0.0; 10.2]
Pardanani 2010	27	78	34.6	[24.1; 45.2]
Patriarca 2013	4	89	4.5	[0.2; 8.8]
Saint-Martin 2009	12	61	19.7	[9.7; 29.6]
Schlenk 2016	10	96	10.4	[4.3; 16.5]
Segura-Díaz 2020	12	68	17.6	[8.6; 26.7]
Song 2017	20	102	19.6	[11.9; 27.3]
Tefferi 2009	30	227	13.2	[8.8; 17.6]
Tefferi 2010	57	542	10.5	[7.9; 13.1]
Tefferi 2016	33	182	18.1	[12.5; 23.7]
Tefferi 2016a	59	316	18.7	[14.4; 23.0]
Zhang 2015	5	34	14.7	[2.8; 26.6]
**Random effects model**		**3782**	**15.7**	**[11.8; 19.7]**

Heterogeneity: $I^2 = 95\%$, $\tau^2 = 0.0095$, $\chi^2_{26} = 518.07$ ($p < 0.01$)

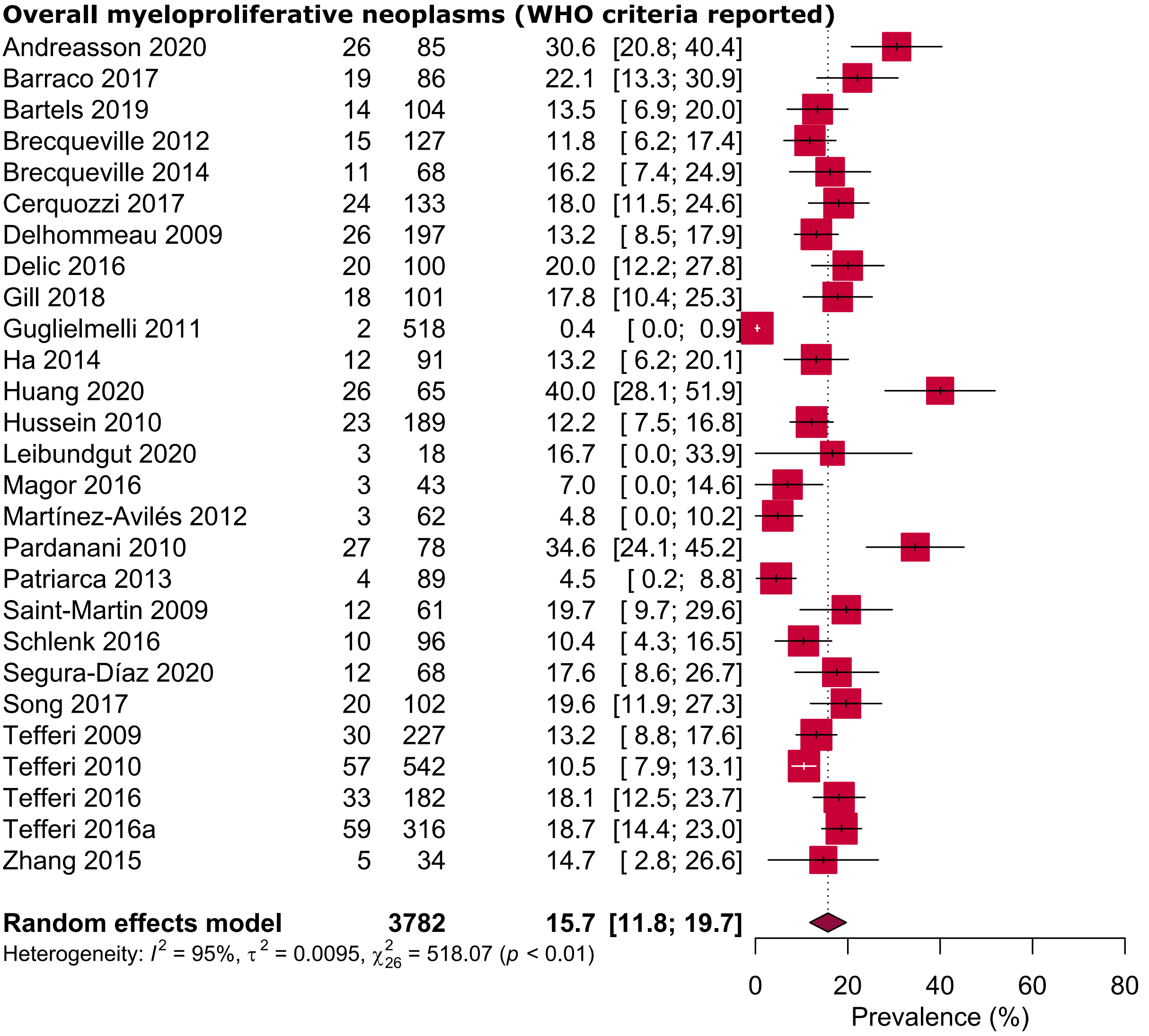

B

Study ID	Cases	Total	Prevalence	95% C.I.
**Overall myeloproliferative neoplasms (WHO criteria not reported)**				
Carbuccia 2009	4	55	7.3	[0.4; 14.1]
Kröger 2017	16	169	9.5	[5.1; 13.9]
Nielsen 2017	3	15	20.0	[0.0; 40.2]
Nischal 2013	8	26	30.8	[13.0; 48.5]
O'Sullivan 2019	12	110	10.9	[5.1; 16.7]
Patel 2015	14	95	14.7	[7.6; 21.9]
Schnittger 2012	11	41	26.8	[13.3; 40.4]
Verger 2014	3	27	11.1	[0.0; 23.0]
**Random effects model**		**538**	**13.1**	**[8.9; 17.3]**

Heterogeneity: $I^2 = 47\%$, $\tau^2 = 0.0015$, $\chi^2_7 = 13.22$ ($p = 0.07$)

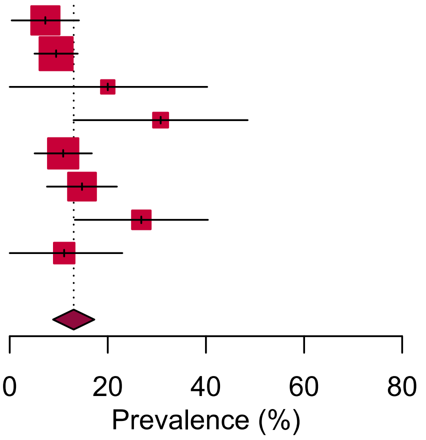

C

Study ID	Cases	Total	Prevalence	95% C.I.
**Overall myeloproliferative neoplasms (WHO 2001 criteria)**				
Delhommeau 2009	26	197	13.2	[8.5; 17.9]
Hussein 2010	23	189	12.2	[7.5; 16.8]
Tefferi 2009	30	227	13.2	[8.8; 17.6]
**Random effects model**		**613**	**12.9**	**[10.2; 15.5]**

Heterogeneity: $I^2 = 0\%$, $\tau^2 = 0$, $\chi^2_2 = 0.13$ ($p = 0.94$)

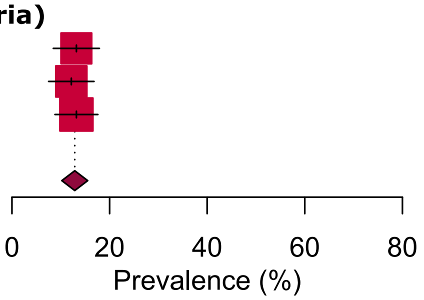

D

Study ID	Cases	Total	Prevalence	95% C.I.
**Overall myeloproliferative neoplasms (WHO 2008 criteria)**				
Andreasson 2020	26	85	30.6	[20.8; 40.4]
Brecqueville 2012	15	127	11.8	[6.2; 17.4]
Brecqueville 2014	11	68	16.2	[7.4; 24.9]
Delic 2016	20	100	20.0	[12.2; 27.8]
Guglielmelli 2011	2	518	0.4	[0.0; 0.9]
Ha 2014	12	91	13.2	[6.2; 20.1]
Magor 2016	3	43	7.0	[0.0; 14.6]
Martínez-Avilés 2012	3	62	4.8	[0.0; 10.2]
Pardanani 2010	27	78	34.6	[24.1; 45.2]
Patriarca 2013	4	89	4.5	[0.2; 8.8]
Saint-Martin 2009	12	61	19.7	[9.7; 29.6]
Schlenk 2016	10	96	10.4	[4.3; 16.5]
Song 2017	20	102	19.6	[11.9; 27.3]
Tefferi 2010	57	542	10.5	[7.9; 13.1]
Tefferi 2016	33	182	18.1	[12.5; 23.7]
Tefferi 2016a	59	316	18.7	[14.4; 23.0]
Zhang 2015	5	34	14.7	[2.8; 26.6]
**Random effects model**		**2594**	**14.5**	**[9.7; 19.3]**

Heterogeneity: $I^2 = 95\%$, $\tau^2 = 0.0089$, $\chi^2_{16} = 335.73$ ($p < 0.01$)

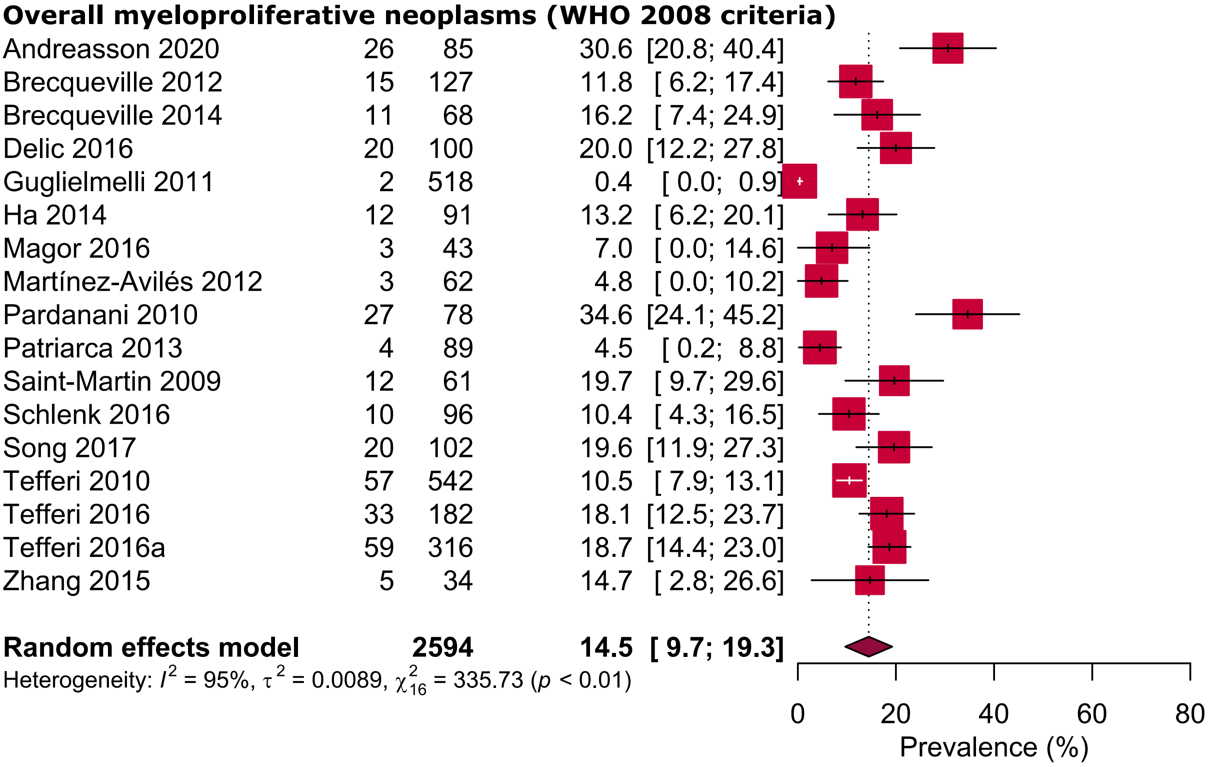

E

Study ID	Cases	Total	Prevalence	95% C.I.
**Overall myeloproliferative neoplasms (WHO 2016 criteria)**				
Barraco 2017	19	86	22.1	[13.3; 30.9]
Bartels 2019	14	104	13.5	[6.9; 20.0]
Cerquozzi 2017	24	133	18.0	[11.5; 24.6]
Gill 2018	18	101	17.8	[10.4; 25.3]
Huang 2020	26	65	40.0	[28.1; 51.9]
Leibundgut 2020	3	18	16.7	[0.0; 33.9]
Segura-Díaz 2020	12	68	17.6	[8.6; 26.7]
**Random effects model**		**575**	**20.1**	**[14.7; 25.4]**

Heterogeneity: $I^2 = 61\%$, $\tau^2 = 0.0030$, $\chi^2_6 = 15.49$ ($p = 0.02$)

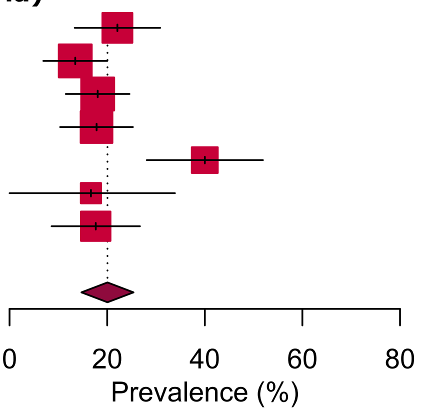

F

Study ID	Cases	Total	Prevalence	95% C.I.
**Polycythaemia vera (WHO 2001 criteria)**				
Delhommeau 2009	13	100	13.0	[6.4; 19.6]
Hussein 2010	9	71	12.7	[4.9; 20.4]
Tefferi 2009	14	89	15.7	[8.2; 23.3]
**Random effects model**		**260**	**13.7**	**[9.6; 17.9]**

Heterogeneity: $I^2 = 0\%$, $\tau^2 = 0$, $\chi^2_2 = 0.39$ ($p = 0.82$)

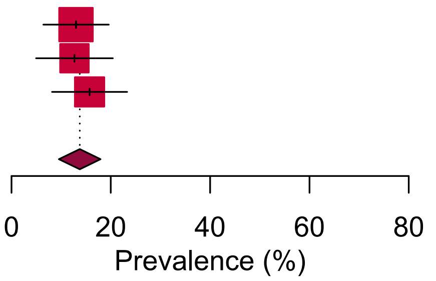

G

Study ID	Cases	Total	Prevalence	95% C.I.
**Polycythaemia vera (WHO 2008 criteria)**				
Andreasson 2020	26	85	30.6	[20.8; 40.4]
Brecqueville 2012	3	30	10.0	[0.0; 20.7]
Delic 2016	9	30	30.0	[13.6; 46.4]
Ha 2014	4	18	22.2	[3.0; 41.4]
Magor 2016	0	8	0.0	[0.0; 15.0]
Martínez-Avilés 2012	0	5	0.0	[0.0; 22.1]
Pardanani 2010	14	39	35.9	[20.8; 51.0]
Patriarca 2013	2	25	8.0	[0.0; 18.6]
Saint-Martin 2009	4	32	12.5	[1.0; 24.0]
Song 2017	6	32	18.8	[5.2; 32.3]
Tefferi 2010	29	248	11.7	[7.7; 15.7]
Tefferi 2016a	29	133	21.8	[14.8; 28.8]
**Random effects model**		**685**	**16.9**	**[11.3; 22.6]**

Heterogeneity: $I^2 = 69\%$, $\tau^2 = 0.0060$, $\chi^2_{11} = 35.63$ ($p < 0.01$)

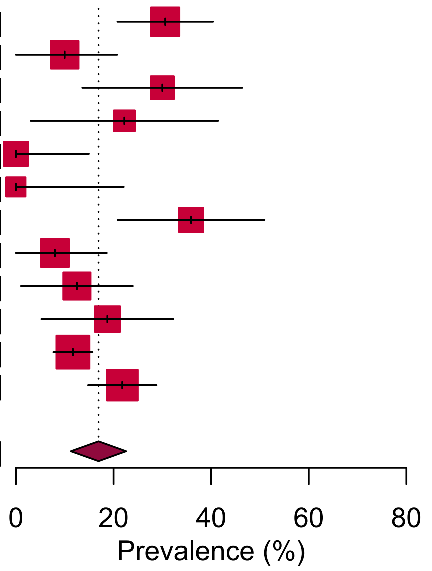

H

Study ID	Cases	Total	Prevalence	95% C.I.
**Polycythaemia vera (WHO 2016 criteria)**				
Barraco 2017	19	86	22.1	[13.3; 30.9]
Cerquozzi 2017	24	133	18.0	[11.5; 24.6]
Huang 2020	8	21	38.1	[17.3; 58.9]
Segura-Díaz 2020	4	16	25.0	[3.8; 46.2]
**Random effects model**		**256**	**21.4**	**[15.6; 27.3]**

Heterogeneity: $I^2 = 16\%$, $\tau^2 = 0.0007$, $\chi^2_3 = 3.58$ ($p = 0.31$)

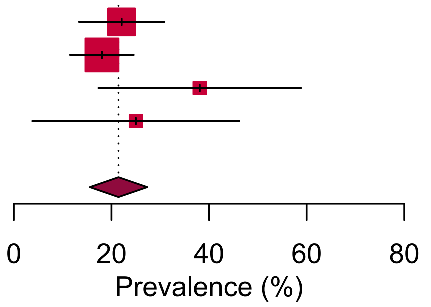

I

Study ID	Cases	Total	Prevalence	95% C.I.
**Essential thrombocythemia (WHO 2001 criteria)**				
Delhommeau 2009	8	80	10.0	[3.4; 16.6]
Hussein 2010	1	43	2.3	[0.0; 6.8]
Tefferi 2009	3	57	5.3	[0.0; 11.1]
**Random effects model**		**180**	**5.3**	**[1.1; 9.6]**

Heterogeneity: $I^2 = 44\%$, $\tau^2 = 0.0006$, $\chi^2_2 = 3.58$ ($p = 0.17$)

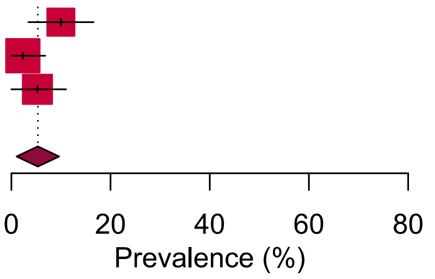

J

Study ID	Cases	Total	Prevalence	95% C.I.
**Essential thrombocythemia (WHO 2008 criteria)**				
Brecqueville 2012	4	53	7.5	[0.4; 14.7]
Delic 2016	7	40	17.5	[5.7; 29.3]
Ha 2014	6	62	9.7	[2.3; 17.0]
Magor 2016	1	16	6.2	[0.0; 18.1]
Martínez-Avilés 2012	3	52	5.8	[0.0; 12.1]
Pardanani 2010	3	14	21.4	[0.0; 42.9]
Patriarca 2013	2	55	3.6	[0.0; 8.6]
Saint-Martin 2009	5	21	23.8	[5.6; 42.0]
Song 2017	2	27	7.4	[0.0; 17.3]
Tefferi 2010	13	177	7.3	[3.5; 11.2]
Tefferi 2016a	30	183	16.4	[11.0; 21.8]
**Random effects model**		**700**	**9.4**	**[6.1; 12.6]**

Heterogeneity: $I^2 = 49\%$, $\tau^2 = 0.0013$, $\chi^2_{10} = 19.76$ ($p = 0.03$)

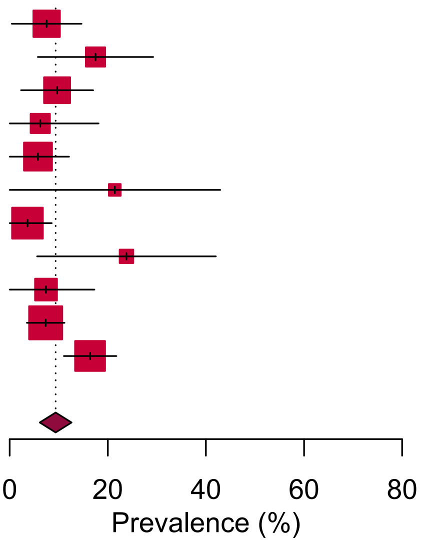

K

Study ID	Cases	Total	Prevalence	95% C.I.
**Essential thrombocythemia (WHO 2016 criteria)**				
Huang 2020	16	38	42.1	[26.4; 57.8]
Leibundgut 2020	3	18	16.7	[0.0; 33.9]
Segura-Díaz 2020	1	25	4.0	[0.0; 11.7]
**Random effects model**		**81**	**20.3**	**[0.0; 43.7]**

Heterogeneity: $I^2 = 89\%$, $\tau^2 = 0.0377$, $\chi^2_2 = 18.58$ ($p < 0.01$)

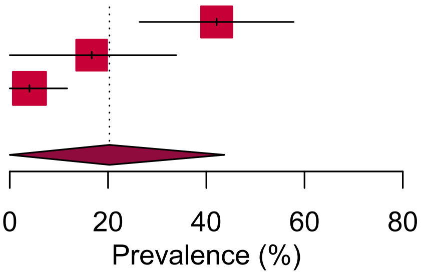

L

Study ID	Cases	Total	Prevalence	95% C.I.
**Myelofibrosis (WHO 2001 criteria)**				
Delhommeau 2009	5	17	29.4	[7.8; 51.1]
Hussein 2010	13	75	17.3	[8.8; 25.9]
Tefferi 2009	13	81	16.0	[8.1; 24.0]
**Random effects model**		**173**	**17.5**	**[11.9; 23.2]**

Heterogeneity: $I^2 = 0\%$, $\tau^2 = 0$, $\chi^2_2 = 1.29$ ($p = 0.52$)

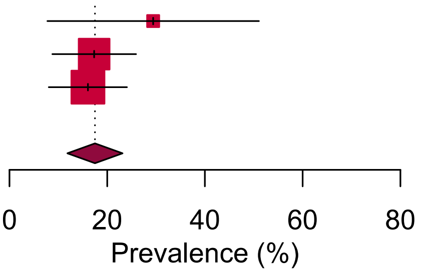

M

Study ID	Cases	Total	Prevalence	95% C.I.
**Myelofibrosis (WHO 2008 criteria)**				
Brecqueville 2012	8	44	18.2	[6.8; 29.6]
Brecqueville 2014	11	68	16.2	[7.4; 24.9]
Delic 2016	4	30	13.3	[1.2; 25.5]
Guglielmelli 2011	2	518	0.4	[0.0; 0.9]
Ha 2014	2	11	18.2	[0.0; 41.0]
Magor 2016	2	19	10.5	[0.0; 24.3]
Martínez-Avilés 2012	0	5	0.0	[0.0; 22.1]
Pardanani 2010	10	25	40.0	[20.8; 59.2]
Patriarca 2013	0	9	0.0	[0.0; 13.5]
Saint-Martin 2009	3	8	37.5	[4.0; 71.0]
Schlenk 2016	10	96	10.4	[4.3; 16.5]
Song 2017	12	44	27.3	[14.1; 40.4]
Tefferi 2010	15	117	12.8	[6.8; 18.9]
Tefferi 2016	33	182	18.1	[12.5; 23.7]
Zhang 2015	5	34	14.7	[2.8; 26.6]
**Random effects model**		**1210**	**14.4**	**[8.1; 20.7]**

Heterogeneity: $I^2 = 90\%$, $\tau^2 = 0.0110$, $\chi^2_{14} = 133.85$ ($p < 0.01$)

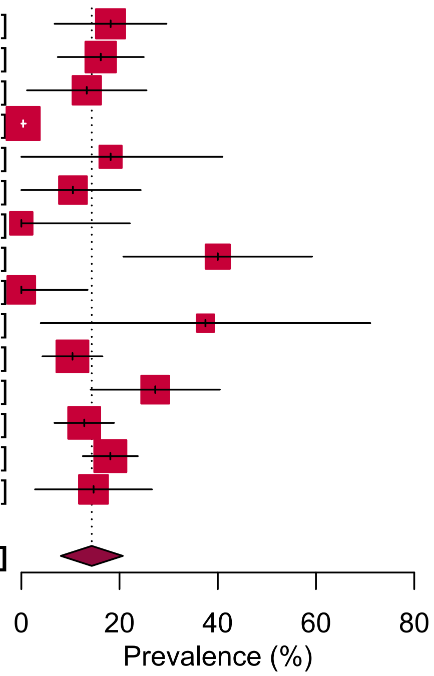

N

Study ID	Cases	Total	Prevalence	95% C.I.
**Myelofibrosis (WHO 2016 criteria)**				
Bartels 2019	14	104	13.5	[6.9; 20.0]
Gill 2018	18	101	17.8	[10.4; 25.3]
Huang 2020	2	6	33.3	[0.0; 71.1]
Segura-Díaz 2020	7	27	25.9	[9.4; 42.5]
**Random effects model**		**238**	**16.5**	**[11.8; 21.2]**

Heterogeneity: $I^2 = 0\%$, $\tau^2 = 0$, $\chi^2_3 = 2.96$ ($p = 0.40$)

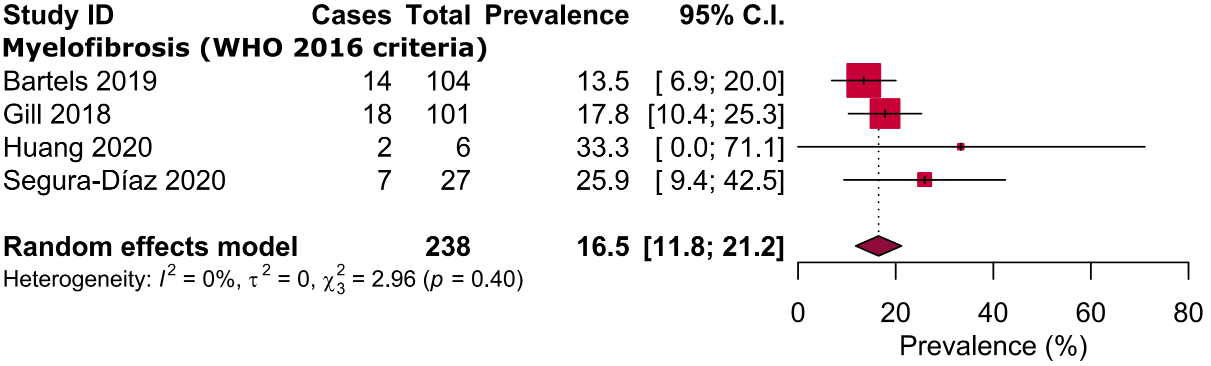

Figure S3. Prevalence of *TET2* gene mutations based on the WHO classification and diagnostic criteria of MPN.

A

Study ID	Cases	Total	Prevalence	95% C.I.
**Overall myeloproliferative neoplasms (NGS)**				
Andreasson 2020	26	85	30.6	[20.8; 40.4]
Bartels 2019	14	104	13.5	[6.9; 20.0]
Cerquozzi 2017	24	133	18.0	[11.5; 24.6]
Delic 2016	20	100	20.0	[12.2; 27.8]
Gill 2018	18	101	17.8	[10.4; 25.3]
Huang 2020	26	65	40.0	[28.1; 51.9]
Hussein 2010	23	189	12.2	[7.5; 16.8]
Leibundgut 2020	3	18	16.7	[0.0; 33.9]
O'Sullivan 2019	12	110	10.9	[5.1; 16.7]
Pardanani 2010	27	78	34.6	[24.1; 45.2]
Patel 2015	14	95	14.7	[7.6; 21.9]
Patriarca 2013	4	89	4.5	[0.2; 8.8]
Segura-Díaz 2020	12	68	17.6	[8.6; 26.7]
Song 2017	20	102	19.6	[11.9; 27.3]
Tefferi 2009	30	227	13.2	[8.8; 17.6]
Tefferi 2010	57	542	10.5	[7.9; 13.1]
Tefferi 2016	33	182	18.1	[12.5; 23.7]
Tefferi 2016a	59	316	18.7	[14.4; 23.0]
**Random effects model**		**2604**	**17.2**	**[14.0; 20.4]**

Heterogeneity: $I^2 = 80\%$, $\tau^2 = 0.0035$, $\chi^2_{17} = 85.62$ ($p < 0.01$)

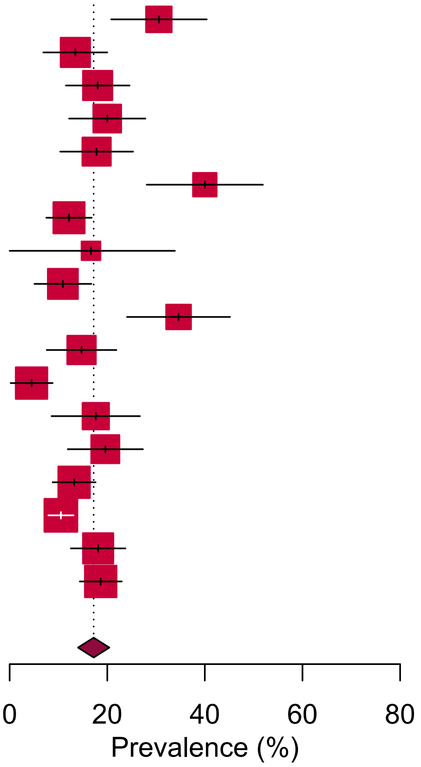

B

Study ID	Cases	Total	Prevalence	95% C.I.
**Overall myeloproliferative neoplasms (SS)**				
Brecqueville 2012	15	127	11.8	[6.2; 17.4]
Brecqueville 2014	11	68	16.2	[7.4; 24.9]
Delhommeau 2009	26	197	13.2	[8.5; 17.9]
Ha 2014	12	91	13.2	[6.2; 20.1]
Kröger 2017	16	169	9.5	[5.1; 13.9]
Martínez-Avilés 2012	3	62	4.8	[0.0; 10.2]
Nischal 2013	8	26	30.8	[13.0; 48.5]
Saint-Martin 2009	12	61	19.7	[9.7; 29.6]
Schlenk 2016	10	96	10.4	[4.3; 16.5]
Schnittger 2012	11	41	26.8	[13.3; 40.4]
Verger 2014	3	27	11.1	[0.0; 23.0]
**Random effects model**		**965**	**12.7**	**[9.6; 15.9]**

Heterogeneity: $I^2 = 52\%$, $\tau^2 = 0.0013$, $\chi^2_{10} = 20.74$ ($p = 0.02$)

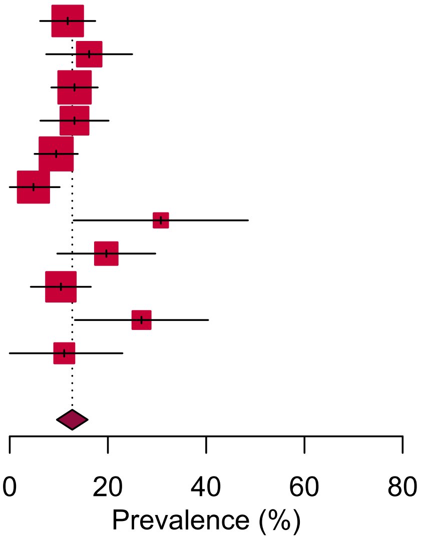

C

Study ID	Cases	Total	Prevalence	95% C.I.
**Overall myeloproliferative neoplasms (HRM)**				
Guglielmelli 2011	2	518	0.4	[0.0; 0.9]
Martínez-Avilés 2012	3	62	4.8	[0.0; 10.2]
Schnittger 2012	11	41	26.8	[13.3; 40.4]
**Random effects model**		**621**	**7.7**	**[0.0; 16.6]**

Heterogeneity: $I^2 = 88\%$, $\tau^2 = 0.0049$, $\chi^2_{2} = 17.18$ ($p < 0.01$)

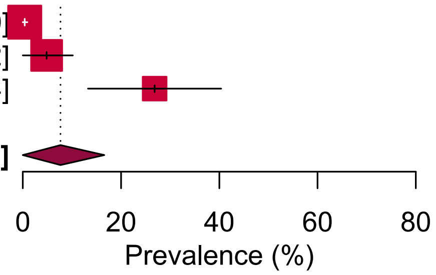

D

Study ID	Cases	Total	Prevalence	95% C.I.
**Polycythaemia vera (NGS)**				
Andreasson 2020	26	85	30.6	[20.8; 40.4]
Cerquozzi 2017	24	133	18.0	[11.5; 24.6]
Delic 2016	9	30	30.0	[13.6; 46.4]
Huang 2020	8	21	38.1	[17.3; 58.9]
Hussein 2010	9	71	12.7	[4.9; 20.4]
Pardanani 2010	14	39	35.9	[20.8; 51.0]
Patriarca 2013	2	25	8.0	[0.0; 18.6]
Segura-Díaz 2020	4	16	25.0	[3.8; 46.2]
Song 2017	6	32	18.8	[5.2; 32.3]
Tefferi 2009	14	89	15.7	[8.2; 23.3]
Tefferi 2010	29	248	11.7	[7.7; 15.7]
Tefferi 2016a	29	133	21.8	[14.8; 28.8]
**Random effects model**		**922**	**19.8**	**[15.1; 24.6]**

Heterogeneity: $I^2 = 67\%$, $\tau^2 = 0.0040$, $\chi^2_{11} = 33.13$ ($p < 0.01$)

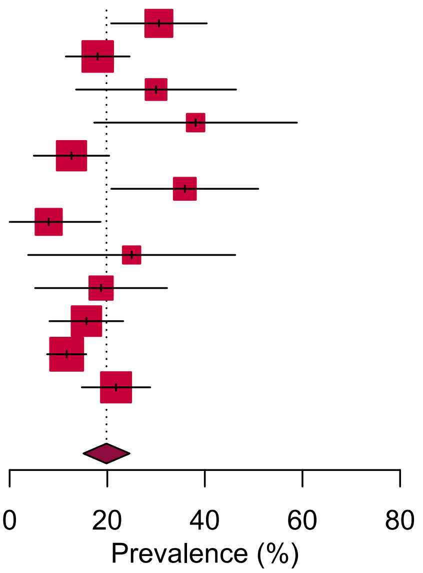

E

Study ID	Cases	Total	Prevalence	95% C.I.
**Polycythaemia vera (SS)**				
Brecqueville 2012	3	30	10.0	[0.0; 20.7]
Delhommeau 2009	13	100	13.0	[6.4; 19.6]
Ha 2014	4	18	22.2	[3.0; 41.4]
Martínez-Avilés 2012	0	5	0.0	[0.0; 22.1]
Nischal 2013	3	8	37.5	[4.0; 71.0]
Saint-Martin 2009	4	32	12.5	[1.0; 24.0]
Verger 2014	2	10	20.0	[0.0; 44.8]
**Random effects model**		**203**	**13.0**	**[8.4; 17.7]**

Heterogeneity: $I^2 = 0\%$, $\tau^2 = 0$, $\chi^2_6 = 4.88$ ($p = 0.56$)

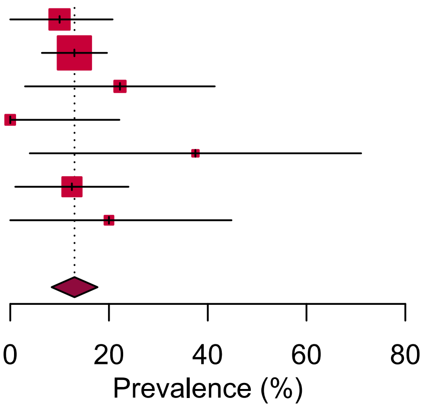

F

Study ID	Cases	Total	Prevalence	95% C.I.
**Polycythaemia vera (HRM)**				
Martínez-Avilés 2012	0	5	0.0	[0; 22.1]

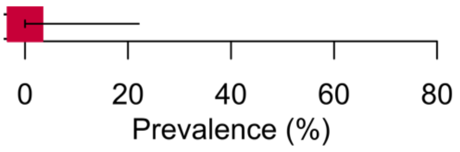

G

Study ID	Cases	Total	Prevalence	95% C.I.
**Essential thrombocythemia (NGS)**				
Delic 2016	7	40	17.5	[5.7; 29.3]
Huang 2020	16	38	42.1	[26.4; 57.8]
Hussein 2010	1	43	2.3	[0.0; 6.8]
Leibundgut 2020	3	18	16.7	[0.0; 33.9]
O'Sullivan 2019	12	110	10.9	[5.1; 16.7]
Pardanani 2010	3	14	21.4	[0.0; 42.9]
Patriarca 2013	2	55	3.6	[0.0; 8.6]
Segura-Díaz 2020	1	25	4.0	[0.0; 11.7]
Song 2017	2	27	7.4	[0.0; 17.3]
Tefferi 2009	3	57	5.3	[0.0; 11.1]
Tefferi 2010	13	177	7.3	[3.5; 11.2]
Tefferi 2016a	30	183	16.4	[11.0; 21.8]
**Random effects model**		**787**	**10.2**	**[6.1; 14.4]**

Heterogeneity: $I^2 = 75\%$, $\tau^2 = 0.0034$, $\chi^2_{11} = 44.57$ ($p < 0.01$)

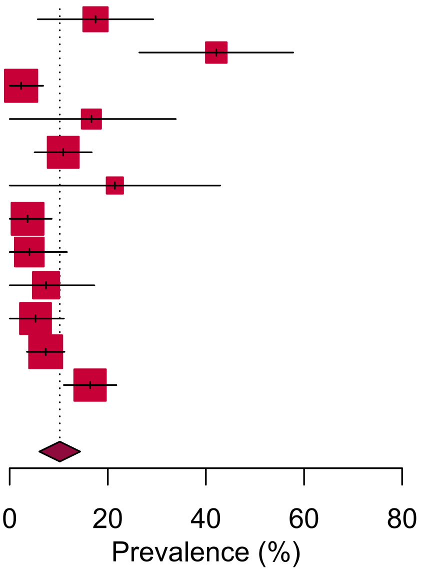

H

Study ID	Cases	Total	Prevalence	95% C.I.
**Essential thrombocythemia (SS)**				
Brecqueville 2012	4	53	7.5	[0.4; 14.7]
Delhommeau 2009	8	80	10.0	[3.4; 16.6]
Ha 2014	6	62	9.7	[2.3; 17.0]
Martínez-Avilés 2012	3	52	5.8	[0.0; 12.1]
Nischal 2013	2	6	33.3	[0.0; 71.1]
Saint-Martin 2009	5	21	23.8	[5.6; 42.0]
Schnittger 2012	8	30	26.7	[10.8; 42.5]
Verger 2014	1	12	8.3	[0.0; 24.0]
**Random effects model**		**316**	**10.4**	**[6.2; 14.6]**

Heterogeneity: $I^2 = 31\%$, $\tau^2 = 0.0010$, $\chi^2_7 = 10.09$ ($p = 0.18$)

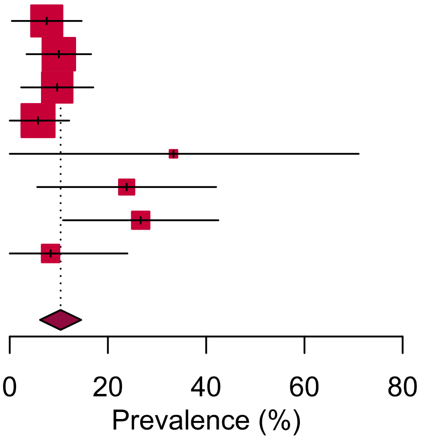

I

Study ID	Cases	Total	Prevalence	95% C.I.
**Essential thrombocythemia (HRM)**				
Martínez-Avilés 2012	3	52	5.8	[0.0; 12.1]
Schnittger 2012	8	30	26.7	[10.8; 42.5]
**Random effects model**		**82**	**14.9**	**[0.0; 35.2]**

Heterogeneity: $I^2 = 83\%$, $\tau^2 = 0.0181$, $\chi^2_1 = 5.77$ ($p = 0.02$)

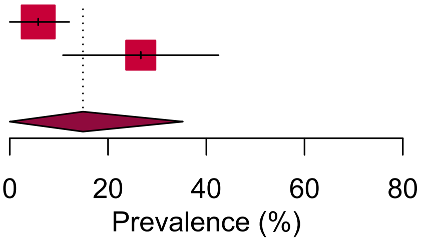

J

Study ID	Cases	Total	Prevalence	95% C.I.
**Myelofibrosis (NGS)**				
Bartels 2019	14	104	13.5	[6.9; 20.0]
Delic 2016	4	30	13.3	[1.2; 25.5]
Gill 2018	18	101	17.8	[10.4; 25.3]
Huang 2020	2	6	33.3	[0.0; 71.1]
Hussein 2010	13	75	17.3	[8.8; 25.9]
Pardanani 2010	10	25	40.0	[20.8; 59.2]
Patel 2015	14	95	14.7	[7.6; 21.9]
Patriarca 2013	0	9	0.0	[0.0; 13.5]
Segura-Díaz 2020	7	27	25.9	[9.4; 42.5]
Song 2017	12	44	27.3	[14.1; 40.4]
Tefferi 2009	13	81	16.0	[8.1; 24.0]
Tefferi 2010	15	117	12.8	[6.8; 18.9]
Tefferi 2016	33	182	18.1	[12.5; 23.7]
**Random effects model**		**896**	**16.5**	**[13.2; 19.8]**

Heterogeneity: $I^2 = 38\%$, $\tau^2 = 0.0012$, $\chi^2_{12} = 19.21$ ($p = 0.08$)

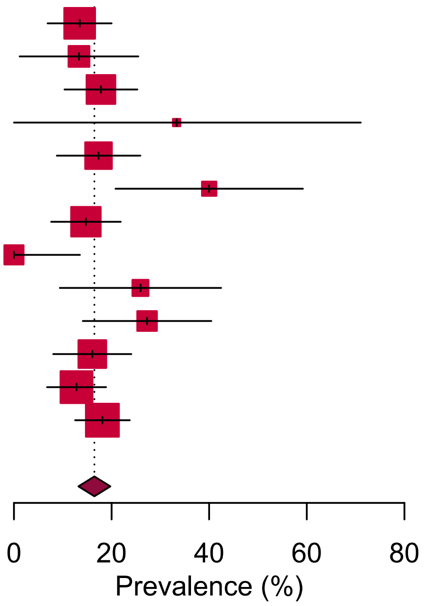

K

Study ID	Cases	Total	Prevalence	95% C.I.
**Myelofibrosis (SS)**				
Brecqueville 2012	8	44	18.2	[6.8; 29.6]
Brecqueville 2014	11	68	16.2	[7.4; 24.9]
Delhommeau 2009	5	17	29.4	[7.8; 51.1]
Ha 2014	2	11	18.2	[0.0; 41.0]
Kröger 2017	16	169	9.5	[5.1; 13.9]
Martínez-Avilés 2012	0	5	0.0	[0.0; 22.1]
Nischal 2013	3	12	25.0	[0.5; 49.5]
Saint-Martin 2009	3	8	37.5	[4.0; 71.0]
Schlenk 2016	10	96	10.4	[4.3; 16.5]
Schnittger 2012	3	11	27.3	[1.0; 53.6]
Verger 2014	0	5	0.0	[0.0; 22.1]
**Random effects model**		**446**	**13.3**	**[9.1; 17.5]**

Heterogeneity: $I^2 = 24\%$, $\tau^2 = 0.0010$, $\chi^2_{10} = 13.15$ ($p = 0.22$)

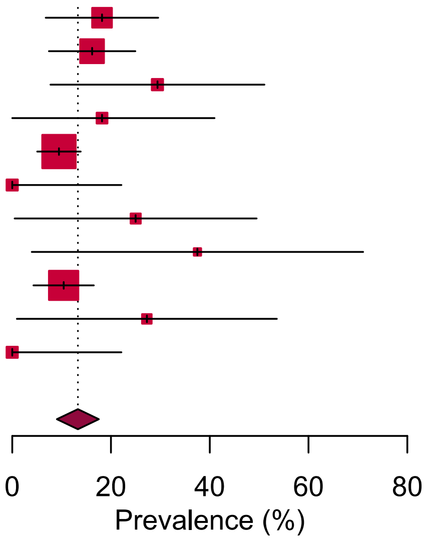

L

Study ID	Cases	Total	Prevalence	95% C.I.
**Myelofibrosis (HRM)**				
Guglielmelli 2011	2	518	0.4	[0; 0.9]
Martínez-Avilés 2012	0	5	0.0	[0; 22.1]
Schnittger 2012	3	11	27.3	[1; 53.6]
**Random effects model**		**534**	**5.0**	**[0; 18.2]**

Heterogeneity: $I^2 = 50\%$, $\tau^2 = 0.0075$, $\chi^2_{2} = 4.01$ ($p = 0.13$)

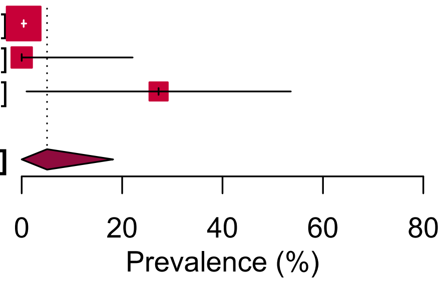

Figure S4. Prevalence of *TET2* gene mutations based on different methods used to detect *TET2* gene mutations in MPN.

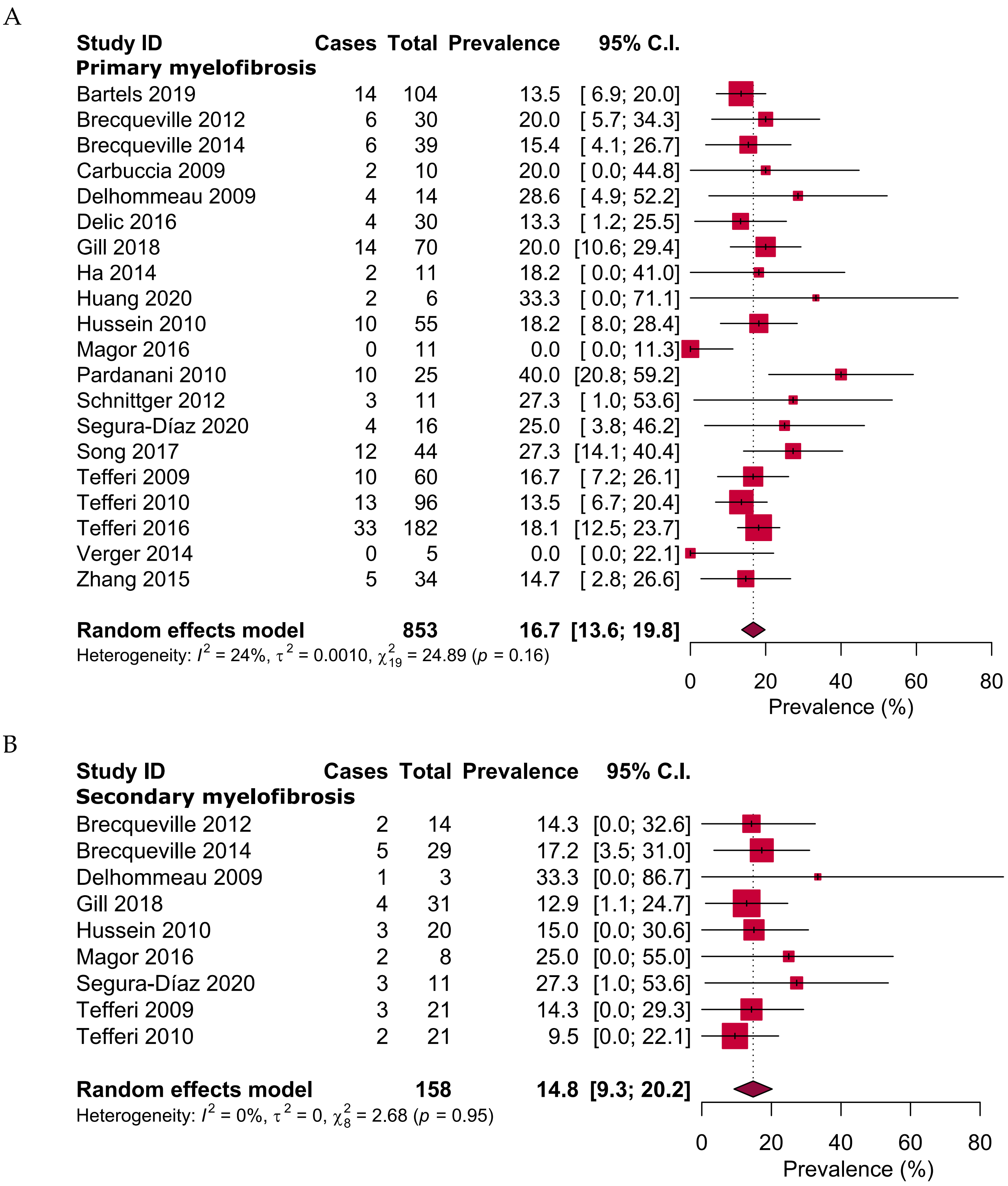

Figure S5. Subgroup analysis. Prevalence of *TET2* gene mutations in patients with (**A**) PMF and (**B**) SMF.

A

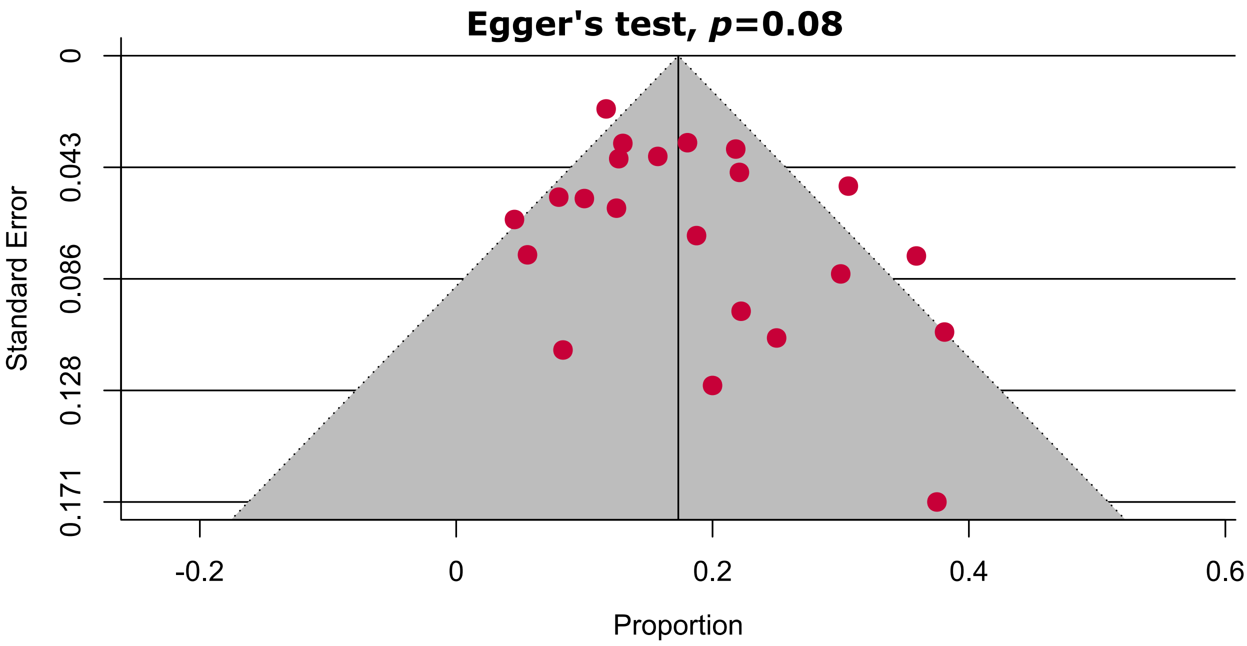

Egger's test, *p*=0.08
Standard Error
0
0.043
0.086
0.128
0.171
-0.2
0
0.2
0.4
0.6
Proportion

B

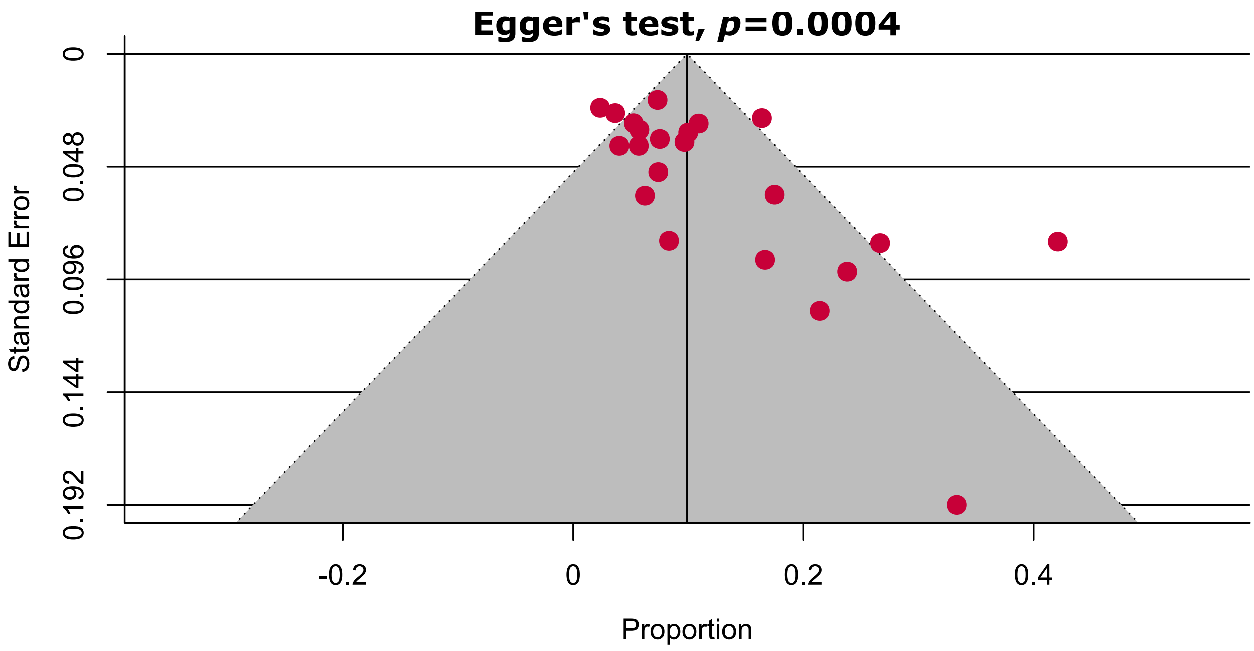

Egger's test, *p*=0.0004
Standard Error
0
0.048
0.096
0.144
0.192
-0.2
0
0.2
0.4
Proportion

C

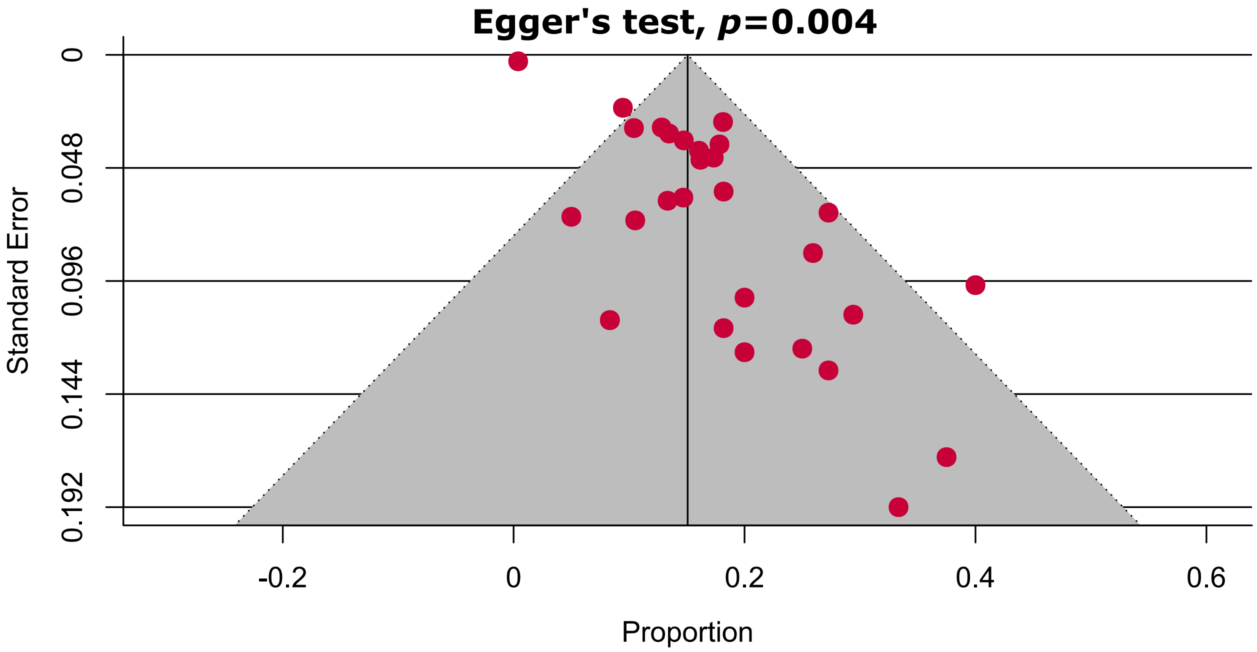

Figure S6. Funnel plots estimating the prevalence of *TET2* gene mutations in patients with (**A**) polycythaemia vera, (**B**) essential thrombocythaemia and (**C**) myelofibrosis.

A

Study ID	Cases	Total	Prevalence	95% C.I.
**Excluding small studies (MPNs, overall)**				
Bartels 2019	14	104	13.5	[6.9; 20.0]
Brecqueville 2012	15	127	11.8	[6.2; 17.4]
Cerquozzi 2017	24	133	18.0	[11.5; 24.6]
Delhommeau 2009	26	197	13.2	[8.5; 17.9]
Delic 2016	20	100	20.0	[12.2; 27.8]
Gill 2018	18	101	17.8	[10.4; 25.3]
Guglielmelli 2011	2	518	0.4	[0.0; 0.9]
Hussein 2010	23	189	12.2	[7.5; 16.8]
Kröger 2017	16	169	9.5	[5.1; 13.9]
O'Sullivan 2019	12	110	10.9	[5.1; 16.7]
Song 2017	20	102	19.6	[11.9; 27.3]
Tefferi 2009	30	227	13.2	[8.8; 17.6]
Tefferi 2010	57	542	10.5	[7.9; 13.1]
Tefferi 2016	33	182	18.1	[12.5; 23.7]
Tefferi 2016a	59	316	18.7	[14.4; 23.0]
**Random effects model**		**3117**	**13.6**	**[8.8; 18.4]**

Heterogeneity: I^2 = 96%, τ^2 = 0.0082, χ^2_{14} = 354.47 (p < 0.01)

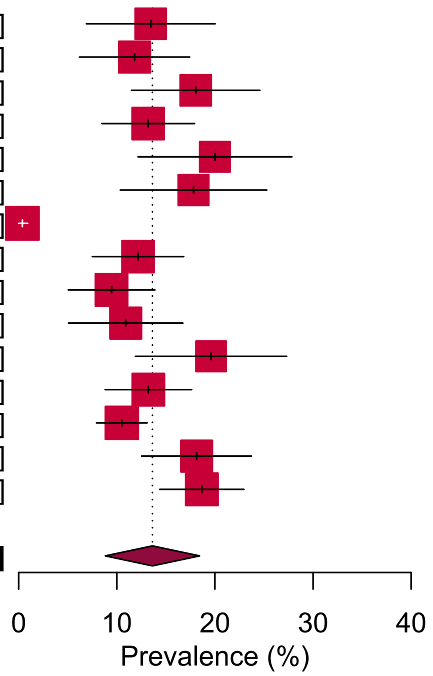

B

Study ID	Cases	Total	Prevalence	95% C.I.
**Excluding small studies (PV)**				
Cerquozzi 2017	24	133	18.0	[11.5; 24.6]
Delhommeau 2009	13	100	13.0	[6.4; 19.6]
Tefferi 2010	29	248	11.7	[7.7; 15.7]
Tefferi 2016a	29	133	21.8	[14.8; 28.8]
**Random effects model**		**614**	**15.6**	**[11.0; 20.3]**

Heterogeneity: I^2 = 59%, τ^2 = 0.0013, χ^2_3 = 7.37 (p = 0.06)

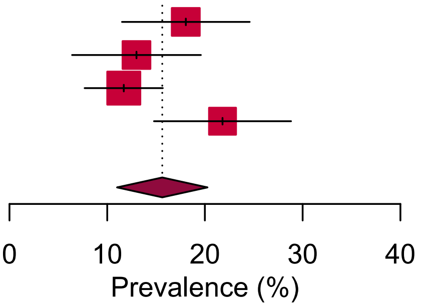

C

Study ID	Cases	Total	Prevalence	95% C.I.
**Excluding small studies (ET)**				
O'Sullivan 2019	12	110	10.9	[5.1; 16.7]
Tefferi 2010	13	177	7.3	[3.5; 11.2]
Tefferi 2016a	30	183	16.4	[11.0; 21.8]
**Random effects model**		**470**	**11.3**	**[5.9; 16.8]**

Heterogeneity: I^2 = 72%, τ^2 = 0.0017, χ^2_2 = 7.25 (p = 0.03)

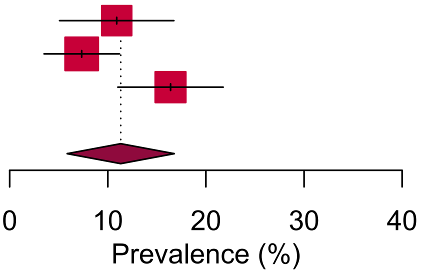

D

Study ID	Cases	Total	Prevalence	95% C.I.
**Excluding small studies (MF)**				
Bartels 2019	14	104	13.5	[6.9; 20.0]
Gill 2018	18	101	17.8	[10.4; 25.3]
Guglielmelli 2011	2	518	0.4	[0.0; 0.9]
Kröger 2017	16	169	9.5	[5.1; 13.9]
Tefferi 2010	15	117	12.8	[6.8; 18.9]
Tefferi 2016	33	182	18.1	[12.5; 23.7]
**Random effects model**		**1191**	**11.7**	**[4.0; 19.5]**

Heterogeneity: I^2 = 95%, τ^2 = 0.0086, χ^2_5 = 103.13 (p < 0.01)

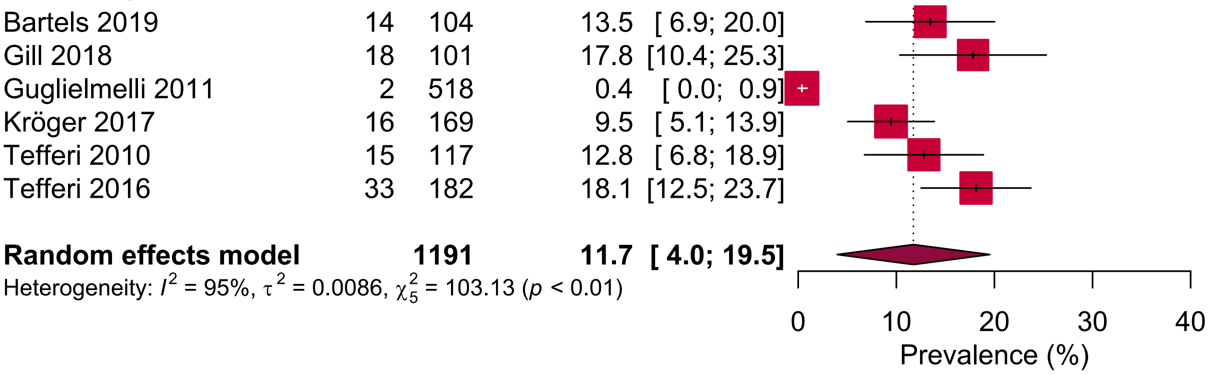

E

Study ID	Cases	Total	Prevalence	95% C.I.
**Excluding low- and moderate-quality studies (MPNs, overall)**				
Andreasson 2020	26	85	30.6	[20.8; 40.4]
Barraco 2017	19	86	22.1	[13.3; 30.9]
Bartels 2019	14	104	13.5	[6.9; 20.0]
Brecqueville 2012	15	127	11.8	[6.2; 17.4]
Brecqueville 2014	11	68	16.2	[7.4; 24.9]
Cerquozzi 2017	24	133	18.0	[11.5; 24.6]
Delic 2016	20	100	20.0	[12.2; 27.8]
Gill 2018	18	101	17.8	[10.4; 25.3]
Guglielmelli 2011	2	518	0.4	[0.0; 0.9]
Ha 2014	12	91	13.2	[6.2; 20.1]
Huang 2020	26	65	40.0	[28.1; 51.9]
Kröger 2017	16	169	9.5	[5.1; 13.9]
Leibundgut 2020	3	18	16.7	[0.0; 33.9]
O'Sullivan 2019	12	110	10.9	[5.1; 16.7]
Patel 2015	14	95	14.7	[7.6; 21.9]
Patriarca 2013	4	89	4.5	[0.2; 8.8]
Saint-Martin 2009	12	61	19.7	[9.7; 29.6]
Schlenk 2016	10	96	10.4	[4.3; 16.5]
Segura-Díaz 2020	12	68	17.6	[8.6; 26.7]
Tefferi 2009	30	227	13.2	[8.8; 17.6]
Tefferi 2010	57	542	10.5	[7.9; 13.1]
Tefferi 2016	33	182	18.1	[12.5; 23.7]
Tefferi 2016a	59	316	18.7	[14.4; 23.0]
Zhang 2015	5	34	14.7	[2.8; 26.6]
**Random effects model**		**3485**	**15.4**	**[11.3; 19.6]**

Heterogeneity: I^2 = 95%, τ^2 = 0.0091, χ^2_{23} = 454.90 (p < 0.01)

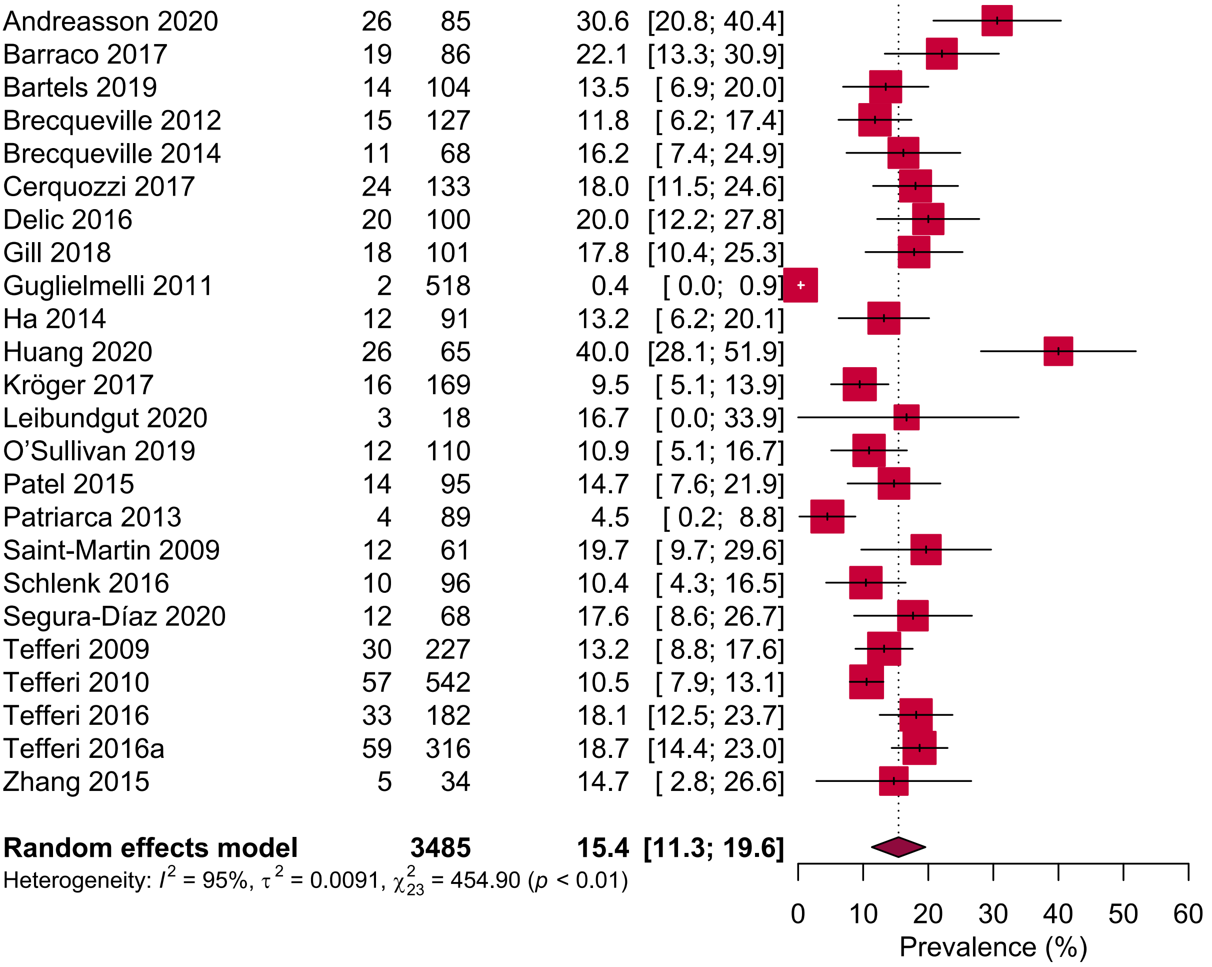

F

Study ID	Cases	Total	Prevalence	95% C.I.
**Excluding low- and moderate-quality studies (PV)**				
Andreasson 2020	26	85	30.6	[20.8; 40.4]
Barraco 2017	19	86	22.1	[13.3; 30.9]
Brecqueville 2012	3	30	10.0	[0.0; 20.7]
Cerquozzi 2017	24	133	18.0	[11.5; 24.6]
Delic 2016	9	30	30.0	[13.6; 46.4]
Ha 2014	4	18	22.2	[3.0; 41.4]
Huang 2020	8	21	38.1	[17.3; 58.9]
Patriarca 2013	2	25	8.0	[0.0; 18.6]
Saint-Martin 2009	4	32	12.5	[1.0; 24.0]
Segura-Díaz 2020	4	16	25.0	[3.8; 46.2]
Tefferi 2009	14	89	15.7	[8.2; 23.3]
Tefferi 2010	29	248	11.7	[7.7; 15.7]
Tefferi 2016a	29	133	21.8	[14.8; 28.8]
**Random effects model**		**946**	**18.6**	**[14.4; 22.7]**

Heterogeneity: $I^2 = 59\%$, $\tau^2 = 0.0030$, $\chi^2_{12} = 29.54$ ($p < 0.01$)

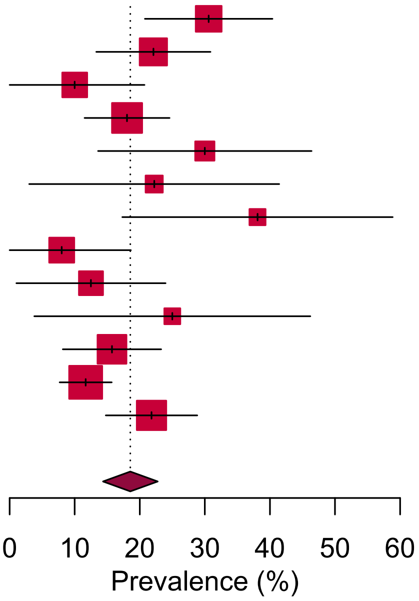

G

Study ID	Cases	Total	Prevalence	95% C.I.
**Excluding low- and moderate-quality studies (ET)**				
Brecqueville 2012	4	53	7.5	[0.4; 14.7]
Delic 2016	7	40	17.5	[5.7; 29.3]
Ha 2014	6	62	9.7	[2.3; 17.0]
Huang 2020	16	38	42.1	[26.4; 57.8]
Leibundgut 2020	3	18	16.7	[0.0; 33.9]
O'Sullivan 2019	12	110	10.9	[5.1; 16.7]
Patriarca 2013	2	55	3.6	[0.0; 8.6]
Saint-Martin 2009	5	21	23.8	[5.6; 42.0]
Segura-Díaz 2020	1	25	4.0	[0.0; 11.7]
Tefferi 2009	3	57	5.3	[0.0; 11.1]
Tefferi 2010	13	177	7.3	[3.5; 11.2]
Tefferi 2016a	30	183	16.4	[11.0; 21.8]
**Random effects model**		**839**	**11.1**	**[7.1; 15.0]**

Heterogeneity: $I^2 = 72\%$, $\tau^2 = 0.0030$, $\chi^2_{11} = 38.72$ ($p < 0.01$)

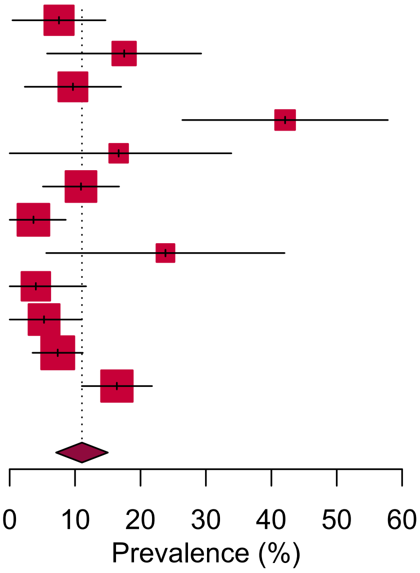

H

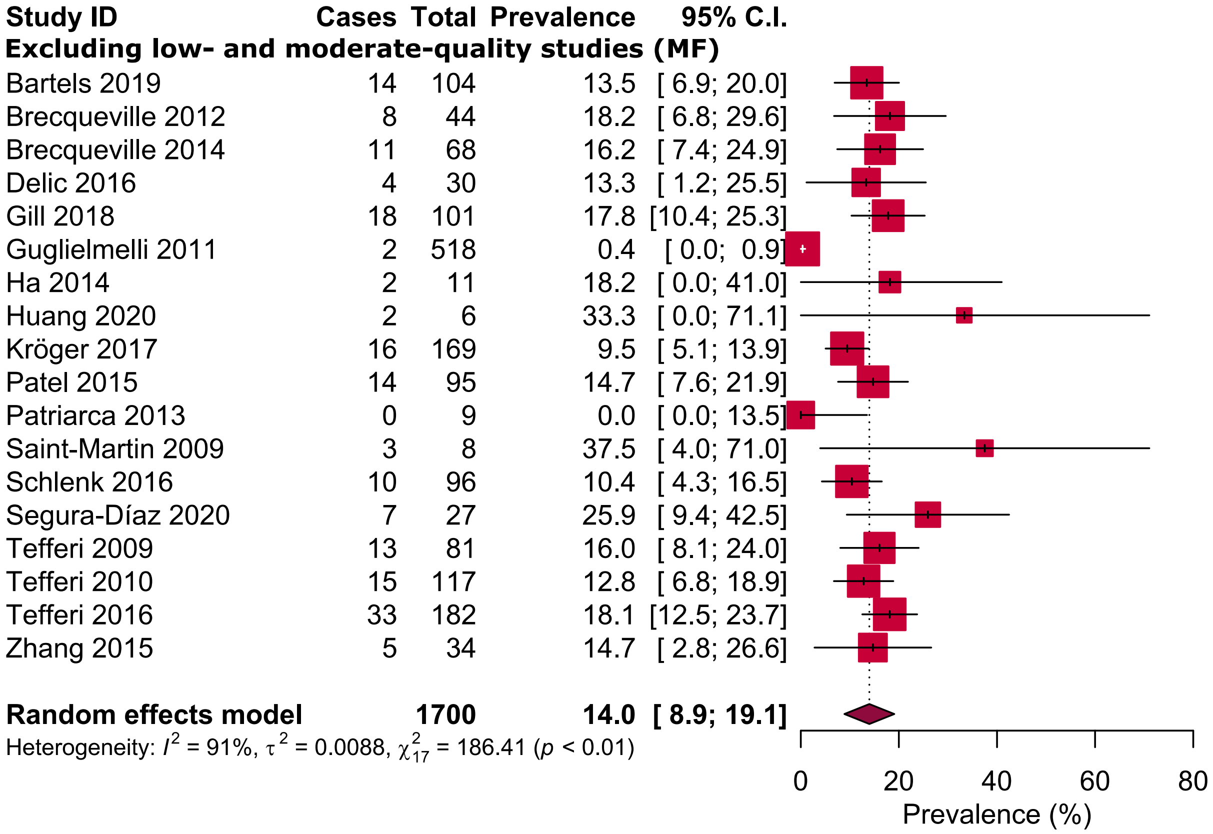
Study ID Cases Total Prevalence 95% C.I.
Excluding low- and moderate-quality studies (MF)
Bartels 2019 14 104 13.5 [6.9; 20.0]
Brecqueville 2012 8 44 18.2 [6.8; 29.6]
Brecqueville 2014 11 68 16.2 [7.4; 24.9]
Delic 2016 4 30 13.3 [1.2; 25.5]
Gill 2018 18 101 17.8 [10.4; 25.3]
Guglielmelli 2011 2 518 0.4 [0.0; 0.9]
Ha 2014 2 11 18.2 [0.0; 41.0]
Huang 2020 2 6 33.3 [0.0; 71.1]
Kröger 2017 16 169 9.5 [5.1; 13.9]
Patel 2015 14 95 14.7 [7.6; 21.9]
Patriarca 2013 0 9 0.0 [0.0; 13.5]
Saint-Martin 2009 3 8 37.5 [4.0; 71.0]
Schlenk 2016 10 96 10.4 [4.3; 16.5]
Segura-Díaz 2020 7 27 25.9 [9.4; 42.5]
Tefferi 2009 13 81 16.0 [8.1; 24.0]
Tefferi 2010 15 117 12.8 [6.8; 18.9]
Tefferi 2016 33 182 18.1 [12.5; 23.7]
Zhang 2015 5 34 14.7 [2.8; 26.6]
Random effects model 1700 14.0 [8.9; 19.1]
Heterogeneity: I^2 = 91%, τ^2 = 0.0088, χ^2_{17} = 186.41 (p < 0.01)
0
20
40
60
80
Prevalence (%)

I

Study ID	Cases	Total	Prevalence	95% C.I.
**Excluding outlier studies (MPNs, overall)**				
Andreasson 2020	26	85	30.6	[20.8; 40.4]
Barraco 2017	19	86	22.1	[13.3; 30.9]
Bartels 2019	14	104	13.5	[6.9; 20.0]
Brecqueville 2012	15	127	11.8	[6.2; 17.4]
Brecqueville 2014	11	68	16.2	[7.4; 24.9]
Carbuccia 2009	4	55	7.3	[0.4; 14.1]
Cerquozzi 2017	24	133	18.0	[11.5; 24.6]
Delhommeau 2009	26	197	13.2	[8.5; 17.9]
Delic 2016	20	100	20.0	[12.2; 27.8]
Gill 2018	18	101	17.8	[10.4; 25.3]
Ha 2014	12	91	13.2	[6.2; 20.1]
Hussein 2010	23	189	12.2	[7.5; 16.8]
Kröger 2017	16	169	9.5	[5.1; 13.9]
Leibundgut 2020	3	18	16.7	[0.0; 33.9]
Magor 2016	3	43	7.0	[0.0; 14.6]
Martínez-Avilés 2012	3	62	4.8	[0.0; 10.2]
Nielsen 2017	3	15	20.0	[0.0; 40.2]
O’Sullivan 2019	12	110	10.9	[5.1; 16.7]
Patel 2015	14	95	14.7	[7.6; 21.9]
Patriarca 2013	4	89	4.5	[0.2; 8.8]
Saint-Martin 2009	12	61	19.7	[9.7; 29.6]
Schlenk 2016	10	96	10.4	[4.3; 16.5]
Schnittger 2012	11	41	26.8	[13.3; 40.4]
Segura-Díaz 2020	12	68	17.6	[8.6; 26.7]
Song 2017	20	102	19.6	[11.9; 27.3]
Tefferi 2009	30	227	13.2	[8.8; 17.6]
Tefferi 2010	57	542	10.5	[7.9; 13.1]
Tefferi 2016	33	182	18.1	[12.5; 23.7]
Tefferi 2016a	59	316	18.7	[14.4; 23.0]
Verger 2014	3	27	11.1	[0.0; 23.0]
Zhang 2015	5	34	14.7	[2.8; 26.6]
**Random effects model**		**3633**	**13.9**	**[12.0; 15.9]**

Heterogeneity: I^2 = 63%, τ^2 = 0.0016, χ^2_{30} = 80.90 (p < 0.01)

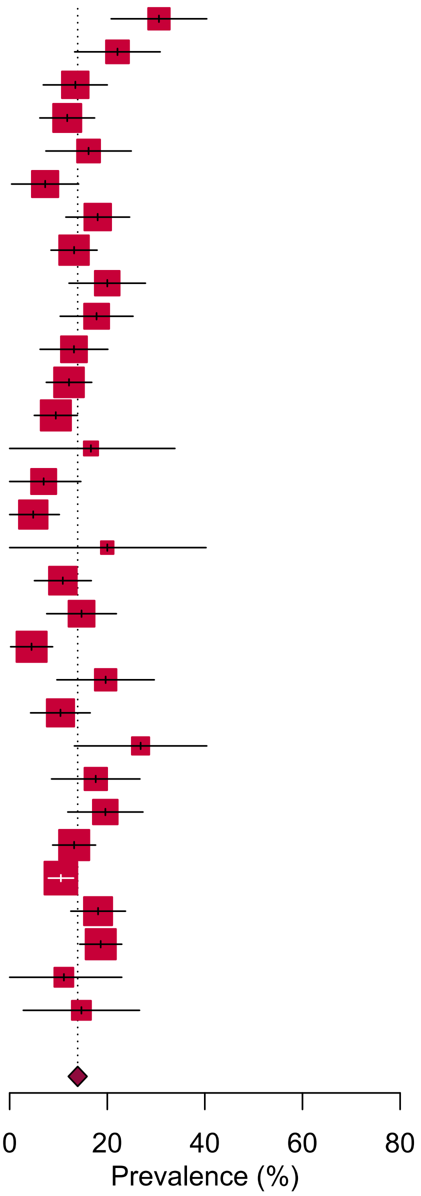

J

Study ID	Cases	Total	Prevalence	95% C.I.
**Excluding outlier studies (PV)**				
Andreasson 2020	26	85	30.6	[20.8; 40.4]
Barraco 2017	19	86	22.1	[13.3; 30.9]
Brecqueville 2012	3	30	10.0	[0.0; 20.7]
Carbuccia 2009	0	10	0.0	[0.0; 12.3]
Cerquozzi 2017	24	133	18.0	[11.5; 24.6]
Delhommeau 2009	13	100	13.0	[6.4; 19.6]
Delic 2016	9	30	30.0	[13.6; 46.4]
Ha 2014	4	18	22.2	[3.0; 41.4]
Hussein 2010	9	71	12.7	[4.9; 20.4]
Magor 2016	0	8	0.0	[0.0; 15.0]
Martínez-Avilés 2012	0	5	0.0	[0.0; 22.1]
Patriarca 2013	2	25	8.0	[0.0; 18.6]
Saint-Martin 2009	4	32	12.5	[1.0; 24.0]
Segura-Díaz 2020	4	16	25.0	[3.8; 46.2]
Song 2017	6	32	18.8	[5.2; 32.3]
Tefferi 2009	14	89	15.7	[8.2; 23.3]
Tefferi 2010	29	248	11.7	[7.7; 15.7]
Tefferi 2016a	29	133	21.8	[14.8; 28.8]
Verger 2014	2	10	20.0	[0.0; 44.8]
**Random effects model**		**1161**	**15.4**	**[12.0; 18.7]**

Heterogeneity: $I^2 = 54\%$, $\tau^2 = 0.0026$, $\chi^2_{18} = 39.12$ ($p < 0.01$)

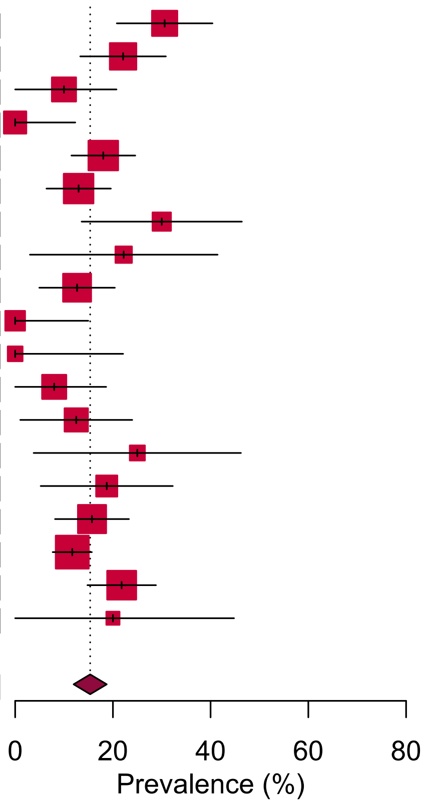

K

Study ID	Cases	Total	Prevalence	95% C.I.
**Excluding outlier studies (ET)**				
Brecqueville 2012	4	53	7.5	[0.4; 14.7]
Carbuccia 2009	2	35	5.7	[0.0; 13.4]
Delhommeau 2009	8	80	10.0	[3.4; 16.6]
Delic 2016	7	40	17.5	[5.7; 29.3]
Ha 2014	6	62	9.7	[2.3; 17.0]
Hussein 2010	1	43	2.3	[0.0; 6.8]
Leibundgut 2020	3	18	16.7	[0.0; 33.9]
Magor 2016	1	16	6.2	[0.0; 18.1]
Martínez-Avilés 2012	3	52	5.8	[0.0; 12.1]
O'Sullivan 2019	12	110	10.9	[5.1; 16.7]
Patriarca 2013	2	55	3.6	[0.0; 8.6]
Saint-Martin 2009	5	21	23.8	[5.6; 42.0]
Schnittger 2012	8	30	26.7	[10.8; 42.5]
Segura-Díaz 2020	1	25	4.0	[0.0; 11.7]
Song 2017	2	27	7.4	[0.0; 17.3]
Tefferi 2009	3	57	5.3	[0.0; 11.1]
Tefferi 2010	13	177	7.3	[3.5; 11.2]
Tefferi 2016a	30	183	16.4	[11.0; 21.8]
Verger 2014	1	12	8.3	[0.0; 24.0]
**Random effects model**		**1096**	**8.4**	**[6.0; 10.8]**

Heterogeneity: $I^2 = 48\%$, $\tau^2 = 0.0011$, $\chi^2_{18} = 34.57$ ($p = 0.01$)

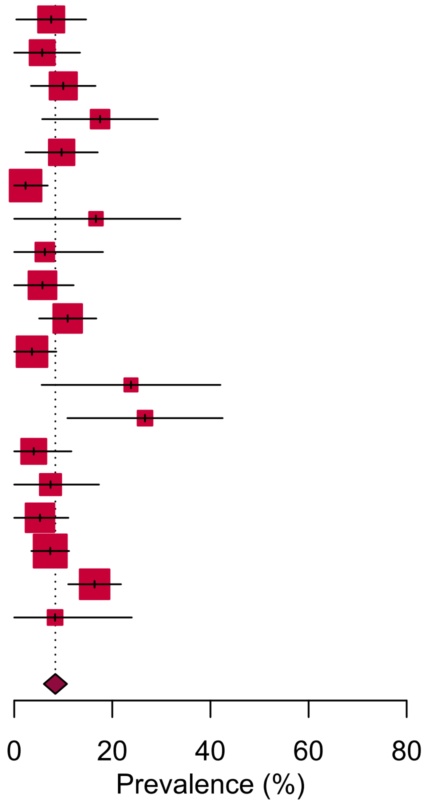

L

Study ID	Cases	Total	Prevalence	95% C.I.
**Excluding outlier studies (MF)**				
Bartels 2019	14	104	13.5	[6.9; 20.0]
Brecqueville 2012	8	44	18.2	[6.8; 29.6]
Brecqueville 2014	11	68	16.2	[7.4; 24.9]
Carbuccia 2009	2	10	20.0	[0.0; 44.8]
Delhommeau 2009	5	17	29.4	[7.8; 51.1]
Delic 2016	4	30	13.3	[1.2; 25.5]
Gill 2018	18	101	17.8	[10.4; 25.3]
Ha 2014	2	11	18.2	[0.0; 41.0]
Hussein 2010	13	75	17.3	[8.8; 25.9]
Kröger 2017	16	169	9.5	[5.1; 13.9]
Magor 2016	2	19	10.5	[0.0; 24.3]
Martínez-Avilés 2012	0	5	0.0	[0.0; 22.1]
Nielsen 2017	3	15	20.0	[0.0; 40.2]
Patel 2015	14	95	14.7	[7.6; 21.9]
Patriarca 2013	0	9	0.0	[0.0; 13.5]
Saint-Martin 2009	3	8	37.5	[4.0; 71.0]
Schlenk 2016	10	96	10.4	[4.3; 16.5]
Schnittger 2012	3	11	27.3	[1.0; 53.6]
Segura-Díaz 2020	7	27	25.9	[9.4; 42.5]
Song 2017	12	44	27.3	[14.1; 40.4]
Tefferi 2009	13	81	16.0	[8.1; 24.0]
Tefferi 2010	15	117	12.8	[6.8; 18.9]
Tefferi 2016	33	182	18.1	[12.5; 23.7]
Verger 2014	0	5	0.0	[0.0; 22.1]
Zhang 2015	5	34	14.7	[2.8; 26.6]
**Random effects model**		**1377**	**14.5**	**[12.4; 16.7]**

Heterogeneity: $I^2 = 18\%$, $\tau^2 = 0.0005$, $\chi^2_{24} = 29.12$ ($p = 0.22$)

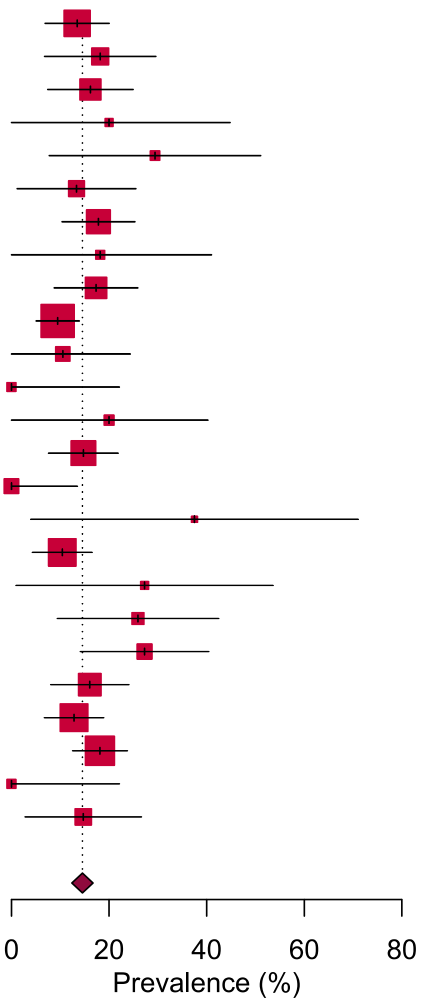

Figure S7. Sensitivity analyses.

References

1. Andréasson, B.; Pettersson, H.; Wasslavik, C.; Johansson, P.; Palmqvist, L.; Asp, J. ASXL1 mutations, previous vascular complications and age at diagnosis predict survival in 85 WHO-defined polycythaemia vera patients. *Br. J. Haematol.* **2020**, *189*, 913–919.
2. Barraco, D.; Cerquozzi, S.; Gangat, N.; Patnaik, M.M.; Lasho, T.; Finke, C.; Hanson, C.A.; Ketterling, R.P.; Pardanani, A.; Tefferi, A. Monocytosis in polycythemia vera: Clinical and molecular correlates. *Am. J. Hematol.* **2017**, *92*, 640–645.
3. Brecqueville, M.; Rey, J.; Bertucci, F.; Coppin, E.; Finetti, P.; Carbuccia, N.; Cervera, N.; Gelsi-Boyer, V.; Arnoulet, C.; Gisserot, O.; et al. Mutation analysis of ASXL1, CBL, DNMT3A, IDH1, IDH2, JAK2, MPL, NF1, SF3B1, SUZ12, and TET2 in myeloproliferative neoplasms. *Genes Chromosomes Cancer* **2012**, *51*, 743–755.
4. Brecqueville, M.; Rey, J.; Devillier, R.; Guille, A.; Gillet, R.; Adélaide, J.; Gelsi-Boyer, V.; Arnoulet, C.; Chaffanet, M.; Mozziconacci, M.J.; et al. Array comparative genomic hybridization and sequencing of 23 genes in 80 patients with myelofibrosis at chronic or acute phase. *Haematologica* **2014**, *99*, 37–45.
5. Carbuccia, N.; Murati, A.; Trouplin, V.; Brecqueville, M.; Adelaide, J.; Rey, J.; Vainchenker, W.; Bernard, O.; Chaffanet, M.; Vey, N. Mutations of ASXL1 gene in myeloproliferative neoplasms. *Leukemia* **2009**, *23*, 2183–2186.
6. Cerquozzi, S.; Barraco, D.; Lasho, T.; Finke, C.; Hanson, C.A.; Ketterling, R.P.; Pardanani, A.; Gangat, N.; Tefferi, A. Risk factors for arterial versus venous thrombosis in polycythemia vera: A single center experience in 587 patients. *Blood Cancer J.* **2017**, *7*, 662.
7. Delhommeau, F.; Dupont, S.; Della Valle, V.; James, C.; Trannoy, S.; Massé, A.; Kosmider, O.; Le Couedic, J.P.; Robert, F.; Alberdi, A.; et al. Mutation in TET2 in myeloid cancers. *N. Engl. J. Med.* **2009**, *360*, 2289–2301.
8. Delic, S.; Rose, D.; Kern, W.; Nadarajah, N.; Haferlach, C.; Haferlach, T.; Meggendorfer, M. Application of an NGS-based 28-gene panel in myeloproliferative neoplasms reveals distinct mutation patterns in essential thrombocythaemia, primary myelofibrosis and polycythaemia vera. *Br. J. Haematol.* **2016**, *175*, 419–426.

9. Gill, H.; Ip, H.W.; Yim, R.; Tang, W.F.; Pang, H.H.; Lee, P.; Leung, G.M.K.; Li, J.; Tang, K.; So, J.C.C.; et al. Next-generation sequencing with a 54-gene panel identified unique mutational profile and prognostic markers in Chinese patients with myelofibrosis. *Ann. Hematol.* **2019**, *98*, 869–879.
10. Guglielmelli, P.; Biamonte, F.; Score, J.; Hidalgo-Curtis, C.; Cervantes, F.; Maffioli, M.; Fanelli, T.; Ernst, T.; Winkelman, N.; Jones, A.V.; et al. EZH2 mutational status predicts poor survival in myelofibrosis. *Blood* **2011**, *118*, 5227–5234.
11. Ha, J.S.; Jeon, D.S.; Kim, J.R.; Ryoo, N.H.; Suh, J.S. Analysis of the ten-eleven translocation 2 (TET2) gene mutation in myeloproliferative neoplasms. *Ann. Clin. Lab. Sci.* **2014**, *44*, 173–179.
12. Huang, X.; Wu, J.; Deng, X.; Xu, X.; Zhang, X.; Ma, W.; Hu, T.; Yang, J.; Guan, M.; Tang, G. Mutation profiles of classic myeloproliferative neoplasms detected by a customized next-generation sequencing-based 50-gene panel. *J. Bio-X Res.* **2020**, *3*, 13–20.
13. Hussein, K.; Abdel-Wahab, O.; Lasho, T.L.; Van Dyke, D.L.; Levine, R.L.; Hanson, C.A.; Pardanani, A.; Tefferi, A. Cytogenetic correlates of TET2 mutations in 199 patients with myeloproliferative neoplasms. *Am. J. Hematol.* **2010**, *85*, 81.
14. Kröger, N.; Panagiota, V.; Badbaran, A.; Zabelina, T.; Triviai, I.; Araujo Cruz, M.M.; Shahswar, R.; Ayuk, F.; Gehlhaar, M.; Wolschke, C.; et al. Impact of Molecular Genetics on Outcome in Myelofibrosis Patients after Allogeneic Stem Cell Transplantation. *Biol. Blood Marrow Transplant.* **2017**, *23*, 1095–1101.
15. Oppliger Leibundgut, E.; Haubitz, M.; Burington, B.; Ottmann, O.G.; Spitzer, G.; Odenike, O.; McDevitt, M.A.; Röth, A.; Snyder, D.S.; Baerlocher, G.M. Dynamics of mutations in patients with essential thrombocythemia treated with imetelstat. *Haematologica* **2020**, doi:10.3324/haematol.2020.252817.
16. Magor, G.W.; Tallack, M.R.; Klose, N.M.; Taylor, D.; Korbie, D.; Mollee, P.; Trau, M.; Perkins, A.C. Rapid Molecular Profiling of Myeloproliferative Neoplasms Using Targeted Exon Resequencing of 86 Genes Involved in JAK-STAT Signaling and Epigenetic Regulation. *J. Mol. Diagn.* **2016**, *18*, 707–718.
17. Martinez-Aviles, L.; Besses, C.; Alvarez-Larran, A.; Torres, E.; Serrano, S.; Bellosillo, B. TET2, ASXL1, IDH1, IDH2, and c-CBL genes in JAK2-and MPL-negative myeloproliferative neoplasms. *Ann. Hematol.* **2012**, *91*, 533–541.
18. Nischal, S.; Bhattacharyya, S.; Christopeit, M.; Yu, Y.; Zhou, L.; Bhagat, T.D.; Sohal, D.; Will, B.; Mo, Y.; Suzuki, M.; et al. Methylome profiling reveals distinct alterations in phenotypic and mutational subgroups of myeloproliferative neoplasms. *Cancer Res.* **2013**, *73*, 1076–1085.
19. O'Sullivan, J.M.; Hamblin, A.; Yap, C.; Fox, S.; Boucher, R.; Panchal, A.; Alimam, S.; Dreau, H.; Howard, K.; Ware, P.; et al. The poor outcome in high molecular risk, hydroxycarbamide-resistant/intolerant ET is not ameliorated by ruxolitinib. *Blood* **2019**, *134*, 2107–2111.
20. Pardanani, A.; Lasho, T.; Finke, C.; Oh, S.T.; Gotlib, J.; Tefferi, A. LNK mutation studies in blast-phase myeloproliferative neoplasms, and in chronic-phase disease with TET2, IDH, JAK2 or MPL mutations. *Leukemia* **2010**, *24*, 1713–1718.
21. Patel, K.P.; Newberry, K.J.; Luthra, R.; Jabbour, E.; Pierce, S.; Cortes, J.; Singh, R.; Mehrotra, M.; Routbort, M.J.; Luthra, M.; et al. Correlation of mutation profile and response in patients with myelofibrosis treated with ruxolitinib. *Blood* **2015**, *126*, 790–797.
22. Patriarca, A.; Colaizzo, D.; Tiscia, G.; Spadano, R.; Di Zacomo, S.; Spadano, A.; Villanova, I.; Margaglione, M.; Grandone, E.; Dragani, A. TET2 mutations in Ph-negative myeloproliferative neoplasms: Identification of three novel mutations and relationship with clinical and laboratory findings. *BioMed Res. Int.* **2013**, *2013*, 929840.
23. Saint-Martin, C.; Leroy, G.; Delhommeau, F.; Panelatti, G.; Dupont, S.; James, C.; Plo, I.; Bordessoule, D.; Chomienne, C.; Delannoy, A.; et al. Analysis of the Ten-Eleven Translocation 2 (TET2) gene in familial myeloproliferative neoplasms. *Blood* **2009**, *114*, 1628–1632.
24. Schlenk, R.F.; Stegelmann, F.; Reiter, A.; Jost, E.; Gattermann, N.; Hebart, H.; Waller, C.; Hochhaus, A.; Platzbecker, U.; Schafhausen, P.; et al. Pomalidomide in myeloproliferative neoplasm-associated myelofibrosis. *Leukemia* **2017**, *31*, 889–895.
25. Schnittger, S.; Bacher, U.; Eder, C.; Dicker, F.; Alpermann, T.; Grossmann, V.; Kohlmann, A.; Kern, W.; Haferlach, C.; Haferlach, T. Molecular analyses of 15,542 patients with suspected BCR-ABL1-negative myeloproliferative disorders allow to develop a stepwise diagnostic workflow. *Haematologica* **2012**, *97*, 1582–1585.
26. Segura-Díaz, A.; Stuckey, R.; Florido, Y.; González-Martín, J.M.; López-Rodríguez, J.F.; Sánchez-Sosa, S.; González-Pérez, E.; Perdomo, M.N.S.; del Mar Perera, M.; de la Iglesia, S.; et al. Thrombotic risk detection in patients with polycythemia vera: The predictive role of DNMT3A/TET2/ASXL1 mutations. *Cancers* **2020**, *12*, 934.
27. Song, J.; Hussaini, M.; Zhang, H.; Shao, H.; Qin, D.; Zhang, X.; Ma, Z.; Hussnain Naqvi, S.M.; Zhang, L.; Moscinski, L.C. Comparison of the Mutational Profiles of Primary Myelofibrosis, Polycythemia Vera, and Essential Thrombocytosis. *Am. J. Clin. Pathol.* **2017**, *147*, 444–452.
28. Tefferi, A.; Pardanani, A.; Lim, K.H.; Abdel-Wahab, O.; Lasho, T.L.; Patel, J.; Gangat, N.; Finke, C.M.; Schwager, S.; Mullally, A.; et al. TET2 mutations and their clinical correlates in polycythemia vera, essential thrombocythemia and myelofibrosis. *Leukemia* **2009**, *23*, 905–911.
29. Tefferi, A.; Lasho, T.L.; Abdel-Wahab, O.; Guglielmelli, P.; Patel, J.; Caramazza, D.; Pieri, L.; Finke, C.M.; Kilpivaara, O.; Wadleigh, M.; et al. IDH1 and IDH2 mutation studies in 1473 patients with chronic-, fibrotic- or blast-phase essential thrombocythemia, polycythemia vera or myelofibrosis. *Leukemia* **2010**, *24*, 1302–1309.
30. Tefferi, A.; Lasho, T.L.; Finke, C.M.; Elala, Y.; Hanson, C.A.; Ketterling, R.P.; Gangat, N.; Pardanani, A. Targeted deep sequencing in primary myelofibrosis. *Blood Adv.* **2016**, *1*, 105–111.
31. Tefferi, A.; Lasho, T.L.; Guglielmelli, P.; Finke, C.M.; Rotunno, G.; Elala, Y.; Pacilli, A.; Hanson, C.A.; Pancrazzi, A.; Ketterling, R.P.; et al. Targeted deep sequencing in polycythemia vera and essential thrombocythemia. *Blood Adv.* **2016**, *1*, 21–30.

32. Verger, E.; Andreoli, A.; Chomienne, C.; Kiladjian, J.J.; Cassinat, B. TET2 gene sequencing may be helpful for myeloproliferative neoplasm diagnosis. *Br. J. Haematol.* **2014**, *165*, 416–419.
33. Chunxia, Z.; Li, G.; Qianqian, J.; Zhenling, L.; Fanzhou, H.; Yayue, G.; Ming, G.; Shaohua, X.; Yin, T.; Yanrong, C.; et al. Symptom burden and its relationships with risk assessment and gene mutations in patients with primary myelofibrosis. *J. Leuk. Lymphoma* **2015**, *24*, 453–456.
34. Bartels, S.; Faisal, M.; Büsche, G.; Schlue, J.; Hasemeier, B.; Schipper, E.; Vogtmann, J.; Westphal, L.; Lehmann, U.; Kreipe, H. Mutations associated with age-related clonal hematopoiesis in PMF patients with rapid progression to myelofibrosis. *Leukemia* **2019**, *34*, 1364–1372.
35. Myrtue Nielsen, H.; Lykkegaard Andersen, C.; Westman, M.; Sommer Kristensen, L.; Asmar, F.; Arvid Kruse, T.; Thomassen, M.; Stauffer Larsen, T.; Skov, V.; Lotte Hansen, L.; et al. Epigenetic changes in myelofibrosis: Distinct methylation changes in the myeloid compartments and in cases with ASXL1 mutations. *Sci. Rep.* **2017**, *7*, 6774.
